# Phospho-tau serine-262 and serine-356 as biomarkers of pre-tangle soluble tau assemblies in Alzheimer’s disease

**DOI:** 10.1038/s41591-024-03400-0

**Published:** 2025-02-10

**Authors:** Tohidul Islam, Emily Hill, Eric E. Abrahamson, Stijn Servaes, Denis S. Smirnov, Xuemei Zeng, Anuradha Sehrawat, Yijun Chen, Przemysław R. Kac, Hlin Kvartsberg, Maria Olsson, Emma Sjons, Fernando Gonzalez-Ortiz, Joseph Therriault, Cécile Tissot, Ivana Del Popolo, Nesrine Rahmouni, Abbie Richardson, Victoria Mitchell, Henrik Zetterberg, Tharick A. Pascoal, Tammaryn Lashley, Mark J. Wall, Douglas Galasko, Pedro Rosa-Neto, Milos D. Ikonomovic, Kaj Blennow, Thomas K. Karikari

**Affiliations:** 1 Department of Psychiatry and Neurochemistry, Institute of Neuroscience and Physiology, The Sahlgrenska Academy at the University of Gothenburg, Mölndal, Sweden; 2 School of Life Sciences, University of Warwick, Coventry, UK; 3 Department of Neurology, School of Medicine, University of Pittsburgh, Pittsburgh, PA USA; 4 Geriatric Research Education and Clinical Center, VA Pittsburgh HS, Pittsburgh, PA USA; 5 Translational Neuroimaging Laboratory, McGill University Research Centre for Studies in Aging, McConnell Brain Imaging Centre (BIC), Montréal Neurological Institute, Montréal, Québec Canada; 6 Department of Neurology and Neurosurgery, McGill University, Montréal, Québec Canada; 7 Shiley-Marcos Alzheimer’s Disease Research Center, Department of Neurosciences, University of California, San Diego, San Diego, CA USA; 8 Pathology Residency Program, Mass General and Brigham and Women’s Hospitals, Harvard Medical School, Boston, MA USA; 9 Department of Psychiatry, School of Medicine, University of Pittsburgh, Pittsburgh, PA USA; 10 Department of Chemistry, University of Pittsburgh, Pittsburgh, PA USA; 11 Clinical Neurochemistry Laboratory, Sahlgrenska University Hospital, Mölndal, Sweden; 12 Department of Neurodegenerative Diseases, Queen Square Institute of Neurology UCL, London, UK; 13 UK Dementia Research Institute, University College London, London, UK; 14 Hong Kong Center for Neurodegenerative Diseases, Hong Kong, China; 15 Department of Medicine, School of Medicine and Public Health, University of Wisconsin–Madison, Madison, WI USA; 16 Department of Neurodegenerative Disease, Queen Square Institute of Neurology, UCL, London, UK

**Keywords:** Alzheimer's disease, Neurochemistry

## Abstract

Patients with Alzheimer’s disease (AD) with little or no quantifiable insoluble brain tau neurofibrillary tangle (NFT) pathology demonstrate stronger clinical benefits of therapies than those with advanced NFTs. The formation of NFTs can be prevented by targeting the intermediate soluble tau assemblies (STAs). However, biochemical understanding and biomarkers of STAs are lacking. We show that Tris-buffered saline-soluble tau aggregates from autopsy-verified AD brain tissues include the core sequence ~tau_258–368_. In neuropathological assessments, antibodies against the phosphorylation sites serine-262 and serine-356 within the STA core almost exclusively stained granular (that is, prefibrillar) tau aggregates in pre-NFTs while antibodies against phosphorylation at serine-202 and threonine-205 and threonine-231, outside the STA core, stained the entire spectrum of tau aggregates in pre-NFTs and mature NFTs, dystrophic neurites and neuropil threads in the hippocampus. Functionally, a recombinantly produced STA core peptide robustly altered neuronal excitability and synaptic transmission in mouse hippocampal brain slices. Furthermore, we developed a cerebrospinal fluid assay that differentiated STAs in AD from non-AD tauopathies, correlated with the severity of NFT burden and cognitive decline independently of amyloid beta deposition, and with tau positron emission tomography uptake across Braak NFT stages. Together, our findings inform about the status of early-stage tau aggregation, reveal aggregation-relevant phosphorylation epitopes in tau and offer a diagnostic biomarker and targeted therapeutic opportunities for AD.

## Abstract (web summary)

Two new phosphorylation sites, p-tau_262_ and p-tau_356_, detect the formation of prefibrillar tau aggregates and may serve as early-stage biofluid-based biomarkers of tau neurofibrillary tangle pathology in Alzheimer’s disease.

## Main

Neurofibrillary tangles (NFTs), consisting of paired helical filaments (PHFs) or straight filaments due to the polymerization of tau protein into fibrillar intracellular aggregates, are a defining neuropathological feature of Alzheimer’s disease (AD)^1–6^. In AD, the severity of tau pathology, assessed according to the Braak staging for NFTs^1^, is a stronger correlate and predictor of cognitive outcomes than amyloid beta (Aβ) plaques, another neuropathological hallmark of AD^7–11^. While neuropathological diagnosis of AD requires tau immunohistochemical staining for NFTs^1,2^, in vivo imaging using tau positron emission tomography (PET) facilitates clinical diagnosis^12,13^. Yet, these diagnostic approaches lack sensitivity for the early-stage tau changes that precede NFTs^14,15^. Advances in cryo-electron microscopy (cryo-EM) have enabled detailed biophysical characterization of tau filaments in NFTs^16,17^. However, therapeutic targeting of mature tau filaments has fundamental drug development challenges, including filaments being substantially less neurotoxic compared with early-stage structures such as phosphorylated oligomers^18–20^. Additionally, biomarker methods for quantifying early-stage prefibrillar tau aggregates in biofluids are lacking.

Converging evidence indicates that physiological buffer-soluble, low-order tau assemblies (for example, oligomers and protomers, collectively referred to hereafter as soluble tau assemblies (STAs)) that form in the initial stages of the tau aggregation cascade and constitute the building blocks for the higher-order fibrils and NFTs, are highly efficient in seeding and propagating tau toxicity^21–24^. These findings indicate that early-stage STAs may have utility as early diagnostic biomarkers and targets for effective anti-tau therapeutics^21,25^. For instance, in a recent phase 3 randomized controlled clinical trial of an anti-Aβ monoclonal antibody (mAb), participants with a tau-PET signal indicating low-to-medium NFT pathology in their brains showed significantly stronger clinical benefits based on cognitive assessments compared with those whose tau-PET showed more severe NFT pathology before enrollment^26^. This suggests that inhibiting NFT formation could be a viable anti-AD therapeutic approach. A potentially effective strategy to achieve this objective is by targeting STAs. Yet, little is known about the biochemical features of STAs. For example, which tau domains constitute STAs, and which sequences are critical to the aggregation-promoting properties of STAs? What posttranslational modifications characterize these pre-tangle species? Are STAs detectable in human biofluids such as cerebrospinal fluid (CSF) and blood for diagnostic purposes?

Established approaches such as cryo-EM are currently unsuitable for examining STAs given the high solubility, limited defined structure and smaller sizes of STAs compared with fibrils and NFTs^27^. In this study, we used an integrative biochemical approach to identify a defining core sequence of STAs in Tris-buffered saline (TBS) extracts of human AD brain tissue. We show that the STA core region rapidly aggregates in vitro and more robustly alters neuronal excitability than the fibril core identified previously using cryo-EM. Furthermore, we uncover the aggregation-relevant phosphorylation sites p-tau_262_ and p-tau_356_, which are detectable in the pre-NFT stage when commonly used p-tau antibodies such as AT8 (epitope: p-tau_202/205_) show limited reactivity. High-performance liquid chromatography–tandem mass spectrometry (LC–MS/MS) analysis revealed that the STA core region-containing tau forms were near-full-length and had mid-region and microtubule-binding region (MTBR) sequences. Finally, we applied these new insights to develop a biofluid biomarker of pathological tau and verified its clinical performance in an autopsy-verified cohort with antemortem CSF samples and in a cohort with tau-PET imaging.

## Results

### Validation of the biochemical assay for STAs

**Fig. 1:**
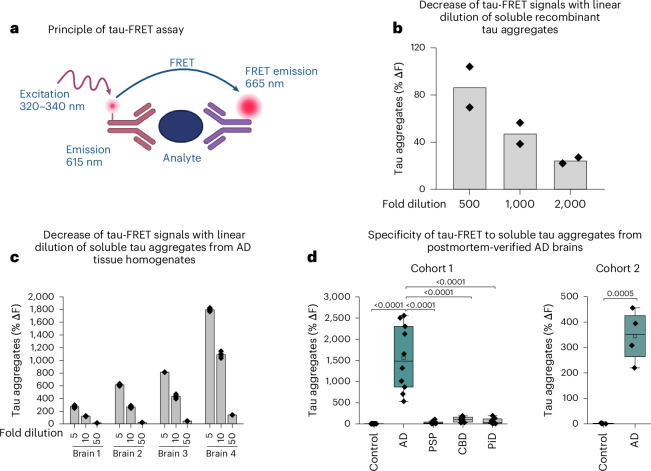
Neuropathological verification of the FRET assay that specifically recognizes STAs from AD but not from other neurodegenerative tauopathies. **a**, Schematic illustration of the principle of the tau-FRET assay. Simultaneous binding of the donor–antibody and acceptor–antibody complexes to a polyvalent analyte leads to excitation followed by energy transfer to a nearby acceptor–antibody that fluoresces at a 665-nm wavelength. **b**, Linearity of decreases in tau-FRET signal in response to dilution of the tau-FRET assay in recombinant tau_441_ diluted 500, 1,000 or 2,000 times. *n* = 2 biological replicates, performed on different days. **c**, Dilution linearity of the tau-FRET assay in brain tissues from four different Braak stage VI individuals with neuropathologically confirmed AD. Data are from three replicates. **d**, Comparison of the tau-FRET signal in neuropathologically diagnosed AD, other neurodegenerative diseases (progressive supranuclear palsy, corticobasal degeneration and PiD) and controls (cohort 1: *n* = 10 participants per group) with a Kruskal–Wallis test with Dunn’s multiple comparisons. Cohort 2 includes neuropathologically diagnosed individuals with AD and controls (*n* = 4 participants each); two-sided Mann–Whitney *U*-test = 0 (*P* = 0.0286). In each panel, data points are shown as the mean ± s.e.m. The boxplot center indicates the median, the box boundaries indicate the 25th and 75th percentiles, and the whiskers indicate extreme values outside the box boundaries (Q1 − 1.5 × IQR and Q3 + 1.5 × IQR), where Q1, Q3 and IQR refer to the 25th percentile, 75th percentile and interquartile range, respectively. To summarize, the FRET assay recognizes soluble and solubilized tau assemblies from recombinant sources and from human brain tissues; it is selective for STAs in AD versus other tauopathies and the signal decrease is proportional to the relative amount of soluble and solubilized assemblies present after sample dilution.

We used a fluorescence resonance energy transfer (FRET) assay to quantify STAs in human autopsied frontal gray matter brain tissues from well-characterized individuals (Fig. 1a). The same anti-tau mAb, targeting a defined sequence in the proline-rich region and initial sequences of the MTBR, was coupled separately to the donor and acceptor molecules^28^. As both antibody conjugates bind to an identical epitope, only one antibody can recognize any given tau monomer. Hence, the donor and receptor conjugates do not reach sufficient proximity to generate a signal. On the other hand, the polyvalency of STAs allows multiple binding events to occur. This leads to excitation of the antibody–donor complex followed by energy transfer to a nearby antibody acceptor that fluoresces at a wavelength of 665 nm. The assay signal (background-subtracted percentage fluorescence signal increase over the negative control) is proportional to the number and sizes of STAs present^28^.

To test assay specificity, we compared equal concentrations of recombinant tau_441_ and α-synuclein as they both aggregate via β-pleated sheet polymerization. Tau_441_ produced higher FRET signals than α-synuclein (Supplementary Fig. 1). Next, we performed dose titrations to evaluate FRET signal equivalence to the STAs present. First, recombinant tau_441_ showed decreasing signals proportional to the dilution fold (Fig. 1b). Then, we examined STAs from AD brain frontal gray matter tissue isolated according to established protocols^29,30^. We used NFT-free TBS-soluble extract of AD brain homogenates (Supplementary Fig. 2). The tau-FRET signal decreased linearly across dilutions (fivefold, tenfold or 50-fold) for four independent cases of identical starting total protein concentration, but with a wide range of starting FRET signals (Fig. 1c).

### FRET assay is specific to AD-type STAs

We hypothesized that like filament heterogeneity^16^, STAs from different tauopathies will have different tau-FRET assay binding. We examined TBS-soluble fractions of frontal gray matter tissue from autopsied cases (cohort 1: *n* = 50; Extended Data Table 1) consisting of AD (*n* = 10) and non-AD tauopathies, including corticobasal degeneration (CBD) (*n* = 10), progressive supranuclear palsy (PSP) (*n* = 10) and Pick’s disease (PiD) (*n* = 10), as well as unaffected controls (*n* = 10). The tau-FRET signal (samples diluted to the same total protein concentration) was strongest in the AD group, being ~300-fold higher than controls (*P* < 0.0001) and 15–43 times higher than the primary tauopathies (*P* = 0.0003 versus PSP and *P* = 0.0048 versus PiD; Fig. 1d). In a validation cohort of autopsy-confirmed AD and control cases without dementia who contributed parietal cortex tissue samples (cohort 2: (ref. ^29^) *n* = 8), the tau-FRET signal was again ~300-fold higher in AD versus control tissues (*P* = 0.0286; Fig. 1d). While the tau-FRET assay had a low coefficient of variation (that is, high precision) in both cohorts, there was remarkable heterogeneity in signals within groups despite all brain samples being at the advanced stages of pathology, suggesting inherent interindividual variability in STA levels. Together, these results show that the FRET assay is selective for AD-type STAs over those in other tauopathies.

### The core region of AD brain STAs

We applied the tau-FRET technique to biochemically characterize STAs in AD brains. As AD tau filaments consist of a core region and an outer layer (fuzzy coat) binding adhesively to the core^16,31,32^, we hypothesized that STAs will have a similar organization. We reasoned that immunodepletion with anti-tau antibodies will first remove the outer fuzzy coat layer before targeting the difficult-to-reach core region. Thus, antibodies targeting epitopes outside the core region will be more efficient at removing the aggregation signal obtained in the FRET assay when that same antibody is used to immunoprecipitate tau from the sample before the FRET analysis, and vice versa. In effect, the core region will be minimally affected by immunodepletion as it should be least accessible to antibodies.

**Fig. 2:**
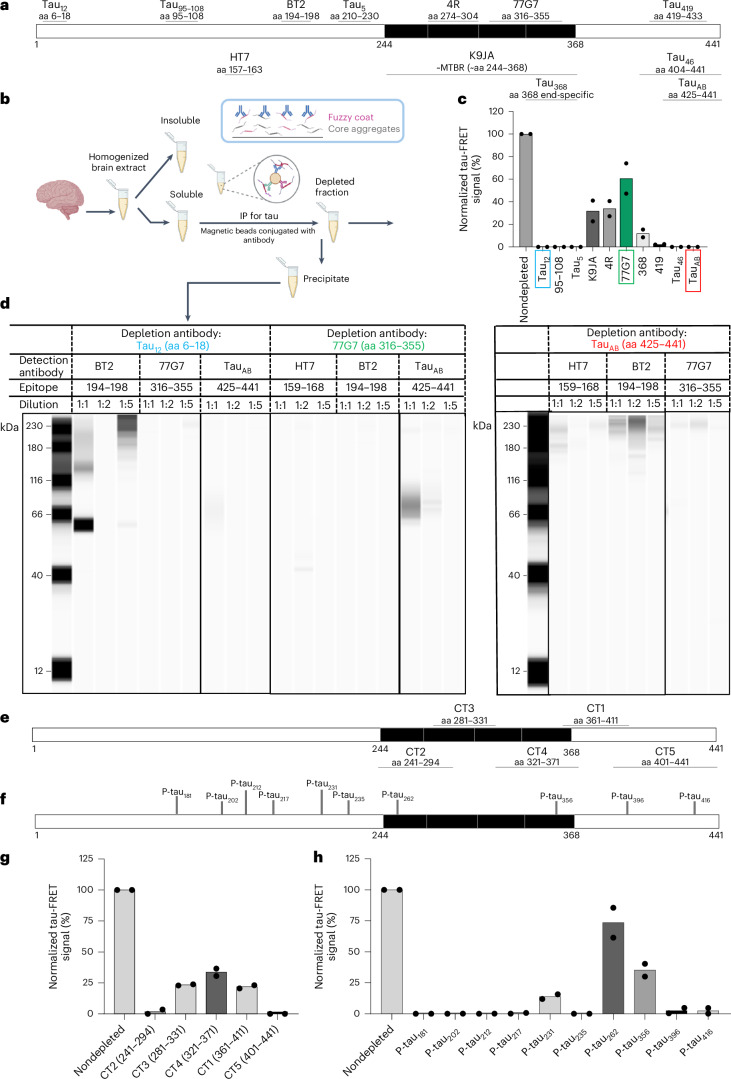
Integrative biochemical analyses identify a putative core sequence of AD-type STAs in human brain tissue. **a**, Schematic illustration of the epitopes of the anti-tau antibodies used in the IP experiments. These antibodies target defined nonphosphorylated tau sites; their sources are described in Supplementary Information. **b**, Flow diagram of the experimental procedure used to isolate TBS-soluble tau fractions from AD human brains. Aliquots of the soluble fraction underwent immunodepletion using a defined antibody, including those shown in **a**. Afterward, the depleted fraction was examined with the FRET assay described in Fig. 1, while the precipitate portion was evaluated using immunoblotting. The diagram in the inset illustrates that fuzzy coat peptides are more accessible to antibodies and thus are first removed in the imunodepletion step using IP with those antibodies. **c**, tau-FRET signals obtained before (‘nondepleted’) and after (denoted by the name of the depleting antibody) immunodepleting tau content from the TBS-soluble brain tissue fraction from a patient with autopsy-verified Braak stage VI AD using the named antibodies covering the tau_441_ sequence. Data are shown as the mean with individual data points overlaid. *n* = 2 biological replicates. **d**, For three antibodies with epitopes in the N terminus (tau_12_), MTBR (77G7) and the extreme C terminus (tau_AB_), the corresponding immunoreactivity of the precipitate fractions to the selected antibodies using immunoblotting is shown. Representative images from three biological replicates. **e**, Schematic illustration of the epitopes of the set of ‘CT’ mAbs developed in this study against defined regions inside and outside the MTBR. **f**, Schematic illustration of the anti-tau antibodies used in this study that target the phosphorylation sites. **g**, tau-FRET assay signals after immunodepletion of TBS-soluble brain fractions with the new CT mAbs relative to a nondepleted control. Data are shown as the mean with individual data points overlaid. *n* = 2 biological replicates. **h**, tau-FRET assay signals after immunodepletion of TBS-soluble brain fractions with anti-tau antibodies that target defined phosphorylation epitopes relative to a nondepleted control. Data are shown as the mean with individual data points overlaid. *n* = 2 biological replicates. This figure, together with Extended Data Figs. 1–3, shows that STAs in TBS-soluble AD brain tissue contain a core region that covers the peptide ~tau_258–368_. IP followed by high-resolution MS analysis using several anti-tau antibodies identified that tau forms that contain this STA core region are long, near-full-length fragments that stretch from the N terminus or mid-region into the MTBR.

We evaluated approximately two dozen antibodies targeting tau epitopes spanning the 441-amino acid (aa) full-length tau sequence (Fig. 2a). Depleting TBS-soluble AD brain tissue homogenates with the N-terminus-targeting antibodies tau_12_ and tau_95–108_ (epitopes: aa 6–18 and aa 95–108, respectively) as well as the mid-region antibody tau_5_ (aa 210–230), each led to complete removal of the tau-FRET signal (Fig. 2b,c), which taken together suggests the presence of long, near-full-length tau species. Similar results were obtained with the C-terminal antibodies tau_46_ (aa 404–441), tau_AB_ (aa 425–441) and tau_419_ (aa 419–433) (Fig. 2c). However, depletion with the MTBR-targeting polyclonal antibody K9JA led to a 70.4% decrease in signal (Fig. 2c). When using mAbs against defined MTBR epitopes, four-repeat-tau (~aa 275–291) removed a similar amount of the FRET signal (67.3%) while 77G7 (aa 315–355) removed less (42.7%) (Fig. 2c). However, tau_368_ (neospecific for truncation at aa 368 (ref. ^33^)) targeting the end of the MTBR removed much more FRET signal (88.9%). These findings could be caused by a decrease in the accessibility of the antibodies to the MTBR through which tau aggregates. The results further suggest that the STA core is located mostly in the R2–R4 region, as application of antibodies targeting this region produced the smallest depletion effect, which corresponds to the weakest attenuation of the tau-FRET signal.

To probe this further, we generated a set of antibodies covering the entire MTBR and the C terminus (Fig. 2e). The least depletion was obtained with CT4 (aa 321–371; 66.8% of the signal removed) followed by CT3 (aa 281–331; 76.6% removed) and CT1 (aa 361–411; 78.3% removed) (Fig. 2g). In agreement with Fig. 2c, CT2 (aa 241–294; targeting mostly the R1 region) and CT5 (aa 401–441; directed at the extreme C terminus) were the most efficient at signal depletion (98–100% of the signal removed; Fig. 2g). These results further indicate that the STA core may be located principally in the R2–R4 region.

Next, we examined phosphorylation (p-tau) sites in the STA core (Fig. 2f). Immunodepleting with p-tau_262_ and p-tau_356_ (serine residues in the KXGS motifs in the R1 and R4 repeats, respectively) showed limited antibody accessibility, suggesting that these epitopes are integral to the core (Fig. 2h). Corroborating the results in Fig. 2c,g, immunodepleting with p-tau antibodies in the N terminus to mid-region (p-tau_181_, p-tau_202_, p-tau_212_, p-tau_217_, p-tau_231_ and p-tau_235_) or C terminus (p-tau_396_ and p-tau_416_) removed ~100% of the tau-FRET signal (Fig. 2h). Together, given that the STA core is prominently phosphorylated at serine-262 and contains the R2 region (four-repeat-tau), we conclude that it starts from approximately aa 258 and ends at aa 368 where there is a major NFT-promoting pathological truncation^33^.

### The fuzzy coat contains N-terminal and C-terminal tau

To further verify the STA core and the molecular forms of tau that make up the fuzzy coat, we used immunoblotting capillary electrophoresis^34^ to probe the antigen–antibody–protein G-precipitated fractions from the immunodepletion experiments in Fig. 2b,c. Antibodies that efficiently depleted STAs (thus leading to high decreases in tau-FRET signals) had these signals consolidated in the precipitate, resulting in high immunoblotting immunoreactivity, and vice versa (Fig. 2b–d). Moreover, we hypothesized that antibodies that efficiently deplete most of the signal will cover the nonaggregating fuzzy coat region because the STA core should be least accessible to antibodies (Fig. 2b). Tau_12_ was efficient at depleting the tau-FRET signal (Fig. 2c). To estimate the length of tau_12_^+^ fragments, we screened antibodies covering neighboring sites. Tau_12_-precipitated tau stained positive for BT2 (aa 194–198) but not 77G7 or tau_AB_, meaning that tau_12_-containing forms stretched into the mid-region but not into the far end recognized by 77G7 or the extreme C terminus where tau_AB_ binds (Fig. 2d). In contrast, 77G7 was poor at depleting the tau-FRET signal (Fig. 2c), suggesting that it targets an epitope within the STA core. 77G7-precipitated tau fractions were negative for HT7 (aa 159–163) and BT2, indicating that the tau fragment immunoprecipitated by the 77G7 antibody lacked the mid-region sequences recognized by HT7 and BT2 (Fig. 2d). When considering the C terminus, the tau_AB_-precipitated fraction did not stain strongly for the mid-region-targeting antibodies HT7 and BT2 or the MTBR-binding 77G7 (Fig. 2d). Full-length blots are shown in Supplementary Fig. 3.

### Molecular identity of tau forms that contain the STA core

**Extended Data Fig. 1:**
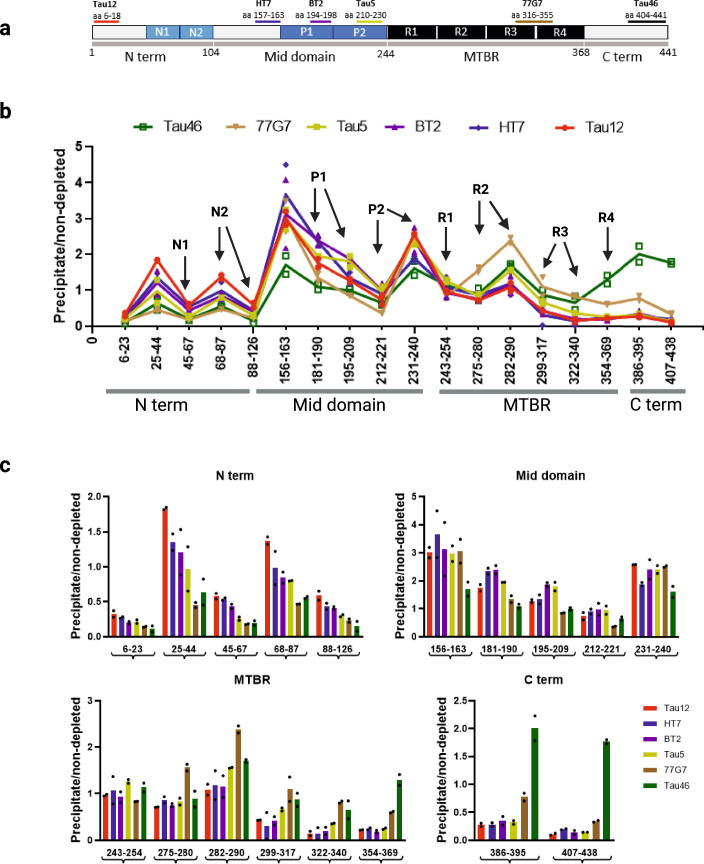
Mass spectrometric characterization of the molecular identity of tau forms that contain the core region of soluble tau assemblies (STAs). (**a**) Schematic illustration of the structure of the longest tau isoform (2N4R) and epitopes of tau antibodies used for immunoprecipitation. (**b**) Line graphs for the normalized ratio of the peptide peak area in the precipitate over non-depleted fractions across immunoprecipitations (IPs) with various tau antibodies. The peak area of the tau peptides was based on PRM assays on Exploris™ 480. The ratio of each IP was normalized to a mean of 1. N = 2 biological replicates. (**c**) Bar graph representations for the normalized ratio shown in (**b**). The error bars depict the standard error of the mean. N = 2 biological replicates.

We performed paramagnetic bead-based immunoprecipitation (IP) of a pooled TBS-soluble AD brain homogenate using six antibodies together stretching the tau_441_ sequence (Extended Data Fig. 1a), comparing tau forms in the nondepleted (before IP), depleted (supernatant after IP) and precipitate (immunoprecipitated; tau forms pulled by the antibody of interest and thus enriched on the beads) fractions by MS using a parallel reaction monitoring (PRM) assay on a high-resolution quadrupole Orbitrap hybrid instrument (Orbitrap Exploris 480) online coupled with nanoflow LC. We monitored the levels of 18 tau peptides spanning aa 6–438 of tau 2N4R (Extended Data Fig. 1a) and quantified peptide intensity using the sum intensity area of the top three quality fragments of each PRM assay (Supplementary Table 1). Generally, the intensity area for the precipitate fraction was much stronger than the same for the nondepleted and depleted fractions, demonstrating efficient IP (Supplementary Figs. 4–9). No tau peptide was detected in the precipitate when a mouse IgG isotype control was used for the IP (Supplementary Fig. 10).

We compared the enrichment efficiency of tau peptides across several regions of the protein by assessing the normalized precipitate to nondepleted ratios, with the mean ratio set to 1 to standardize IP with different antibodies (Extended Data Fig. 1b,c). We observed that IP with the N-terminus-targeting antibody tau_12_ strongly enriched the N-terminal and mid-region tau forms in the precipitate, except for isoform-specific sequences in the 1N and 2N regions (aa 45–103) for which results were driven by isoform abundance. However, there was a sharp decrease in peptides enriched in the precipitate after aa 209 but not for the second proline-rich region and the MTBR sequences aa 231–240 and aa 282–290, respectively, which remained abundant in the precipitate fraction (Extended Data Fig. 1 and Supplementary Fig. 4). These results agree with the independent findings in Fig. 2 that did not include MS data. Together, the results indicate that tau_12_-containing tau forms can stretch not only from the N terminus to the mid-region but also into the N-terminal parts of the MTBR. This explains why tau_12_-precipitated tau forms stained positive for the mid-region-targeting antibody BT2 (epitope: aa 194–198) but not 77G7 (epitope: aa 316–355), which recognizes sequences toward the C terminus of the MTBR (Fig. 2d). Moreover, it clarifies the efficiency of tau_12_ (and by extension the antibody 95–108) to deplete the tau-FRET signal (Fig. 2c) as they bind tau forms covering the region which the antibody used in the FRET assay recognizes.

The mid-region-directed antibodies HT7, BT2 and tau_5_ were not as efficient as tau_12_ in precipitating N-terminal tau, but they were highly efficient in enriching mid-region tau and MTBR sequences, including aa 231–240 and 282–290, but not any peptide C terminus to these (Extended Data Fig. 1 and Supplementary Figs. 5–7). Compared to tau_12_, 77G7 showed reduced efficiency in precipitating the N terminus and mid-region of tau but much higher efficiency for the MTBR and C terminus, particularly after aa 275 (the R2 MTBR region) (Extended Data Fig. 1 and Supplementary Fig. 8). Finally, the tau_46_ antibody precipitated fragments containing the MTBR-end species through the C terminus (that is, aa 354–438), and some of the proline-rich region and N-terminal MTBR sequences, including aa 231–240 and 282–290, and the mid-region peptides 156–163 (Extended Data Fig. 1 and Supplementary Fig. 9). Together, tau forms that contain the STA core included near-full-length sequences that can stretch from the N terminus to the MTBR and from the mid-region to the far C terminus.

Furthermore, immunoblotting showed that tau_12_ prominently stained the nondepleted brain tissue sample. For the tau_12_ immunoprecipitated samples, the signal was much stronger in the precipitate versus depleted fractions when probed with tau_12_ (Supplementary Fig. 11). This explains the efficiency of tau_12_ at immunoprecipitating STAs in the FRET (Fig. 2) and MS (Extended Data Fig. 1) experiments. Together, the immunoblotting findings, further corroborating the results from the FRET and MS experiments, highlight the diversity of tau forms that contain the STA core region.

### P-tau_262_ and p-tau_356_ in the STA core detect early-stage NFTs

We performed immunohistochemical analyses of autopsy-verified cases with either low (Braak NFT stages 0–II) or high (Braak NFT stages V and VI) tau pathology, focusing on the CA1 region of the hippocampus, which is highly vulnerable to AD tau pathology^35^. We immunostained for p-tau_262_ and p-tau_356_, within the STA core, as well as p-tau_231_ (AT180) and AT8 (p-tau_202/205_), outside of the core region.

**Fig. 3:**
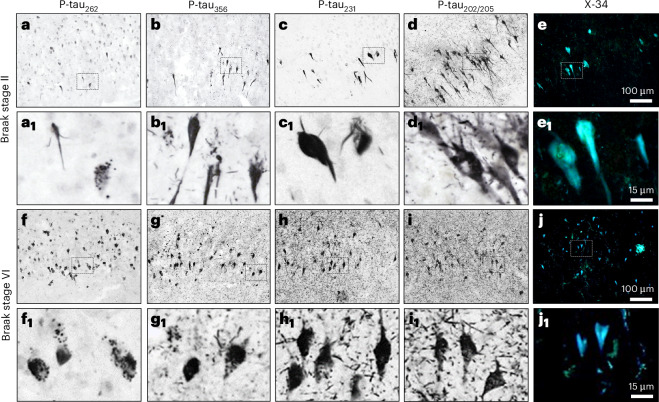
Chromogen immunohistochemistry (IHC) analyses of tau forms with phosphorylated epitopes inside (p-tau_262_, p-tau_356_) and outside (p-tau_231_, p-tau_202/205_ (AT8)) the STA core region in postmortem human hippocampus. **a**–**j**, Photomicrographs of hippocampal tissue sections from an individual with Braak NFT stage II (**a**–**e**,**a**_**1**_–**e**_**1**_) and an individual with Braak NFT stage VI (**f**–**j**,**f**_**1**_–**j**_**1**_) immunohistochemically processed using antibodies directed against the p-tau_262_ epitope of the tau protein (**a**,**a**_**1**_,**f**,**f**_**1**_), the p-tau_356_ epitope (**b**,**b**_**1**_,**g**,**g**_**1**_), the p-tau_231_ epitope (clone AT180; **c**,**c**_**1**_,**h**,**h**_**1**_) and the p-tau_202/205_ epitope (clone AT8; **d**,**d**_**1**_,**i**,**i**_**1**_). The pan-amyloid binding dye X-34 was used to confirm the presence of fibrillar tau (**e**,**e**_**1**_,**j**,**j**_**1**_). Immunolabeling and histofluorescence observed in the CA1 region, near the CA1/CA2 border, are illustrated at low (**a**–**e**,**f**–**j**) and higher (**a**_**1**_–**e**_**1**_,**f**_**1**_–**j**_**1**_) magnification. The locations of the higher-magnification images are indicated by dashed outlines in the low-magnification images. The illustrated immunohistochemical staining was replicated in three sections per individual in each of three individuals with Braak II and five individuals with Braak VI. In summary, the p-tau_262_ and p-tau_356_ immunostaining is localized mainly to granular structures inside hippocampal neurons, whereas the p-tau_231_ and p-tau_202/205_ antibodies show a more diffuse pattern of immunolabeling in hippocampal neurons, and in neuropil threads, at both Braak II and VI NFT stages.

At the low Braak NFT stages (for example, Braak stage II; Fig. 3), each antibody labeled isolated groups of CA1 pyramidal cells (Fig. 3a–d,a_1_–d_1_), whereas at the high Braak NFT stages (for example, Braak stage VI; Fig. 3) they revealed high frequencies of immunolabeled cells throughout the CA1 region (Fig. 3f–i,f_1_–i_1_). The presence of fibrillar tau was confirmed using the pan-amyloid binding dye X-34 (Fig. 3e,e_1_,j,j_1_). The p-tau_262_ and p-tau_356_ immunostaining patterns differed from p-tau_231_ and p-tau_202/205_ antibodies in the overall appearance and extent of labeling neurons and neuritic pathology (dendritic tau pathology presenting as neuropil threads and axonal tau pathology presenting as dystrophic neurites in neuritic plaques). The p-tau_262_-directed antibody labeled pre-NFTs with a predominantly granular, vesicle-like immunolabeling pattern in portions of the cell soma and, less frequently, a diffuse, confluent immunolabeling over the cell soma and proximal dendrites at both low and high Braak NFT stages (Fig. 3a_1_,f_1_), while p-tau_262_ labeling of neuropil threads was rare or absent (Fig. 3a,a_1_,f,f_1_). The p-tau_356_-directed antibody resulted in more instances of confluent staining in pyramidal cell bodies and portions of proximal dendritic processes, as well as granular and vesicle-like staining, in both low and high Braak NFT stages (Fig. 3b,b_1_,g,g_1_). In cases at the high Braak NFT stages, the p-tau_356_ antibody also labeled small numbers of neuropil threads and neuritic processes (Fig. 3g,g_1_). Compared with the p-tau_262_ and p-tau_356_ immunostains, the p-tau_231_-directed (Fig. 3c,c_1_,h,h_1_) and p-tau_202/205_-directed (Fig. 3d,d_1_,i,i_1_) antibodies yielded the most robust staining, primarily confluent, of pre-NFTs and mature NFTs in the low and high Braak NFT cases; in the latter, they also revealed a dense network of neuropil threads (Fig. 3h,h_1_,i,i_1_) and dystrophic neurites (for example, p-tau_231_-labeled neuritic plaque is present in the middle bottom of Fig. 3h).

**Fig. 4:**
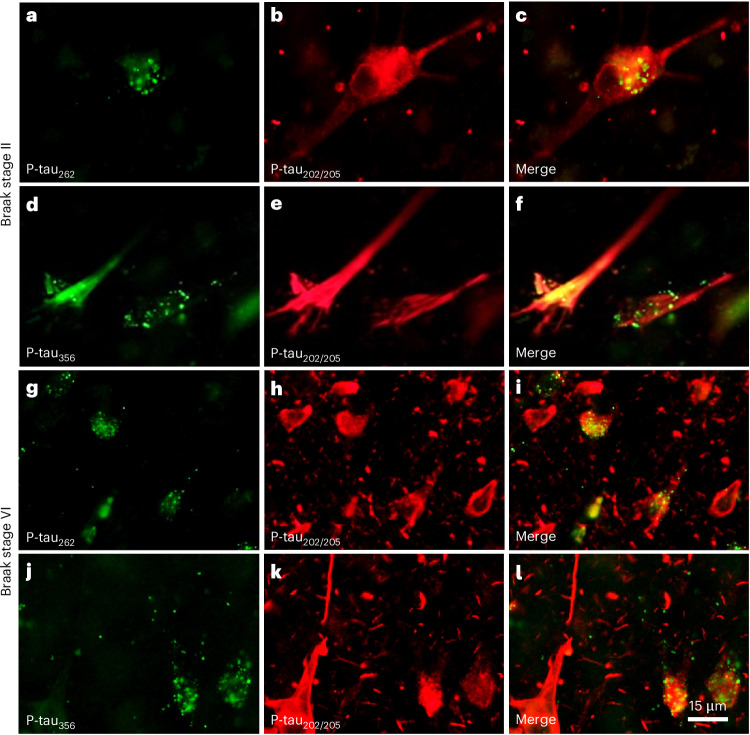
Dual immunofluorescence staining of the p-tau_262_ and p-tau_356_ sites in the STA core versus p-tau_202/205_ (AT8) in the fuzzy coat in human postmortem tissue at early and late Braak NFT stages. **a**–**l**, Tissue sections of the hippocampus from an individual at Braak NFT stage II (**a**–**f**) and an individual at Braak NFT stage VI (**g**–**l**) were processed using dual immunofluorescence to assess the codistribution of p-tau_262_ labeling with p-tau_202/205_ (AT8) labeling (Braak II: **a**–**c**; Braak VI: **g**–**i**) and p-tau_356_ labeling with p-tau_202/205_ (AT8) labeling (Braak II: **d**–**f**; Braak VI: **j–l**). In each triplet, green fluorescence indicates p-tau_262_ (**a**,**g**) or p-tau_356_ (**d**,**j**); red fluorescence indicates p-tau_202/205_ (AT8) (**b**,**e**,**h**,**k**). Merged images are shown in **c**,**f**,**i**,**l**. The illustrated immunohistochemical staining was replicated in three sections per individual in each of three Braak II and five Braak VI individuals. Together, in Braak NFT stage II, hippocampal neurons with confluent p-tau_202/205_ labeling also contained p-tau_262_ or p-tau_356_-labeled granular structures in a portion of the cell cytoplasm, whereas in Braak NFT stage VI, only a subset of hippocampal neurons with confluent p-tau_202/205_ labeling also contained p-tau_262_ or p-tau_356_ immunofluorescence.

Dual immunofluorescence was used to assess the relationship of p-tau_262_ and p-tau_356_ with p-tau_202/205_ immunolabeling in CA1 pyramidal neurons from cases with low and high Braak NFTs. In low Braak NFTs, many neurons with confluent p-tau_202/205_ labeling throughout their soma and processes had p-tau_262_ or p-tau_356_ labeling restricted to only a portion of the cell cytoplasm, mostly in clusters of vesicle-like granular structures (Fig. 4a–f). In high Braak NFTs, p-tau_262_ and p-tau_356_ immunofluorescence was observed in only a subset of p-tau_202/205_-labeled CA1 pyramidal neurons and was almost completely absent from p-tau_202/205_-labeled neuropil threads (Fig. 4g–l). In instances of intracellular codistribution, p-tau_262_ or p-tau_356_ immunofluorescence signals were localized to a portion of the cell soma in contrast to the confluent p-tau_202/205_ signal that extended throughout the cell body.

**Extended Data Fig. 2:**
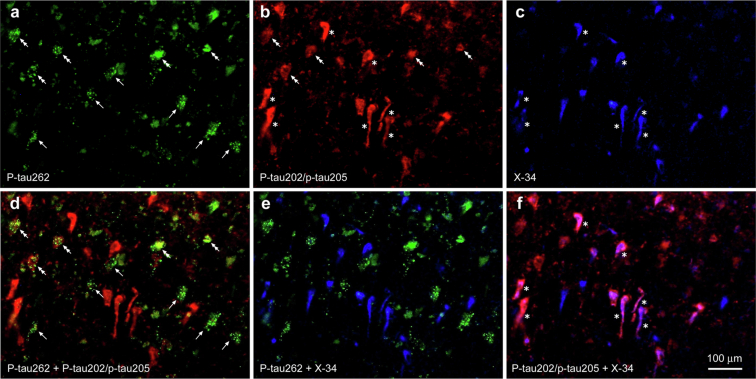
Multifluorescence analyses of co-distribution of a tau form phosphorylated at an epitope within the STA core region (p-tau262) with a tau form phosphorylated at an epitope outside the STA core region (p-tau202/p-tau205[AT8]) and with the pan-amyloid binding dye X-34. Fluorescence photomicrographs of a single hippocampal tissue section from a case with Braak NFT stage V processed for p-tau262 (**a**, green), p-tau202/p-tau205 (**b**, red), and X-34 stain (**c**, blue). **d**-**f**: co-distribution patterns of p-tau262 and p-tau202/p-tau205 (**d**), p-tau262 and X-34 (**e**), and p-tau202/p-tau205 and X-34 (**f**). Cells with granular p-tau262 immunofluorescence in their soma show either weak p-tau202/p-tau205 signal (**a**, **b**, **d**; double arrows) or no p-tau202/p-tau205 signal (**a**, **d**; single arrows). Most cells labelled with the X-34 stain also contain p-tau202/p-tau205 immunofluorescence, particularly classic neurofibrillary tangles (**b**, **c**, **f**, Asterisk). In contrast, no overlap is observed between p-tau262 and X-34 signals (**e**). Scale bar: 100 µm. The illustrated immunofluorescence staining was replicated in three sections per case in each of three Braak V cases. Together, in the hippocampus, neurofibrillary tangles with X-34 labelled fibrils frequently co-localize with p-tau202/p-tau205 immunosignal but do not have appreciable immunostaining by the p-tau262 antibody.

**Extended Data Fig. 3:**
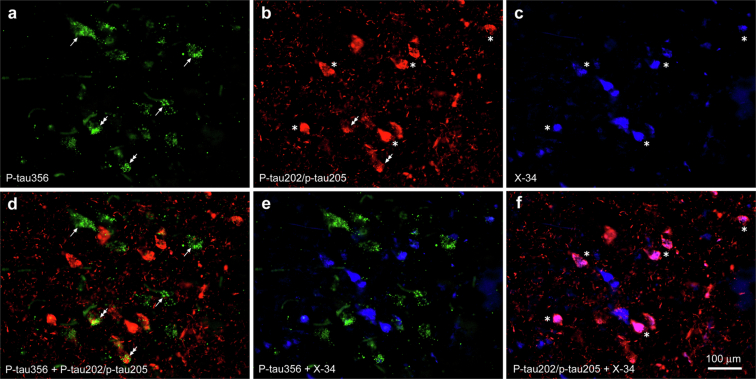
Multifluorescence analyses of co-distribution of a tau form phosphorylated at an epitope within both the STA core region and fibril core (p-tau356) with a tau form phosphorylated at an epitope outside the either core region (p-tau202/p-tau205) and with the pan-amyloid binding dye X-34. (**a**-**c**). Fluorescence photomicrographs of hippocampal tissue sections from a case with Braak NFT stage V illustrating p-tau356 (**a**, green) and p-tau202/p-tau205 (**b**, red) fluorescence immunolabeling and X-34 stain (**c**, blue). d-f: co-distribution patterns p-tau356 and p-tau202/p-tau205 (**d**), p-tau356 and X-34 (**e**), and p-tau202/p-tau205 and X-34 (**f**). Cells with granular p-tau356 immunofluorescence in their soma show either weak p-tau202/p-tau205 signal (**a**, **b**, **d**; double arrows) or no p-tau202/p-tau205 signal (**a**, **d**; single arrows). Most cells labelled with the X-34 stain also contain p-tau202/p-tau205 immunofluorescence, particularly classic neurofibrillary tangles (**b**, **c**, **f**, asterisk). In contrast, no overlap is observed between p-tau356 and X-34 signals (**e**). Scale bar: 100 µm. The illustrated immunofluorescence staining was replicated in three sections per case in each of three Braak V cases. To summarize, in the hippocampus, neurofibrillary tangles with X-34 labelled fibrils frequently co-localize with p-tau202/p-tau205 immunosignal but do not have appreciable immunostaining with p-tau356 antibody.

To determine the relationship of the p-tau_262_ and p-tau_356_ immunosignal with p-tau_202/205_ labeling in pre-NFTs that lack tau fibrils and in NFTs that contain fibrillar tau, we performed triple fluorescence labeling experiments combining p-tau_262_ or p-tau_356_ with p-tau_202/205_ and the pan-amyloid binding dye X-34, which labels tau fibrils in NFT and Aβ fibrils in plaques and cerebral amyloid angiopathy. These experiments demonstrated that X-34-labeled NFTs frequently contained the p-tau_202/205_ signal but did not have appreciable p-tau_262_ (Extended Data Fig. 2) or p-tau_356_ immunosignal (Extended Data Fig. 3).

### Specificity of the p-tau_262_ and p-tau_356_ antibodies

**Extended Data Fig. 4:**
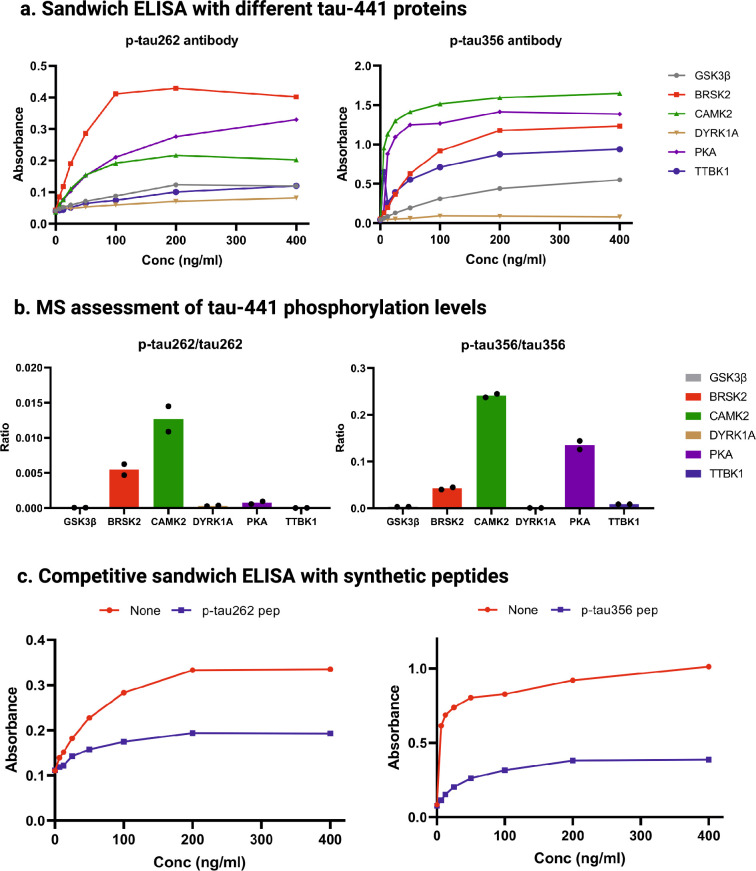
Biochemical characterization of the specificity of the p-tau262 and p-tau356 antibodies. (**a**) Sandwich ELISA results utilizing either the p-tau262 (left) or p-tau356 (right) antibodies for capture and biotinylated anti-tau mouse mAb, clone Tau12 as the detector. Recombinant tau441 proteins, phosphorylated by different kinases, were assayed at 0 to 400 ng/ml concentrations. N = 2 biological replicates. (**b**) Mass spectrometric characterization of the phosphorylation levels at S262 (left) and S356 (right) of various recombinant tau441 proteins as a ratio against the non-phosphorylated version of the same peptide. The quantification was based on PRM-measured peak areas of peptides: IGpSTENLK (p-tau262), IGSTENLK (tau262), IGpSLDNITHVPGGGNK (p-tau356), and IGSLDNITHVPGGGNK (tau356), with p indicating phosphorylation. N = 2 biological replicates. (**c**) Competitive ELISA results for p-tau262 (left) and p-tau356 (right) antibodies. The assays tested varying concentrations of CAMK2-phosphorylated tau441 protein against a set concentration (0.1 μg/ml) of the indicated synthetic peptides phosphorylated at Ser262 or Ser356 (p-tau262 pep or p-tau356 pep; blue) or not (None; red). These peptides, corresponding to Tau441 amino acids 257 to 267 (KSKIGSTENLK) for p-tau262 and Tau441 amino acids 351 to 361 for p-tau356, were linked to an N-terminal cysteine via a peptide bond and, where indicated, phosphorylated at the specified residues. N = 2 biological replicates.

Previous studies reported tau phosphorylation by Ca^2^^+^ and calmodulin-dependent protein kinase II (CAMK2) at serine-262 (refs. ^36,37^) and at serine-262 and serine-356 jointly^38^, as well as by BR serine/threonine kinase 2 (BRSK2) at serine-262 (refs. ^39,40^). Protein kinase A was reportedly involved in tau phosphorylation at serine-262 (ref. ^37^). We performed sandwich enzyme-linked immunosorbent assays (ELISAs) with six different recombinant tau_441_ proteins in vitro phosphorylated by these kinases, which were commercially sourced from the same vendor. In sandwich ELISA, the proteins with the highest reactivity to both the p-tau_262_ and p-tau_356_ antibodies were those phosphorylated by CAMK2, BRSK2 and protein kinase A (Extended Data Fig. 4a). To assess if the varying levels of reactivity aligned with phosphorylation levels, we used MS to compare the ratio of the phosphorylated and nonphosphorylated versions of tryptic peptides covering the serine-262 and serine-356 epitopes across different recombinant tau_441_ proteins (Supplementary Table 2). The MS data largely mirrored the ELISA findings, with CAMK2-phosphorylated and BRSK2-phosphorylated tau_441_ having high p-tau_262_/tau_262_ and p-tau_356_/tau_356_ ratios (Extended Data Fig. 4b).

To further confirm antibody specificity, we performed competitive sandwich ELISAs using the highly reactive CAMK2-phosphorylated tau_441_ protein, which reacted dose-dependently when titrated against the p-tau_262_ or p-tau_356_ antibody. The signals were substantially decreased when synthetic peptides phosphorylated specifically at serine-262 or serine-356 were added in the reaction mixture to compete with the antibodies for target engagement (Extended Data Fig. 4c). These results show that the p-tau_262_ and p-tau_356_ antibodies are specific to tau forms phosphorylated at serine-262 or serine-356.

### In vitro aggregation of the STA core peptide

**Fig. 5:**
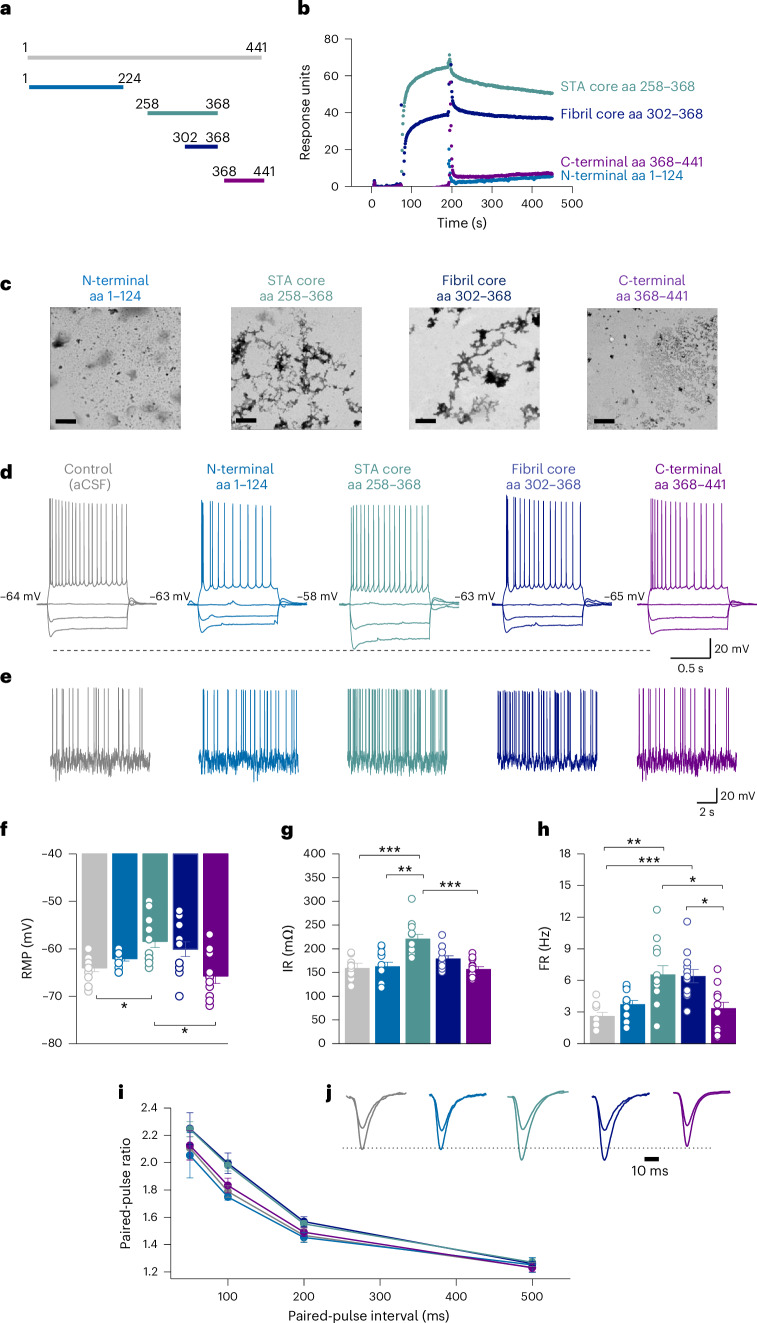
The core region peptide of STAs potently modulates neuronal function. **a**, Schematic representation of recombinantly produced truncated tau species covering the putative STA core sequence (aa 258–368), the insoluble fibril core peptide (aa 302–368) and the flanking N-terminal (aa 1–124) and C-terminal (aa 368–441) peptide controls. **b**, SPR profiles of the recombinant STA core peptide relative to the fibril core sequence, and the N-terminal and C-terminal peptides. The SPR sensorgrams illustrate the effectiveness of the CT19.1 antibody in recognizing the specific epitope within the tau sequence (aa 331–361). The N-terminal tau fragment (aa 1–224) and the C-terminal tau fragment (aa 368–441) fall outside the epitope region. Consequently, their respective sensorgrams closely resemble the baseline signal. In contrast, the sensorgrams for the soluble (aa 258–368) and insoluble (aa 302–368) core fragments exhibit higher signals. The SPR plots were generated from *n* = 3 replicates. **c**, Representative negative stain transmission electron microscopy (TEM) images of the recombinant STA core, fibril core, and the N-terminal and C-terminal control peptides. Images are representative of *n* = 2 replicates. Scale bars, 500 nm. **d**, Whole-cell patch clamp recordings were made from CA1 pyramidal neurons in acute mouse hippocampal brain slices. Representative examples of standard current-voltage responses for slices incubated in soluble aliquots of the recombinant tau peptides (N terminus, *n* = 12; STA core, *n* = 12; fibril core, *n* = 12; or C terminus, *n* = 12) or control aCSF (*n* = 12) for 1 h before electrophysiological recordings. Incubation with the STA core region peptide depolarized the RMP and increased the IR of the recorded neurons. Each ‘*n*’ in **d**–**h** represents a whole-cell patch clamp recording made from individual acute hippocampal brain slices incubated with one of the recombinant tau peptides (diluted in aCSF; Methods) or control aCSF. **e**, Representative example of membrane potential responses to naturalistic current injection^86^ for each of the three conditions described in **d**. **f**, Incubation with the STA core peptide resulted in a significant depolarization of the RMP (mean RMP in slices incubated with the STA core of −58 ± 1.31 mV^−1^ (*n* = 12) compared with −65 ± 0.83 mV^−1^ in controls (*n* = 12)); Kruskal–Wallis test (Kruskal–Wallis statistic = 14.27, *P* = 0.0065) with Dunn’s multiple comparisons (*P* = 0.0400), which was not observed with the other tau truncations. Data are presented as the mean ± s.e.m., with individual data points overlaid. RMP depolarization with the STA core also significantly differed from that of the C-terminal peptide. **g**, Incubation with the STA core peptide also significantly increased IR (mean IR in slices incubated with the STA core peptide was 220.7 ± 9.6 mΩ (*n* = 12), compared with the mean IR in aCSF control slices of 159.3 ± 5.6 mΩ (*n* = 12); Kruskal–Wallis test (Kruskal–Wallis statistic = 24.60, *P* < 0.0001) with Dunn’s multiple comparisons (*P* = 0.0008)), an effect which was also not observed with the other tau truncations. Data are presented as the mean ± s.e.m., with individual data points overlaid. **h**, Incubation with the STA core peptide significantly increased the FR (a correlate of neuronal excitability). The mean FR in slices incubated with the STA core was 6.4 ± 0.85 Hz (*n* = 12), compared to the mean FR in aCSF control slices of 2.7 ± 0.32 Hz (*n* = 12); Kruskal–Wallis test (Kruskal–Wallis statistic = 25.66, *P* < 0.0001) with Dunn’s multiple comparisons (*P* = 0.0016). Incubation with the fibril core also significantly increased the FR compared to aCSF controls (mean FR in slices incubated with the fibril core was increased to 6.3 ± 0.62 Hz (*n* = 12); Kruskal–Wallis test with Dunn’s multiple comparisons (*P* = 0.0006). No change versus aCSF control was observed with N-terminal or C-terminal tau. Data are presented as the mean ± s.e.m., with individual data points overlaid. **i**, Graph showing the mean paired-pulse ratio against interval for the STA core peptide versus the other tau truncations. Incubation with the STA core peptide or the fibril core peptide significantly enhanced paired-pulse facilitation compared with aCSF control slices. The mean paired-pulse ratio at a 100-ms interval was 1.78 ± 0.12 in control aCSF (*n* = 9 slices) compared with the STA core (2.01 ± 0.09; *P* = 0.0190; *n* = 9 slices) and 2.03 ± 0.17 for the fibril core (*n* = 8 slices). There was a significant difference between these values; Kruskal–Wallis test (Kruskal–Wallis statistic = 21.05, *P* = 0.0003) with Dunn’s multiple comparisons (*P* = 0.0359). **j**, Inset to **i**: representative example traces of fEPSP waveforms for the 100-ms interval for each tau peptide. The first fEPSPs were normalized so that facilitation could be compared across conditions. *P ≤ 0.05, **P ≤ 0.01, ***P ≤ 0.001. *P* values for between-group comparisons have been provided in Supplementary Table 3. To summarize, the results demonstrate that the recombinant STA core region peptide has a strong in vitro aggregation propensity and robust impairment of neuronal excitability and functional modulation of neuronal and network functions in mouse hippocampal slices.

To investigate the functional effects of the STA core peptide in vitro, we expressed and purified its recombinant form (aa 258–368) and compared its characteristics with those of the fibril core previously identified using cryo-EM^17^ (~aa 302–368) and N-terminal and C-terminal controls (aa 1–224 and 368–441, respectively; Fig. 5a).

In the surface plasmon resonance (SPR) experiments, an anti-tau antibody (CT19.1; aa 331–361) was immobilized onto a CM5 chip surface and peptide binding responses were recorded in cycles of binding and regeneration. The STA and fibril core peptides gave the highest SPR signals while the N-terminal and C-terminal sequences had no appreciable SPR increases from baseline (Fig. 5b). As the SPR signal is equivalent to the mass and number of molecules of the analyte (that is, the aggregates formed in this case) bound to the surface^41^, these results suggest that the STA core peptide aggregates faster than the fibril core; however, the fuzzy coat structures do not form aggregates. In agreement, electron microscopy analysis showed that the STA and fibril cores formed aggregates, with structures from the latter appearing larger (Fig. 5c). As expected, the N-terminal and C-terminal control sequences did not show aggregate formation (Fig. 5c).

### The STA core peptide alters neuronal excitability

We previously used an in vitro electrophysiology approach to quantify the functional effects of well-characterized recombinant tau species on neuronal and network function^24,25,42^. This approach enables control over the structure and concentration of tau forms and permits studies of different tau forms, including truncated peptides as well as monomers and oligomeric assemblies^24,42^. In this study, acute mouse hippocampal slices were incubated in identical concentrations of recombinant tau peptides covering the STA core region identified in this study (the ~aa 258–368 STA core), the ~aa 302–368 AD fibril core identified previously by cryo-EM, N terminus (aa 1–224) and C terminus (368–441) control peptides contained in the fuzzy coat peptides or in control buffer (artificial CSF (aCSF)) (Fig. 5a) for 1 h before electrophysiological recording. Whole-cell patch clamp recordings were made from mouse hippocampal CA1 pyramidal cells. Neuronal function was measured using stepwise and dynamic current injection protocols (Fig. 5d,e). Incubation with the STA core peptide led to significant depolarization (7 mV) of the resting membrane potential (RMP) versus aCSF control slices (*P* = 0.0400; Fig. 5f) and significant increases in input resistance (IR) versus aCSF control slices (reflecting a decrease in whole-cell conductance, *P* = 0.0008; Fig. 5g). There was no significant effect on RMP or IR in slices incubated with the other tau fragments, including the fibril core region. Given that the STA core depolarizes the RMP and increases the IR, we predicted that it should also increase the action potential firing rate (FR). Incubation with the STA core peptide significantly increased the FR versus aCSF control slices (*P* = 0.0016; Fig. 5h), so did the fibril core peptide versus aCSF controls (*P* = 0.0006; Fig. 5h). Thus, the STA core peptide impairs neuronal excitability in mouse hippocampal slices.

### The STA core peptide alters synaptic transmission

Next, we investigated whether the STA core induced changes in synaptic transmission. We found no significant differences to the stimulus input and output responses across the different tau peptides. We then examined whether there were changes to the degree of paired-pulse facilitation. In slices incubated with either the STA or the fibril core peptide, the degree of facilitation was significantly increased compared with aCSF control (*P* = 0.0190 and *P* = 0.0359 at the 100-ms interval, respectively; Fig. 5i,j). Together, these observations show that the STA core can modulate neuronal and network function.

### An assay for STAs in human CSF

We next used our findings to develop an assay to measure STAs in human CSF, using single-molecule array (Simoa) technology as we have done for other tau-based biomarkers^43–48^. The immunoassay paired two new mAbs: tau_368_ (neospecific for pathological truncation at aa 368) as capture and CT23.1 (aa 321–371) as detector. The epitopes of both antibodies fall within the STA core. Matrix-assisted laser desorption/ionization (MALDI) MS analysis demonstrated that the tau_368_ antibody is end-specific for the truncation at aa 368 (ref. ^33^) (Supplementary Fig. 12).

### STAs in the CSF separate AD from other tauopathies

**Fig. 6:**
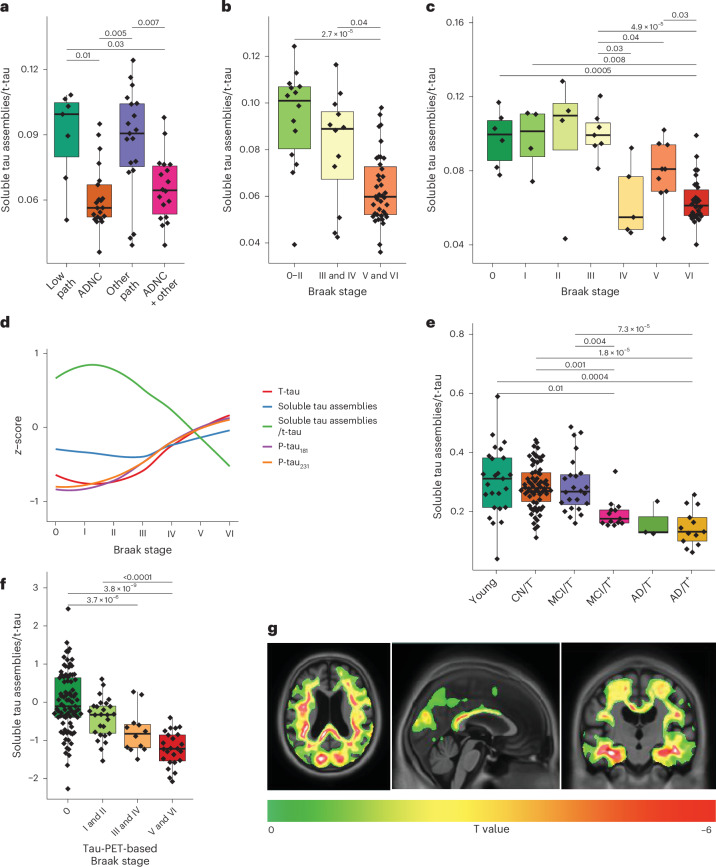
CSF levels of STAs and total tau (t-tau) ratio associate with tau NFT pathology evaluated either at autopsy or in vivo. **a**, Levels of the CSF tau STA and t-tau ratio in a well-characterized cohort with paired antemortem CSF samples and neuropathological diagnosis at postmortem (cohort 3). The groups diagnosed with ADNC (*n* = 21) and those with ADNC plus concomitant neurodegenerative pathologies (ADNC + other, *n* = 19) each had significantly lower levels of the STA and t-tau ratio versus those with low tau pathology (probably age-related tau, *n* = 8) and non-ADNC (other pathology, *n* = 19). In the box plots, the center line represents the median, the boundaries of the box are the 25th and 75th percentiles, and the whiskers extend to the furthest data value 1.5 times the interquartile range (IQR). The displayed *P* values correspond to post hoc pairwise Mann–Whitney *U*-tests with Benjamini–Hochberg false discovery rate (FDR) multiple comparison adjustment, after a significant overall Kruskal–Wallis rank-sum test (chi-squared = 17.3, d.f. = 3, *P* = 6.1 × 10^−4^). **b**, The CSF STA and t-tau ratio levels decreased with increasing Braak staging. The lowest levels were in those with the most advanced ADNC (Braak stages V and VI, *n* = 40), being significantly different from both the Braak III and IV (*n* = 13) and 0–II (*n* = 14) groups. As above, the center of the box plot indicates the median, the box boundaries indicate the 25th and 75th percentiles, and the whiskers indicate the furthest value 1.5 times the IQR. The *P* values correspond to post hoc pairwise Mann–Whitney U-tests with FDR adjustment, after a significant overall Kruskal–Wallis rank-sum test (chi-squared = 18.7, d.f. = 2, *P* = 8.5 × 10^−5^). **c**, Analyzing the STA and t-tau ratio levels in the cohort according to each of the six different Braak stages (*n* = 6 Braak stage 0; *n* = 4 (I); *n* = 4 (II); *n* = 8 (III); *n* = 5 (IV); *n* = 9 (V); *n* = 31 (VI)) highlights a sharp decrease in the ratio between Braak stages III and IV, suggesting a pathology-dependent increase in the amount of soluble tau in human CSF once affected individuals reach this disease stage. As above, the center of the box plot indicates the median, the box boundaries indicate the 25th and 75th percentiles, and the whiskers indicate the furthest value 1.5 times the IQR. The *P* values correspond to post hoc pairwise Mann–Whitney *U*-tests with FDR adjustment, after a significant overall Kruskal–Wallis rank-sum test (chi-squared = 31.6, d.f. = 6, *P* = 1.9 × 10^−5^). **d**, *z*-score model plots showing the CSF STA and t-tau ratio levels at each Braak stage relative to t-tau and STA separately, and p-tau_181_ and p-tau_231_, in the same cohort as in **c**. **e**, CSF STA and t-tau ratio levels according to diagnostic group in the tau-PET cohort (cohort 4). The STA and t-tau ratio was lowest in tau-PET^+^ AD dementia/T^+^ (*n* = 17) and highest in tau-PET^−^ young adults (young, *n* = 25), and tau-PET^−^ older adults either with CN/T^−^ (*n* = 92) or MCI/T^−^ (*n* = 30). Tau-PET^+^ individuals with MCI/T (*n* = 16) and tau-PET^−^ AD/T^−^ (*n* = 6) showed intermediate values. As above, the center of the box plot indicates the median, the box boundaries indicate the 25th and 75th percentiles, and the whiskers indicate the furthest value 1.5 times the IQR. The *P* values correspond to post hoc pairwise Mann–Whitney *U*-tests with FDR adjustment, after a significant overall Kruskal–Wallis rank-sum test (chi-squared = 41.8, d.f. = 5, *P* = 6.4 × 10^−8^). **f**, The CSF STA and t-tau ratio decreased according to tau-PET-based Braak staging (*n* = 100 (Braak stage 0), *n* = 34 (stages I and II), *n* = 16 (stages III and IV) and *n* = 31 (stages V and VI)). As above, the center of the box plot indicates the median, the box boundaries indicate the 25th and 75th percentiles, and the whiskers indicate the furthest value 1.5 times the IQR. The *P* values correspond to post hoc pairwise Mann–Whitney U-tests with FDR adjustment, after a significant overall Kruskal–Wallis rank-sum test (chi-squared = 48.6, d.f. = 3, *P* = 1.6 × 10^−10^). The plot in panel **f** is *z*-scored representation of the CSF STA/t-tau ratio normalized to the Braak 0 group as control. **g**, Voxel-wise association analyses showed inverse correlation of the STA and t-tau ratio with regional tau-PET accumulation. The voxel-wise analyses were adjusted for Aβ PET uptake, age, sex and *APOE* ε4 genotype. The *P* values for the between-group comparisons can be found in Supplementary Table 4. This figure, alongside Extended Data Figs. 5 and 6, demonstrates that there are only minute amounts of STAs in the CSF of young adults and cognitively normal older adults. However, the levels increase with disease severity such that the ratio of these tau forms to t-tau, accounting for interindividual variability in tau production and/or release, decreases with worsening NFT pathology as indexed by the Braak staging both at autopsy using immunohistochemical analysis and in vivo using tau-PET. These changes are specific to individuals with high ADNC with or without mixed neurodegenerative pathologies.

**Extended Data Fig. 5:**
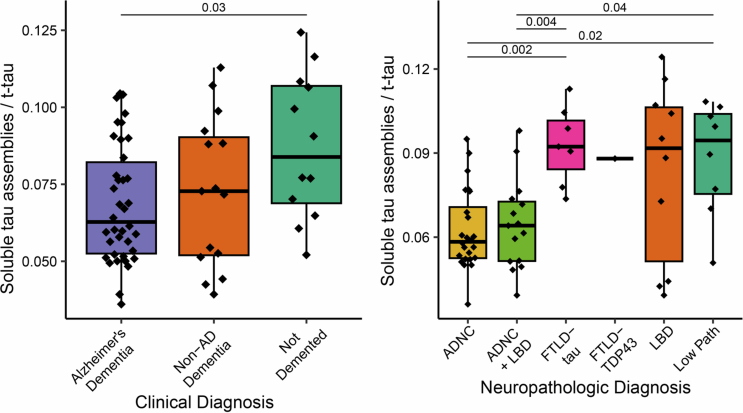
CSF soluble tau assemblies (STAs)/total-tau ratio levels in combined clinical and neuropathologic diagnostic groups in the CSF-to-autopsy cohort. (**Left**) The CSF tau STA/t-tau ratio levels were lower in those with a clinical diagnosis of Alzheimer’s Dementia (n = 29) than non-demented individuals (n = 13), but did not differ from those diagnosed with Non-AD Dementia (n = 15). For boxplots, the centre line represents the median, the bounds of the box are the 25^th^ and 75^th^ percentile, and the whiskers extend to the furthest data value within 1.5x the interquartile range (IQR). Displayed p values correspond to post-hoc pairwise Mann-Whitney U tests with Benjamini & Hochberg false discovery rate (FDR) multiple comparison adjustment, following a significant overall Kruskal-Wallis rank sum test (χ^2^ = 6.3, df = 2, p = 0.04). (**Right**) The CSF tau STA/t-tau ratio levels differed between individuals with neuropathologic consensus diagnoses of ADNC (n = 24), ADNC + LBD (n = 16), FTLD-tau (n = 7), FTLD-TDP43 (n = 1), LBD alone (n = 10), and overall Low Pathology (likely age-related tau, n = 9). As above, boxplot centre = median, box bounds = 25^th^ / 75^th^ percentile, and whiskers = furthest value within 1.5x IQR. P values correspond to post-hoc pairwise Mann-Whitney U tests with FDR adjustment, following a significant overall Kruskal-Wallis rank sum test (χ^2^ = 18.5, df = 5, p = 0.002). Abbreviations: ADNC, Alzheimer’s disease neuropathologic change; FTLD, frontotemporal lobal degeneration; LBD, Lewy body dementia; Low Path, Low Pathology; and Other Path, Other Pathology.

**Extended Data Fig. 6:**
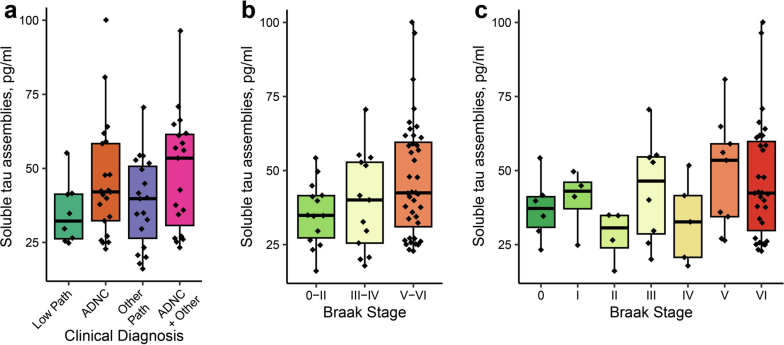
Soluble tau assemblies (STAs) in antemortem CSF samples from patients who received neuropathological diagnosis after death (cohort-3). These figures are a corollary to Fig. 6 (**a**-**c**), demonstrating effects on STAs without normalization to t-tau. (**a**) The CSF tau STA levels did not significantly differ between those with Alzheimer’s disease neuropathologic change (ADNC, n = 21), ADNC plus concomitant neurodegenerative pathologies (ADNC + Other, n = 19), low tau pathology (Low Path; likely age-related tau, n = 8), or non-Alzheimer neuropathologic change (Other Path, n = 19). Kruskal-Wallis rank sum test (χ^2^ = 4.9, df = 3, p = 0.18). (**b**) The CSF tau STA levels did not significantly differ between those with the most advanced ADNC (Braak V-VI, n = 40), those with Braak III-IV (n = 13), or those with Braak 0-II (n = 14). Kruskal-Wallis rank sum test (χ^2^ = 4.5, df = 2, p = 0.10). (**c**) The CSF tau STA levels did not significantly differ between individuals at each of the six Braak stages separately (N = 6 (Braak stage 0), N = 4 (I), N = 4 (II), N = 8 (III), N = 5 (IV), N = 9 (V), N = 31 (VI)). Kruskal-Wallis rank sum test (χ^2^ = 7.3, df = 6, p = 0.29). Abbreviations: ADNC, Alzheimer’s disease neuropathologic change; Low Path, Low Pathology; and Other Path, Other Pathology.

We evaluated the clinical performance of the CSF assay in cohort 3 (*n* = 67; Extended Data Tables 2 and 3), where each participant provided antemortem CSF samples on average 4.4 (s.d. = 2.1) years before death. To account for interindividual heterogeneity in STA levels (as in Fig. 1e), we took a ratio of STA to t-tau similar to the US Food and Drug Administration-approved Aβ_42_ and Aβ_40_ ratio assay, where Aβ_40_ normalizes for individual differences in aggregation-prone Aβ_42_ in the CSF^49^. The STA and t-tau ratio differed according to clinical diagnosis (*P* = 0.04), with post hoc comparisons demonstrating a significant difference between those with AD dementia and participants without dementia (*P* = 0.03; Extended Data Fig. 5, left). Based on standardized and expert neuropathological scoring^50^, individuals differed according to pathological diagnosis at autopsy (*P* = 0.0006), with post hoc comparisons showing that those with high AD neuropathological change (ADNC) at autopsy had similarly low CSF STA and t-tau ratio levels as those with high ADNC plus other neurodegenerative pathologies (*P* = 0.40); however, both groups had lower ratios than the participants with low ADNC pathology or other (non-ADNC) pathologies alone (*P* < 0.05 each; Fig. 6a). These results indicate that the presence of a co-pathology did not affect the results. Expanding the ‘other’ groups into the constituent diseases did not change the outcome (Extended Data Fig. 5, right). The STA and t-tau ratio significantly differed according to Braak NFT staging^1^ (*P* < 0.0001) and was significantly lower in both individuals with Braak V and VI and Braak III–IV staging versus Braak 0–II staging (*P* < 0.05; Fig. 6b), with a sharp decrease between Braak III and IV (Fig. 6c,d) suggesting that pathologically relevant changes occur in incipient AD, before isocortical association areas are affected in stages V and VI. Between-group changes for STA alone are shown in Extended Data Fig. 6.

In multivariable regression models, the STA and t-tau ratio was significantly associated with Braak NFT staging dichotomized as Braak stages 0–III (‘low’) and Braak stages IV–VI (‘high’; *t* = −7.5, *P* < 0.0001) but not with age (*t* = 1.3, *P* = 0.18), sex (*t* = −1.2, *P* = 0.24) or the time interval between CSF collection and death (*t* = 1.9, *P* = 0.06). Adding the Consortium to Establish a Registry for Alzheimer’s Disease (CERAD) score for neuritic plaques to the models did not change the correlation results (Braak IV–VI, *t* = −5.4, *P* ≤ 0.0001; neuritic plaques, *t* = 0.649, *P* = 0.51903). Similarly, adding the Thal phase of Aβ deposition in place of CERAD did not change the correlation results (Braak IV–VI, *t* = −5.9, *P* < 0.0001; Thal phase of Aβ deposition, *t* = 0.66, *P* = 0.52), suggesting that the ratio reflects central nervous system-derived CSF STA in AD independent of Aβ status.

### STAs in CSF associate with in vivo tangle pathology

We further assessed the CSF tau STA assay in an antemortem cohort with in vivo tau-PET quantification using the high-affinity [^18^F]MK6240 tracer^51,52^ (cohort 4: *n* = 185 participants; Extended Data Table 4). There were six groups including tau-PET^−^ young individuals (~20–25 years old; young/T^−^), cognitively normal (CN) older adults (CN/T^−^), and individuals with mild cognitive impairment (MCI) (MCI/T^−^) and AD dementia (AD/T^−^). The tau-PET^+^ groups included individuals with MCI (MCI/T^+^) and AD dementia (AD/T^+^). The STA and t-tau ratio was statistically different between groups (*P* ≤ 0.0001; Fig. 6e). Multiple comparisons demonstrated significantly lower (*P* < 0.05) ratio levels in AD/T^+^ versus each of the young/T^−^, CN/T^−^ and MCI/T^−^ groups. Additionally, there were significantly lower STA and t-tau ratio levels in the MCI/T^+^ group versus both the CN/T^−^ and MCI/T^−^ groups.

The STA and t-tau ratio was also examined across combined PET-based Braak NFT stages, including stages 0, I and II, III and IV, and V and VI. A Kruskal–Wallis test (*P* = 1.60 × 10^−10^) and subsequent pairwise comparisons indicated significant between-group differences. Notably, the STA and t-tau ratio was significantly higher in Braak 0 individuals compared with Braak III and IV (*P* = 0.009) and V and VI (*P* < 0.0001) individuals. Furthermore, the levels were significantly lower in the Braak V and VI versus I and II groups (*P* < 0.0001; Fig. 6f).

Significant and robust negative correlations were observed between the STA and t-tau ratio and the tau-PET radioligand [^18^F]MK6240 uptake across Braak NFT stages while accounting for Aβ PET uptake, age, sex and *APOE* ε4 genotype. These correlations were primarily observed in the entorhinal cortex, amygdala, inferior and middle temporal gyri, fusiform gyrus and parahippocampal gyrus—regions including Braak stages I and IV (Fig. 6g). Furthermore, regions associated with later Braak stages (V and VI) involving neocortical areas, including primary sensory regions, also displayed negative associations between [^18^F]MK6240 tau-PET and CSF tau STA and t-tau ratio (Fig. 6g). These findings suggest an inverse relationship between the STA and t-tau ratio and insoluble (fibrillar) brain tau pathology.

### STAs in the CSF associate with cognition

In cohort 3, the STA and t-tau ratio was positively associated with three different clinical measures of cognitive performance, that is, the Mini Mental State Examination (MMSE) (*r* = 0.41, *P* = 0.0007), the Dementia Rating Scale (*r* = 0.36, *P* = 0.003) and the Clinical Dementia Rating (CDR)-Sum of Boxes (*r* = −0.32, *P* = 0.01). Similarly, in cohort 4, the STA and t-tau ratio was associated with the MMSE scores (*r* = 0.43, *P* < 0.001; Supplementary Fig. 13).

## Discussion

Targeting early-stage STAs is a potentially effective approach to develop anti-tau diagnostics and therapies. We have shown that tau oligomers and related prefibrillar assemblies in the soluble fractions of autopsy-verified AD brains are biochemically different from those in other (primary) tauopathy brains. Next, we identified a minimal core STA peptide (~aa 258–368), revealing p-tau_262_ and p-tau_356_ aggregation-relevant phosphorylation sites. These findings have potential biomarker and therapeutic values. STA levels were higher at more advanced Braak NFT stages and correlated with a PET-detectable insoluble NFT burden specific to AD. Functional in vitro electrophysiology experiments demonstrated that a recombinantly produced STA core peptide alters synaptic transmission and neuronal excitability more potently than the insoluble fibril aggregate core, supporting the hypotheses that soluble tau might be more cytotoxic than fibrils^19,20^. Finally, we used these insights to develop a CSF biomarker of tau pre-NFT pathology and verified its clinical performance in an autopsy-verified cohort with paired antemortem CSF samples and in an antemortem cohort with [^18^F]MK6240 tau-PET imaging.

Current plasma and CSF p-tau biomarkers have modest association with brain tau pathology assessed using tau-PET^15,43,53^, suggesting different pools of tau species in brain parenchyma versus the periphery. Therefore, methods for the direct quantification of aggregation-prone tau forms in body fluids are needed. The identified STA core provides an accessible CSF biomarker that can detect small nonfibrillar tau species. Importantly, our findings indicate that p-tau sites detectable in currently used blood-based biomarker assays (for example, p-tau_181_, p-tau_205_, p-tau_212_, p-tau_217_ and p-tau_231_ (refs. ^43–45,47,54,55^)) are outside the STA core and thus fall within the fuzzy coat, corroborating our recent results that these biomarkers may not be directly associated with tau-PET in humans^53^. This clarifies why their levels start to plateau in advanced-stage AD (that is, dementia)^56^ where tau pathology is more severe and tau-PET is a better predictor of AD pathology^57,58^. It is reasonable to hypothesize that the fuzzy coat contents have less propensity to aggregate and are thus released or secreted into biofluids in the early stages of disease when NFT formation is minimal and most of the available tau species are in the soluble form. However, as STAs mature into fibrils and tangles, fewer free-floating tau fragments bearing these N-terminal to mid-region p-tau markers are available for release into biofluids because of sequestration into NFTs. Characterization of the STA core, p-tau_262_ and p-tau_356_ will thus be important in directing the development of tau aggregation-relevant biomarkers and therapies.

The polymerization of tau into STAs, and further aggregation into fibrils and NFTs, occurs through the MTBR, specifically the R2 and R3 repeat domains or even shorter hexapeptide motifs within these domains, via β-pleated sheet conformation^59–61^. The aggregation process does not require phosphorylation per se as tau proteins and peptides produced recombinantly or by chemical synthesis can polymerize into fibrils in vitro^59,61,62^. In normal full-length tau, the MTBR is masked by the N-terminal and C-terminal projection domains; therefore, because of a cysteine residue each in R2 and R3, it can form only dimers but not oligomers^63–65^. Abnormal phosphorylation at serine-262, serine-356 and related serine and threonine sites unmasks the MTBR and promotes self-aggregation^5^. While tau species lacking portions of the N-terminal and C-terminal domains through truncation can also self-aggregate, with pathologically relevant truncation at various sites reported^33,66–68^, STAs in the AD brain consisted of near-full-length peptides. These tau protein species contain the MTBR together with N-terminal, mid-region or C-terminal sequences, in agreement with previous studies^69–71^.

MS showed, beside N-terminal forms, abundance of tau species containing not only defined peptides within the MTBR but also in the mid-region in brain TBS-soluble fractions, supporting previous results in human biospecimen and cell models^72,73^. However, in the CSF, the mid-region peptides—but not the MTBR forms—remain abundant, supporting approved assays currently used in specialized clinics^74,75^ and recently developed mid-region-tau-targeting CSF assays^45,46,76–79^. Furthermore, in agreement with earlier investigations that suggested that tau forms bearing MTBR fragments were lacking in the CSF^72^, we demonstrated in this study that their levels are indeed low among tau-PET^−^ young adults and cognitively normal older adults. However, STA levels increased with disease severity, being higher in tau-PET^+^ MCI and AD dementia groups. Taking a ratio against a mid-region-targeting nonphosphorylated tau (that is, t-tau) improved this association but the direction was reversed. Importantly, our findings extend previous results in cell models showing that MTBR-containing tau becomes available extracellularly only with neuronal death, suggesting that cellular compromise is critical for MTBR tau release^73^.

Histopathological analysis indicated that p-tau_262_ and p-tau_356_ are early indicators of tau pathology in hippocampal pyramidal neurons; hyperphosphorylation promotes tau self-aggregation, with p-tau_262_ and p-tau_356_ being critical to initiate this pathological process^80,81^. Furthermore, p-tau_262_ and p-tau_356_ have been reported in AD brain extracts from several studies^69–71,80,81^; however, their relevance to NFT formation has remained unclear. We have more directly shown that p-tau_262_-immunoreactive and p-tau_356_-immunoreactive hippocampal neurons in both low and high Braak stages are not colabeled with a marker of tau fibrils (X-34) and are therefore truly representative of pre-tangles. This agrees with our observations that p-tau_262_ and p-tau_356_ immunoreactivity occurred not only in cases with advanced Braak-staged NFT pathology but also in very early cases including at Braak stage II.

Mechanistically, p-tau_262_ decreases tau binding to microtubules^82^. The anti-p-tau_262_ antibody labeled clusters of vesicles strongly resembling those reported to also express markers of granulovacuolar degeneration bodies in pre-tangles^83,84^, which probably precede fibrillar tau aggregation in classic NFTs^85^. The p-tau_356_ site is shared by both the STA and fibril core regions and might thus be an indicator of a biochemical change from soluble to insoluble aggregation. Notably, Augustinak et al.^81^ reported that a p-tau_262_ antibody preferentially detected pre-NFTs while another antibody against both p-tau_262_ and p-tau_356_ was indiscriminate for pre-NFTs, intracellular NFTs and extracellular NFTs. However, a marker of tau fibrils was not used to unequivocally identify NFTs versus pre-NFTs in that study.

The estimated core of AD-type tau fibrils differs from those in PSP, PiD and CBD brains^16^. Our results, supported by others^71^, show that these differences extend to AD-type versus non-AD STAs. Moreover, both the soluble and insoluble tau forms are surrounded by N-terminal and C-terminal fuzzy coat structures. However, the faster aggregation and higher neurotoxicity of STA versus fibril core peptides suggest unique biochemical properties. The presence of the second MTBR repeat region containing the ^275^VQIINK^280^ aggregation-promoting hexapeptide motif and the p-tau_262_ site in the STA core but not the fibril core might, at least partly, explain these results.

The STA core peptide addresses a critical need in drug development, that is, the discovery of druggable therapeutic target(s) for early-stage tau aggregates in AD. As recently shown for Aβ leading to approved therapies, targeting smaller, early-stage soluble, pre-tangle tau assemblies might have higher chances to provide clinically meaningful outcomes than insoluble fibrils. Moreover, assessments of p-tau_262_-immunoreactive and p-tau_356_-immunoreactive tau species have the potential to identify individuals with prefibrillar tau pathology who may not (yet) be tau-PET^+^. Future development of biofluid-based biomarker assays for these p-tau sites should enable identification of living individuals with this profile for mechanistic studies and inclusion in therapeutic trials for preventing tau pathology in AD.

Together, we identified a core peptide of STAs in AD brains, revealed aggregation-relevant phosphorylation sites and translated these findings to develop an accessible CSF biomarker of AD-type tau pathology that will pave the way for the quantification of early-stage soluble (prefibrillar) tau assemblies in CSF and the development of therapies against these soluble pathological entities that may not be detectable using tau-PET.

## Methods

### Tau-FRET aggregation assay

We used the homogeneous time-resolved fluorescence energy transfer tau aggregation assay (originally from Cisbio, now Revvity) which contains a tau-specific antibody conjugated to either Tb cryptate or d2, generating a FRET signal when the labeled antibodies are in proximity. The resulting signal, which is proportional to the number and complexity of aggregates in the sample, was read at the 665 nm and 615 nm wavelengths on a VICTORX4 plate reader (PerkinElmer). A negative control consisting of the two labeled antibodies in diluent (without sample) was used to calculate the Δ*F* percentage, a value that reflects the signal to background of the assay.

Initially, a dilution linearity test was performed to identify the most suitable fold dilution to use for the brain samples. The TBS-soluble fraction from the AD and control brain samples was used in a test run, first brought to the same total protein concentration of 1.317 mg ml^−1^, before a dilution series. The samples were serially diluted 1:100, 1:50, 1:10 and 1:5 in TBS, incubated overnight with the labeled antibodies added according to the manufacturer’s protocol in 96-wells low-volume white microplates. The Δ*F* percentage values were calculated using the ratio between the wavelength and the negative control provided with the kit. The observed signals were multiplied by the fold dilution and compared with the expected signals to determine the linearity of dilution.

### Expression and purification of recombinant α-synuclein and tau constructs

The DNA sequence for full-length α-synuclein and Tau_441_ (UniProt ID: P10636-8) and the tau peptides representing the STA (~tau_258–368_) and fibril (tau_302–368_) core peptides, as well as the N-terminal and C-terminal ends (tau_1–224_ and tau_368–441_, respectively) were amplified using PCR with primers representing the 5′ and 3′ sequence of each fragment, respectively, with the complementary DNA for full-length tau_441_ (cat. no. RC213312, Origene) as the template. The PCR fragments were cloned directly into the pET_SUMO vector (cohort 3: the Shiley-Marcos Alzheimer's Disease Research Center (ADRC), University of California, San Diego (UCSD)), an expression vector with a 6× His-SUMO tag N-terminally fused to the protein or peptide of interest. Constructs containing the 6× His-SUMO-tau fusion protein were sequenced and transformed into the *Escherichia coli* BL21 (DE3) strain for protein expression.

To express the SUMO fusion proteins and peptides, *E. coli* BL21 (DE3) cells harboring the construct of interest were inoculated into 20 ml lysogeny broth (LB) medium supplemented with kanamycin at a concentration of 50 µg ml^−1^ and incubated overnight to obtain a starter culture. The overnight culture was used to inoculate 1 l of LB medium with kanamycin (50 µg ml^−1^) and incubated at 37 °C. When the optical density (OD)_600_ reached 0.5–0.7, protein expression was induced with 1.0 mM isopropyl ß-d-1-thiogalactopyranoside and grown overnight at 27 °C. The next morning, the culture was centrifuged at 7,000 rpm for 20 min at 4 °C and the dry weight was calculated. The pellet was stored at −20 °C until purification.

The pellet was gently thawed at room temperature and resuspended in 1× native buffer containing 50 mM sodium phosphate, pH 8.0, and 0.5 M sodium chloride (Invitrogen) added at a ratio of 8 ml buffer to 1 g of dry weight of pellet. Lysozyme solution (Thermo Fisher Scientific) was added and the lysate was incubated on ice for 30 min, followed by sonication and then centrifugation at 12,000 rpm for 20 min at 4 °C, after which the supernatant was collected. The protein extract was added to Ni-NTA agarose columns (Novex) equilibrated with 10 mM imidazole in 1× native buffer and incubated with gentle rotation at 4 °C for 1 h. The Ni-NTA agarose column was washed with 1× native buffer containing 20 mM imidazole, and the 6× His-SUMO-tau fusion protein of interest eluted with 250 mM imidazole in 1× native buffer. The eluted protein was dialyzed against 50 mM Tris-HCl, 150 mM NaCl, pH 7.8, for 1 h; the buffer was replenished with fresh supply and the process was repeated for another hour. Dithiothreitol (DTT) (1 mM) and a SUMO protease containing a 6× His tag was added and cleavage continued overnight at 4 °C. To remove the SUMO tag and the protease, an Ni-NTA column equilibrated with 20 mM imidazole in 1× native buffer was added and incubation proceeded for 1 h at 4 °C. The flowthrough was collected and the remaining bound protein was eluted separately after adding 20 mM imidazole in 1× native buffer. Eluate fractions were dialyzed against 1× PBS; aliquots of these samples were examined using gel electrophoresis on 4–12% NuPAGE SDS gel (Invitrogen) and stained with Imperial Protein Stain (Thermo Fisher Scientific). Fractions that showed high immunoreactivity for the constructs of interest were pooled and stored at −80 °C until use, in line with previously described protocols^61,87^. Where necessary, the protein or peptide constructs were further polished using size exclusion chromatography according to published methods^61^.

### Preparation of recombinant tau aggregates

The monomeric forms of the recombinant tau peptides prepared according to the procedures described above and frozen at −80 °C in 1× PBS were used to generate the aggregates. Each tau protein variant was diluted to a final concentration of 46 µM in 1× PBS supplemented with 2 mM EDTA and incubated for 72 h on a shaking incubator (Thermomixer comfort, Eppendorf) at 350 rpm at 37 °C.

### Human postmortem tissue and CSF studies (cohorts 1–3)

Human brain tissue and CSF specimens were obtained under permission and used in accordance with the Declaration of Helsinki 2013 and the relevant ethical boards at the respective institutions. The samples were from the following sources: cohort 1, the Queen Square Brain Bank for Neurological Disorders, Department of Clinical and Movement Neurosciences, Institute of Neurology, University College London, London, UK; cohort 2, the Netherlands Brain Bank, Amsterdam, the Netherlands; and cohort 3, the Shiley-Marcos ADRC, UCSD. Ethical approval for these studies was provided by the institutional review boards (IRBs) at the participating institutions, with written consent sought for and provided by the participants or their close family members if deemed to be incapable of making such decisions at that time in accordance with IRB requirements. The Queen Square Brain Bank for Neurological Disorders has generic ethical approval from a London multicenter research ethics committee under a license from the Human Tissue Authority. The Netherlands Brain Bank cohort was approved by the ethics committee of the Vrije Universiteit Medical Center, Amsterdam. The research protocol for the UCSD cohort was reviewed and approved by the human subject review board at UCSD, while informed consent was obtained from all patients or their caregivers as consistent with California State law.

For cohort 1, we used frontal gray matter tissue samples from *n* = 50 patients, including *n* = 10 each from AD, PSP, CBD, PiD and controls to enable pathological comparison across tauopathies. Neuropathological diagnosis followed established guidelines^50,88^.

For cohort 2, the samples were taken from the superior parietal gyrus. Participants with AD were at Braak stages V and VI while controls were at Braak 0, fulfilling the criteria of Braak staging^1,2^. Complete demographic information has been published previously^29^. Briefly, age at death (~64 years), sex distribution (25% males) and the postmortem interval (~6–7 h) were similar between the two groups.

Detailed methodological description of cohort 3 has been provided previously^46,56^. Briefly, the individuals included received clinical assessment for cognitive changes annually until death and signed to allow for neuropathological examination after their death. The evaluation results were carefully assessed at a consensus conference of experts to give a research diagnosis and determine the overall evaluation of cognition (normal, MCI; diagnosed after standard criteria, or dementia)^89^.

Biofluids, including the CSF, were periodically collected from the participants who consented. In this study, we measured CSF samples from individuals with both neuropathological examination and antemortem CSF samples within 5 years of death. We included individuals with sporadic disease, excluding those with a family history of autosomal dominant AD, dominantly inherited mutations (such as *PSEN1*, *PSEN2* and *APP* mutations) or early-onset disease (under 50 years).

The autopsy procedures followed established protocols^90^. For pathological diagnosis of AD, neuritic plaques, diffuse plaques and NFT were identified either with 1% thioflavin S staining viewed with ultraviolet illumination and a 440 µm bandpass wavelength excitation filter, or with immunohistochemical staining using antibodies to Aβ (antibody 69D, rabbit polyclonal from E. Koo, 1:1,200 dilution) and PHF1 tau (from P. Davies, 1:600 dilution). Neuritic plaque density and NFT pathology were assessed according to CERAD^91^ and Braak staging^2^, respectively. For more recent cases, pathological diagnosis of AD was made using the National Institute on Aging (NIA)-Alzheimer's Association (AA) consensus criteria^50,92^. The National Alzheimer’s Coordinating Center Neuropathology Working Group^50^ recommendations were followed to stage the severity of cerebral amyloid angiopathy, grading from 0 (absent) to 3 (severe).

Lewy body pathology was evaluated using hematoxylin and eosin staining in addition to immunostaining with antibodies against α-synuclein (p-synuclein 81A from V. Lee, 1:15,000 dilution). Disease staging was performed in accordance with consensus LBD guidelines^93^. TDP-43 pathology was identified using immunohistochemical staining (polyclonal, 1:12,000 dilution, cat. no. 10782-2-AP, Proteintech).

### Homogenization and characterization of brain tissue isolates

The procedure used for cohorts 1 and 2 has been described previously^29^. Briefly, frozen brain tissue from each autopsy-verified case was dissected from the indicated region and 100 mg were dissolved in 0.5 ml TBS buffer (20 mM Tris-HCl, 137 mM NaCl, pH 7.6) containing cOmplete protease inhibitor cocktail (Roche Diagnostic). Tissue homogenization was performed on ice with TissueLyser II (QIAGEN) under the following conditions: 200-Hz frequency for 2 min. The homogenate was transferred to a new 0.5 ml TBS buffer and centrifuged at 27,000*g* for 20 min at 4 °C. The supernatant (referred to as the TBS-soluble fraction) was aliquoted and stored frozen at −80 °C. The total protein concentration in the various TBS extracts was determined using the DC Protein Assay (Bio-Rad Laboratories).

The brain tissue homogenization protocol used for the MS and immunoblotting experiments followed a protocol described in Islam et al.^30^. Both this method and the one used for cohorts 1 and 2 (which involved centrifugation of brain extracts at 135,000*g* and 27,000*g*, respectively) led to the separation of tau oligomers and tangle-free filaments (sedimentable at 235,000*g*) from monomers^6^.

### IP and depletion of brain tau

The antibodies used in these experiments are described in Supplementary Table 5. For precipitation and depletion of tau from TBS-soluble fractions of AD brain isolates, the indicated anti-tau antibodies were conjugated to Dynabeads M-280 sheep anti-mouse or anti-rabbit IgG (Thermo Fisher Scientific), respectively, depending on the origin of the antibody, and according to the manufacturer’s recommended protocol. Briefly, 10 µg of total protein from the brain extract was incubated with the Dynabeads–antibody complex (that is, 4 µg antibody added to 50 µl beads in 1× PBS) and incubated overnight at 4 °C with gentle rocking to enable even mixing. The next morning, the Dynabeads–antibody complex was recovered by using a magnetized rack, the supernatant was reincubated in new 50 µl antibody–Dynabeads conjugate and the immunoprecipation or depletion process repeated for 2 h at room temperature. Afterwards, the Dynabeads–antibody complex (the precipitate fraction) was recovered and the remaining sample (the depleted fraction) was used in the tau-homogeneous time-resolved fluorescence energy transfer assay, where 10 µl of each sample was analyzed and untreated TBS-soluble brain extract from the same patient was used as control for the depleted samples.

Tau forms precipitated on the Dynabeads captured in the Dynabeads–antibody complex were eluted from the Dynabeads with 50 µl of 0.1 M citrate buffer, pH 2.75, into tubes containing 15 µl of 1 M Tris buffer, pH 9.0, for neutralization. The Dynabead-free tau samples were analyzed using immunoblotting, a capillary-based protein separation and immunodetection assay^34^, to detect the different tau fragments present according to the manufacturer’s recommendations.

### IP–MS

The IP–MS experiments were performed at the MS facility in the Biofluid Biomarker Laboratory, Department of Psychiatry, University of Pittsburgh. Briefly, 30 µl of a pooled TBS-soluble fraction (7.1 mg ml^−1^) from the mid-temporal regions of postmortem brains was diluted to 1 ml with binding buffer (100 mM Tris-HCl, pH 7.4, 300 mM NaCl, 0.2% w/v *n*-dodecyl-ß-d-maltoside, 0.2% w/v *n*-Nonyl-β-d-thiomaltoside (cat. no. N373, Dojindo Laboratories)), supplemented with 10% v/v Neurology Panel 4-PLEX E CSF sample diluent (cat. no. 103727, Quanterix) to minimize nonspecific binding. Tau protein forms were immunoprecipitated using 50 µl of Dynabeads (M-270 Epoxy, cat. no. 14301; cohort 3: Shiley-Marcos ADRC, UCSD) conjugated with 1.25 μg of the specified antibodies (tau_12_, HT7, BT2, tau_5_, 77G7 and tau_46_), incubated overnight at 4 °C with rotation. The supernatant was then removed and the beads were washed twice with 0.5 ml PBS. After the removal of all residual liquid, proteins bound to the beads were eluted twice with 100 µl glycine buffer (50 mM glycine, pH 2.8, 0.1% *n*-dodecyl-ß-d-maltoside). The combined eluates were then neutralized with 5.5 µl 2N NaOH. Two replicate IPs were performed for each tau antibody.

Proteins were digested using SP3-based trypsin digestion^94^, similarly as described in ref. ^95^. Briefly, 50 µl of nondepleted and depleted fractions and 160 µl of precipitated fractions were brought up to 200 µl with 100 mM Tris, pH 8.0, and 2% SDS. The samples were then reduced with 10 mM DTT at 56 °C for 10 min and alkylated with 20 mM iodoacetamide at room temperature in the dark for 1 h. Subsequently, 1 ml of 100% ethanol and 30 µl of 20 mg ml^−1^ Sera-Mag SpeedBeads Carboxylate-Modified Magnetic Particles (equal mix of hydrophobic and hydrophilic beads; cat. no. 65152105050250 and cat. no. 45152105050250, GE Healthcare) were added to each sample. The mixtures were incubated at room temperature with shaking at 1,400 rpm for 20 min, followed by three washes with 80% ethanol. After removing all liquid, 1 µg of trypsin (Sequencing Grade Modified Trypsin; cat. no. V5111, Promega Corporation) in 100 µl of 50 mM ammonium bicarbonate with 1 mM CaCl_2_ was used to digest the proteins bound to the magnetic particles. After digestion, the samples were desalted with C18 spin cartridges (cat. no. SMM SS18V, The Nest Group), dried using a SpeedVac and then reconstituted in 0.1% formic acid (20 µl for nondepleted and depleted fractions and 16 µl for the precipitate).

The reconstituted peptides were analyzed using reverse-phase LC–tandem MS (MS/MS) using a nanoflow LC (a Dionex UltiMate 3000 RSLCnano System) coupled to an Orbitrap Exploris 480 mass spectrometer (Thermo Fisher Scientific). The Xcalibur software (v.2.2 SP1.48, Thermo Fisher Scientific) was used to operate the LC–MS/MS system. For each analysis, 1 µl peptides were directly injected onto a 5-cm Aurora series electrospray ionization column with a 150 µm ID filled with 1.6 μm reversed-phase C-18 packing material (120-Å pore size) (IonOpticks). Peptides were eluted using a linear gradient of 3–34% mobile phase B (0.1% formic acid in acetonitrile) in 5.5 min, then to 90% B for an additional 1 min, all at a constant flow rate of 1 µl min^−1^. Data acquisition parameters included a full MS scan from 350 to 1,600 *m/z* at a 30,000 resolution and an automatic gain control (AGC) target of 300%, followed by four data-dependent MS/MS scans at a 15,000 resolution and a standard AGC target, and a retention-time-scheduled PRM analysis of 18 tau peptides. The PRM parameters included an Orbitrap resolution of 15,000, a standard AGC target, an automatic injection time, an isolation window of 2 *m/z* and a higher-energy C-trap dissociation-normalized collision energy of 30. Supplementary Table 1 shows the targeted inclusion list with the retention-time-scheduled PRM scans. Each peptide sample was analyzed twice using LC–MS/MS.

Skyline (v.21.2.0.568) was used to facilitate the extraction of peptide quantification data from the PRM scans. To ensure accuracy, the chromatogram peak selection for each PRM assay was based on the presence of at least ten coeluting fragment ions. The final quantification of each peptide was based on the total area of the top three high-quality fragment ions (Supplementary Table 1). Replicate injections were averaged before further data analysis. The normalized intensity area ratio of peptides from the precipitate to the nondepleted fractions was used to compare the relative enrichment efficiency of tau peptides from different regions. This was achieved by dividing each ratio by the sample’s mean ratio.

### MS characterization of recombinant tau_441_ phosphorylation

Recombinant tau_441_ proteins, phosphorylated by various kinases, underwent in-solution trypsin digestion as detailed below: 1 μg of each protein was brought up to a final volume of 90 μl using 50 mM ammonium bicarbonate. This was followed by a reduction with 10 mM DTT at 56 °C for 10 min and alkylation with 20 mM iodoacetamide at room temperature in the dark for 1 h. Then, 0.25 μg of trypsin was added to each sample and the mixture was incubated overnight at 37 °C. After digestion, peptides were desalted using C18 spin cartridges, dried using SpeedVac, and reconstituted in 36 μl of 0.1% formic acid. MS analysis proceeded in a manner similar to the IP–MS experiment, with the exception that different tau peptides, as listed in Supplementary Table 2, were targeted for the PRM analysis. The Skyline software was used to extract quantitative data, similar to the IP–MS experiments.

### Biochemical characterization of the p-tau_262_ and p-tau_356_ antibodies

Sandwich ELISAs were used to validate the specificity of the p-tau_262_ and p-tau_356_ antibodies. For each measurement, 80 μl of the antibodies at 2 μg ml^−1^ in PBS, pH 7.2, was added to the well and incubated overnight at 4 °C. The well was then blocked with 200 μl PBS/0.1% BSA (cat. no. 81-053-3, Merck Millipore) for 1 h at room temperature. After blocking, the well was washed twice with 300 μl PBS with 0.05% Tween 20 (PBST). Subsequently, 50 μl of recombinant tau_441_ at concentrations ranging from 0 to 400 ng ml^−1^ were added, followed by the addition of 50 μl of PBST with 2% milk and 50 μl biotinylated tau_12_ (specific to tau_441_ aa 6–18) at 1 μg ml^−1^ in PBST. Immunocomplex formation proceeded for 1 h with gentle shaking at 300 rpm at room temperature. After incubation, the wells were washed with 300 μl PBST five times. Pierce High Sensitivity Streptavidin-HRP (cat. no. 21130, Thermo Fisher Scientific) was added and incubated for 1 h at room temperature, followed by five washes with 300 μl PBST. The 3,3′,5,5′-tetramethylbenzidine substrate (cat. no. 34022, Thermo Fisher Scientific) was added and allowed to incubate for 30 min before stopping the reaction with 100 μl of Stop solution (cat. no. N600, Thermo Fisher Scientific). The OD at 450 nm with subtraction of the background OD at 550 nm was used to determine color development. The indicated synthetic peptides at a 0.1 μg ml^−1^ concentration were added to each well for competitive ELISA during the immunocomplex formation step.

### Immunoblotting

Samples (5 μg for untreated and depleted, or precipitate from 5 μg tissue lysates after the IP procedures described above, using either PBS or the IP–MS buffer with 10% Neurology Plex 4E CSF sample diluent as the IP buffer) complemented with 1× Laemmli sample buffer (cat. no. 161-0747, Bio-Rad Laboratories) were loaded on 4–12% NuPAGE Bis-Tris gradient gels (cat. no. NP0322B0X, Thermo Fisher Scientific) and separated for 3 h at room temperature in 1× NuPAGE MOPS Running Buffer (cat. no. NP0001, Thermo Fisher Scientific) at 100 V. Separated proteins were then transferred to nitrocellulose membranes (Invitrogen iBlot 2 Transfer Stacks, cat. no. IB23002, Thermo Fisher Scientific) using the iBlot2 Western Blot Transfer System (cat. no. IB21001, Thermo Fisher Scientific) at 20 V for 5 min at room temperature. Membranes were incubated for 1 h in Intercept (PBS) Blocking Buffer (cat. no. 927-70001, LI-COR Biosciences), then overnight in the presence of the anti-tau antibody tau_12_ (1:1,000 dilution, cat. no. 806501, BioLegend) in Intercept (PBS) Blocking Buffer with 0.2% Tween 20. Membranes were then incubated with Cy3 goat anti-mouse antibody (cat. no. 115-165-166, Jackson ImmunoResearch), diluted 700× in Intercept (PBS) Blocking Buffer with 0.2% Tween 20. Immunoblots were then dried and scanned using the ChemiDoc MP system (Bio-Rad Laboratories).

### TEM

The recombinant tau aggregate preparations (5 µl) were pipetted onto copper grids that had been glow-discharged and carbon-coated, allowed 1 min to adhere onto the grid surface and then rinsed with ultrapure distilled water. Next, the grids were treated with 0.75% uranyl formate (Electron Microscopy Sciences) for 30 s to enable negative staining. TEM micrographs were taken on a Talos L120C 120 kV TEM microscope (Thermo Fisher Scientific) fitted with a BM-CETA camera-4.096 × 4.096, 14-µm pixel complementary metal–oxide–semiconductor. Microscopic imaging was performed at the Centre for Cellular Imaging at the University of Gothenburg.

For the AD brain samples, TBS-soluble homogenates were first immunoprecipitated (following the procedures described above) with the tau_12_ antibody (which has been shown to enrich for tau forms that stretch into the MTBR; Extended Data Fig. 1 and Supplementary Fig. 4) to enrich for STAs. The resulting precipitated fractions were negatively stained by treating with 1% uranyl acetate, allowed to dry and analyzed on a JEM-1400Flash TEM Microscope (JEOL) at ×25,000 direct magnification, at the Center for Biological Imaging, University of Pittsburgh.

### SPR

The SPR experiments were performed using a Biacore T100 biosensor (GE Healthcare). Immobilization of the CT19.1 antibody (epitope: aa 331–361 of tau_441_) ligand on the surface of a CM5 chip was performed at a 5 µl min^−1^ flow rate to a level of 4,000 response units using standard amino coupling reagents (Cytiva). Thereafter, the analytes (that is, the truncated tau peptides) were injected at a flow rate of 20 µl min^−1^, with the experiments being performed in PBS at 25 °C. The BIAevaluation and Prism 9 (GraphPad Software) software programs were used for data processing and presentation, respectively.

### Generation and characterization of CT antibodies

The new library of anti-tau mAbs was generated by immunizing 8-week-old BALB/c mice with 100 µg of recombinant tau_241–441_ peptide in complete Freund’s adjuvant (Sigma-Aldrich). After 2–3 further dosages of the immunogen (100 µg per mouse) in incomplete Freund’s adjuvant (Sigma-Aldrich), mice were euthanized, the spleen was removed and B cells were fused with the SP2/0 myeloma cell line according to standard protocols. Approximately 10 days after fusion, direct ELISA experiments were performed to screen the cell medium for antibodies that react with full-length recombinant tau_441_ (2N4R) or tau_241–441_. Positive clones were grown further, subcloned and subsequently frozen in liquid nitrogen. Antibody specificity was verified and the isotype determined using the Pierce Rapid Isotyping Kit-Mouse. Thereafter, mAbs were purified using a Hitrap Protein G column (Cytiva) according to the manufacturer’s instructions. Epitope mapping for each mAb was performed using direct ELISA against five custom-designed overlapping peptides spanning the tau_241–441_ sequence (Caslo ApS), specifically tau_241–291_, tau_281–331_, tau_321–371_, tau_361–411_ and tau_401–441_.

### Generation and characterization of polyclonal antibody specific for truncation at aa 368

A new polyclonal antibody specific against tau truncated at aa 368 was generated by immunizing rabbits with 200 µg of a peptide containing the tau_360–368_ sequence (Caslo ApS) in complete Freund’s adjuvant. After one more dose of the immunogen (200 µg per mouse) in incomplete Freund’s adjuvant, and two more doses of the immunogen (100 µg per mouse) in incomplete Freund’s adjuvant, the rabbits were euthanized and standard antibody generation procedures followed. Antibody validation using MS is shown in Supplementary Information.

### IHC and immunofluorescence studies

#### Cases and brain tissue samples

Studies were approved by the University of Pittsburgh Committee for Oversight of Research and Clinical Training Involving Decedents. Hippocampal tissue samples were obtained from autopsy cases at the University of Pittsburgh ADRC brain bank, including cases with NFT stage B1 or B2 and those with severe NFT stage B3 (ref. ^96^). At autopsy, samples of the hippocampus were dissected at the level of lateral geniculate nucleus and placed in cold (4 °C), 4% paraformaldehyde (cat. no. 158127-5006, Sigma-Aldrich) made in 0.01 M sodium phosphate buffer (pH 7.2) (sodium phosphate monobasic and dibasic, cat. nos. S374 and S1319, respectively, Thermo Fisher Scientific) for 48 h. After fixation, samples were sequentially immersed for 48 h in each of 15% and 30% sucrose (cat. no. S512, Thermo Fisher Scientific) solutions made in sodium phosphate buffer. Samples were sliced on a freezing, sliding microtome (model 860, American Optical Corporation) into 40-μm-thick sections that were stored at −20 °C in cryoprotectant solution^97^.

#### IHC and immunofluorescence

Tissue sections were removed from the cryoprotectant solution and rinsed three times in 0.1 M Trizma-buffered saline containing 0.25% Triton X-100 (Trizma, pH 7.4, cat. no. T7693, Sigma-Aldrich; Triton X-100, cat. no. T9284, Sigma-Aldrich). Chromogen-based IHC was performed as described previously^98^ using the VECTASTAIN Elite ABC-HRP Kit (cat. no. PK-6100, Vector Laboratories) with Ni-enhanced 3,3′-diaminobenzidine tetrahydrochloride (cat. no. D8001, Sigma-Aldrich) as the chromogen. The details of the primary antibodies, including primary antibodies targeting an epitope at p-tau_202/205_ (clone AT8, 1:3,000 dilution for chromogen-based IHC and 1:500 dilution for the multi-immunofluorescence experiments, cat. no. MN1020, Thermo Fisher Scientific), an epitope at p-tau_231_ (clone AT180, 1:1,000 dilution for chromogen-based IHC, cat. no. MN1040, Thermo Fisher Scientific), an epitope at p-tau_262_ (1:500 dilution for chromogen-based IHC and 1:200 dilution for the multi-immunofluorescence experiments, cat. no. 44-750G, Thermo Fisher Scientific) and an epitope at p-tau_356_ (1:750 dilution for chromogen-based IHC and 1:250 dilution for the multi-immunofluorescence experiments, cat no. 44-751G, Thermo Fisher Scientific) are given in Supplementary Table 6. Secondary antibodies (all used at 1:250 dilution) included a biotinylated goat anti-mouse IgG (cat. no. 115-065-146, Jackson ImmunoResearch), a biotinylated goat anti-rabbit IgG (cat. no. 111-065-045, Jackson ImmunoResearch), an Alexa Fluor 594-conjugated goat anti-mouse IgG (cat. no. 115-585-146, Jackson ImmunoResearch) and an Alexa Fluor 488-conjugated goat anti-rabbit IgG (cat. no. 111-585-144, Jackson ImmunoResearch). X-34 staining was performed as described previously^99^. Light microscopy analysis was performed using an Olympus BX53 microscope. The immunofluorescence analysis was performed as described previously^100^, using the Olympus BX53 microscope connected to a fluorescence illuminator (X-Cite 120Q). The microscope was equipped with an Olympus DP72 digital camera connected to a Dell Precision T5500 Desktop Workstation running the Olympus cellSens Standard v.1.12 imaging software, and with a U PLAN S-APO ×4 objective (numerical aperture (NA) 0.16), a U PLAN S-APO ×10 objective (NA 0.4) and a U PLAN S-APO ×20 objective (NA 0.75). The fluorescence of the Alexa Fluor 488 fluorophore was visualized using a fluorescein isothiocyanate-compatible filter (excitation peak = 480 nm, beam splitter = 505 nm, emission peak = 535 nm; cat. no. 41001, Chroma). The fluorescence of the Alexa594 fluorophore was visualized using a Texas red isothiocyanate-compatible filter (excitation peak 535 nm, beam splitter 565 nm, emission peak 610 nm; #41002, Chroma). The fluorescence of X-34 was visualized using a violet filter set (excitation peak = 405 nm, dichroic mirror DM440, emission peak = 455 nm; cat. no. 11005, Chroma).

### Electrophysiology experiments

#### Preparation of mouse brain slices

All animal care and experimental procedures were reviewed and approved by the institutional animal welfare and ethical review body at the University of Warwick. Animals were kept in standard housing with littermates, provided with food and water ad libitum and maintained on a 12:12 (light–dark) cycle. Male and female 3–4-week-old C57BL/6 mice were euthanized using cervical dislocation and decapitated in accordance with the UK Animals (Scientific Procedures) Act 1986. The brain was rapidly removed and acute parasagittal or horizontal brain slices (350–400 μM) were cut with a Microm HM 650V microslicer in cold (2–4 °C) high Mg^2+^, low Ca^2+^ aCSF, consisting of the following: 127 mM NaCl, 1.9 mM KCl, 8 mM MgCl_2_, 0.5 mM CaCl_2_, 1.2 mM KH_2_PO_4_, 26 mM NaHCO_3_ and 10 mM d-glucose (pH 7.4 when bubbled with 95% O_2_ and 5% CO_2_, 300 mOsm). Slices were stored at 34 °C in standard aCSF (1 mM Mg^2+^ and 2 mM Ca^2+^) for 1–8 h.

#### Incubation of acute brain slices with recombinant tau truncations

After at least 1 h of recovery, slices were either incubated in aCSF (control) or in 444 nM recombinant tau (1–224; N terminus, 258–368; STA core, 302–368; fibril core or 368–441; C terminus) in aCSF for 1 h, in bespoke incubation chambers at room temperature. The incubation chambers consisted of small, raised grids (to allow perfusion of slices from above and below) placed in the wells of a 24-well plate (Falcon), which were bubbled with 95% O_2_, 5% CO_2_ using microloaders (Eppendorf). Slices were placed into the incubation chambers one at a time (minimum volume 1.5 ml to cover the raised grid). Individual slices were then placed on the recording rig and perfused with regular aCSF throughout the recording period, so the recombinant tau was only present for the 1-h incubation, as shown previously^101^.

#### Whole-cell patch clamp recording from single hippocampal CA1 pyramidal neurons

A slice was transferred to the recording chamber, submerged and perfused (2–3 ml min^−1^) with aCSF at 30 °C. Slices were visualized using infrared IR differential interference contrast optics with an Olympus BX151W microscope (Scientifica) and a charge-coupled device camera (Hitachi). Whole-cell current clamp recordings were made from pyramidal cells in area CA1 of the hippocampus using patch pipettes (5–10 mΩ) manufactured from thick-walled glass (Harvard Apparatus). Pyramidal cells were identified by their position in the slice, morphology (from fluorescence imaging) and characteristics of the standard step current–voltage relationship. Voltage recordings were made using an Axon Multiclamp 700B amplifier (Molecular Devices) and digitized at 20 kHz. Data acquisition and analysis were performed using pClamp 10 (Molecular Devices). Recordings from neurons that had an RMP of between −55 and −75 mV at whole-cell breakthrough were accepted for analysis. Bridge balance was monitored throughout the experiments; any recordings where it changed by more than 20% were discarded.

#### Stimulation protocols

To extract the electrophysiological properties of recorded neurons, both step and naturalistic, fluctuating currents were injected.

##### Standard IV protocol

A standard current–voltage relationship was constructed by injecting standard (step) currents from −200 pA, incrementing by either 50 or 100 pA (1-s duration) until a regular firing pattern was induced. A plot of step current against voltage response around the resting potential was used to measure the infrared (from the gradient of the fitted line).

##### Dynamic IV protocol

A dynamic-I-V curve, defined by the average transmembrane current as a function of voltage during naturalistic activity, can be used to efficiently parameterize neurons. The method has been described previously. Briefly, a current waveform, designed to provoke naturalistic fluctuating voltages, was constructed using the summed numerical output of two Ornstein–Uhlenbeck processes^86,102,103^ with time constants tfast = 3 ms and tslow = 10 ms. This current waveform, which mimics the stochastic actions of α-amino-3-hydroxy-5-methyl-4-isoxazolepropionic acid and gamma-aminobutyric acid receptor channel activation, is injected into cells and the resulting voltage recorded (a fluctuating, naturalistic trace). The voltage trace was used to measure the frequency of action potential firing and to construct a dynamic-I-V curve. The FR was measured from voltage traces evoked by injecting a current waveform of the same gain for all recordings (to give an FR of ∼2–3 Hz). Action potentials were detected by a manually set threshold and the interval between action potentials was measured. All analyses were completed using either the MATLAB or Julia (v.1.7.3) software platforms^104^.

#### Extracellular recording of synaptic transmission

A 400-µM parasagittal slice was transferred to the submerged recording chamber and perfused with aCSF at 4–6 ml min^−1^ (32 °C). The slice was placed on a grid allowing perfusion above and below the tissue; all tubing (Tygon) was gas-tight to prevent loss of oxygen. To record field excitatory postsynaptic potentials (fEPSPs), an aCSF-filled microelectrode was placed on the surface of the stratum radiatum in CA1. A bipolar concentric stimulating electrode (FHC) controlled by an isolated pulse stimulator model 2100 (AM Systems) was used to evoke fEPSPs at the Schaffer collateral–commissural pathway. fEPSPs were evoked every 30 s (0.03 Hz). Stimulus input and output curves for the fEPSPs were generated using a stimulus strength of 2–80 mA for all slices (stimulus duration 200 µs). Signals were filtered at 3 kHz and digitized online (10 kHz) with a Micro CED (mark 2) interface controlled by the Spike software (v.6.1) (Cambridge Electronic Design). The fEPSP slope was measured from a 1-ms linear region following the fiber volley.

### Development and analytical validation of the CSF STA assay

To capture STAs, a rabbit polyclonal antibody targeting an end-specific truncation at aa 368 was coupled to paramagnetic beads (cat. no. 103207, Quanterix), while the detection antibody CT23.1 (epitope: aa 321–371) was conjugated to biotin (cat. no. A3959, Thermo Fisher Scientific) according to the manufacturer’s recommendations. The resulting method was a three-step Simoa assay that combined the assay beads (that is, beads conjugated with the capture antibody) and the helper beads in a 70% to 30% ratio to give 20,000 beads per µl and 1 µg ml^−1^ of biotin-conjugated detection antibody with 100 µl of undiluted CSF. The average number of enzymes per bead signal for each sample was plotted against the concentration of the inputted biospecimen.

The specificity of the capture antibody to the truncation at tau_368_ was confirmed with MALDI MS. In this experiment, 8 μg of the polyclonal 368 antibody was added to 50 μl M-280 Dynabeads (sheep anti-rabbit IgG, Invitrogen) per sample according to the manufacturer’s product description. The 368-coated beads were used for IP of either the positive control or antigen (aa ITHVPGGGN equivalent to aa 359–368 with truncation at aa 368) or the negative control (aa GSLDNITHVPGGGNKKIETHKLTFRE 355–380 lacking truncation at aa 368) in PBS. Beads and samples were transferred to a KingFisher magnetic particle processor (polypropylene tubes, Thermo Fisher Scientific) for automatic washing and elution of full-length and truncated peptides. Eluted samples were collected and dried in a vacuum centrifuge and redissolved in 5 μl 0.1% formic acid in 20% acetonitrile and subsequently analyzed using a Bruker Daltonics UltrafleXtreme MALDI/ionization time-of-flight/time-of-flight mass spectrometer (Bruker Daltonics).

Clinical validation was performed using CSF samples from the CSF-to-autopsy and tau-PET studies (cohorts 3 and 4).

### Tau-PET cohort

#### Study participants

Participants (*n* = 185) included in the study were selected from the Translational Biomarkers of Aging and Dementia (TRIAD) cohort, McGill University, Canada^43,105^. These participants had undergone Aβ and tau-PET imaging, the core CSF biomarker (Aβ_42/40_, p-tau_181_ and t-tau) analyses and had a CSF sample available for tau aggregate quantification.

In the TRIAD cohort, CN participants were defined as having an MMSE score greater than 24 and a CDR score of 0. This group included both young individuals (younger than 30 years) and older adults (older than 55 years). Participants with MCI had a CDR score of 0.5, with subjective and objective impairments in cognition, while their activities of daily living were preserved. Patients with AD dementia met the diagnostic criteria of the NIA and AA, and had a CDR score greater than or equal to 0.5 (ref. ^106^).

All participants provided written informed consent; the research protocol was approved by the Montreal Neurological Institute PET working committee and the Douglas Mental Health University Institute Research Ethics Board.

#### Brain imaging

[^18^F]AZD4694 and [^18^F]MK6240 PET were used to assess brain Aβ and tau pathologies, respectively. Imaging was acquired at two time points, that is, 40–70 min and 90–110 min after injection. PET scans were conducted using a Siemens High Resolution Research Tomograph (Siemens Medical Solutions).

To process the imaging data, PET scans from each participant were combined with their magnetic resonance imaging data. The cerebellar gray matter and the inferior cerebellar gray matter were used as reference regions for calculating the standard uptake value ratio (SUVR) for amyloid-β and tau-PET, respectively.

Aβ positivity was determined as a global [^18^F]AZD4694 SUVR equal to or greater than 1.55 (ref. ^107^). For tau-PET, a global index of tau pathology was obtained by calculating the average SUVR in the temporal meta-region of interest. Tau positivity was then defined as an SUVR equal to or greater than 1.24 (ref. ^108^). Moreover, participants were categorized into PET-based Braak stages based on the topography of tau-PET abnormality, as described in previous studies^12^.

### Statistical methods, data analysis and software

All statistical tests were two-sided.

#### Tau-FRET studies

Data were presented as the mean ± s.e.m. Two groups were compared with the Mann–Whitney *U*-test, whereas a Kruskal–Wallis analysis of variance with Dunn’s multiple comparison test was used to examine three or more groups in Prism 9.

#### Electrophysiology studies

Prism 9 was used. Because of the small sample sizes (*n* < 15), statistical analysis was performed using nonparametric methods, that is, Kruskal–Wallis analysis of variance, Mann–Whitney *U*-test and Wilcoxon signed-rank test as required. All data are presented as the mean ± s.e.m. with individual experiments represented by single data points. For all experiments, significance was set at *P* ≤ 0.05. Data points for each experimental condition were derived from a minimum of four individual animals.

#### CSF-to-autopsy study (cohort 3)

Statistical analyses were performed using R v.4.3.1. The nonparametric Kruskal–Wallis rank-sum test was used for comparisons between group categories in demographic tables and figures alike, with significant results followed by post hoc pairwise Mann–Whitney *U*-tests with Benjamini–Hochberg FDR adjustment for multiple comparisons. Categorical variables were compared using a chi-squared test, with significant results followed by pairwise chi-squared tests with FDR adjustment for multiple comparisons.

#### Tau-PET cohort studies (cohort 4)

Python v.3.11.2 was used to perform nonimaging statistical analyses. For several data processing and statistical tasks, several additional packages were used. Pandas (v.1.5.3) was used as a powerful data analysis tool, providing data structures like DataFrames and Series, which allowed for efficient data handling and transformation. NumPy (v.1.24.2) was used for numerical computations, enabling the manipulation of multidimensional arrays and matrices. Scikit-learn (v.1.2.2) was used for regression, clustering and model evaluation. Statsmodels (v.0.13.5) was used for statistical modeling and hypothesis testing.

To assess the overall differences among the multiple diagnostic categories, a Kruskal–Wallis test was performed. If the Kruskal–Wallis test yielded a significant result, indicating that there were overall differences among the groups, post hoc pairwise comparisons were conducted using a Mann–Whitney *U*-test. To control the increased risk of type I error because of multiple pairwise comparisons, a Bonferroni correction was applied to adjust the significance threshold (alpha) for each comparison and maintain an overall alpha level for the entire set of tests.

Correlation analyses were performed to examine the relationships between a set of selected variables. Specifically, the Pearson correlation coefficient was calculated to measure the linear associations between continuous variables. To control the risk of type I errors arising from multiple comparisons, FDR correction was applied to adjust the *P* values. Only correlations with an FDR-corrected *P* *<* 0.05 were considered statistically significant and included in the final correlation matrix. Significant correlations were visualized using a heatmap, with color intensity representing the strength and direction of the associations between variables.

Neuroimaging analyses were carried out using the VoxelStats toolbox (https://github.com/sulantha2006/VoxelStats), a MATLAB‐based analytical framework that allows for the execution of multimodal voxel‐wise neuroimaging analyses^109^. Using this toolbox, a voxel-wise linear model was constructed to assess the relationship between the CSF STA and t-tau ratio and [^18^F]MK6240 PET SUVR while correcting for age, sex, the presence of *APOE* ε4 and [^18^F]AZD4694 PET SUVR, resulting in a *t*-map to display the strength of this relationship in a voxel-wise manner across different brain regions.

### Reporting summary

Further information on research design is available in the Nature Portfolio Reporting Summary linked to this article.

## Online content

Any methods, additional references, Nature Portfolio reporting summaries, source data, extended data, supplementary information, acknowledgements, peer review information; details of author contributions and competing interests; and statements of data and code availability are available at 10.1038/s41591-024-03400-0.

## Supplementary information

Supplementary Information Supplementary Figs. 1–13 and Tables 1–6.

Reporting Summary

## Figures and Tables

**Extended Data Table 1:**
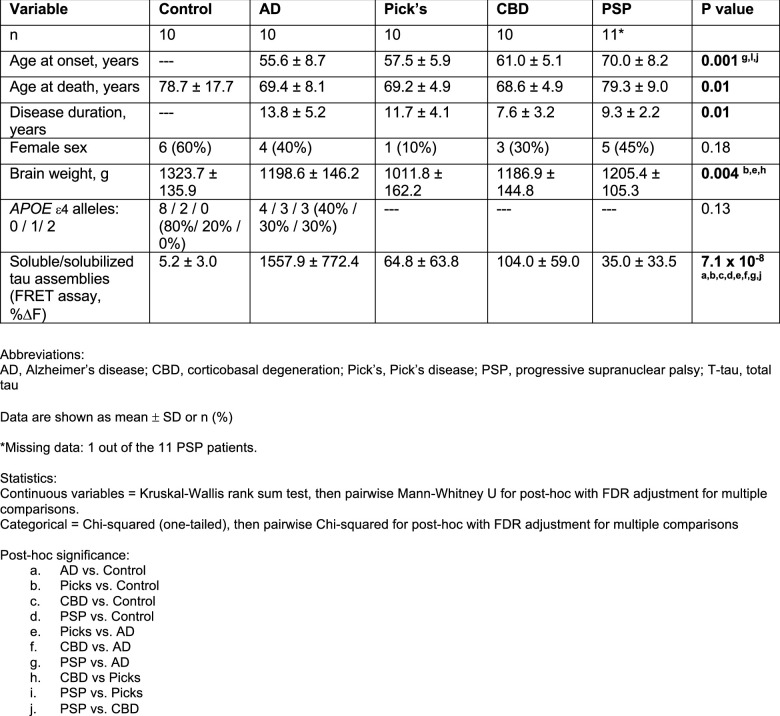
Demographic characteristics of the autopsy-verified tauopathy brain tissue cohort (cohort 1)

**Extended Data Table 2:**
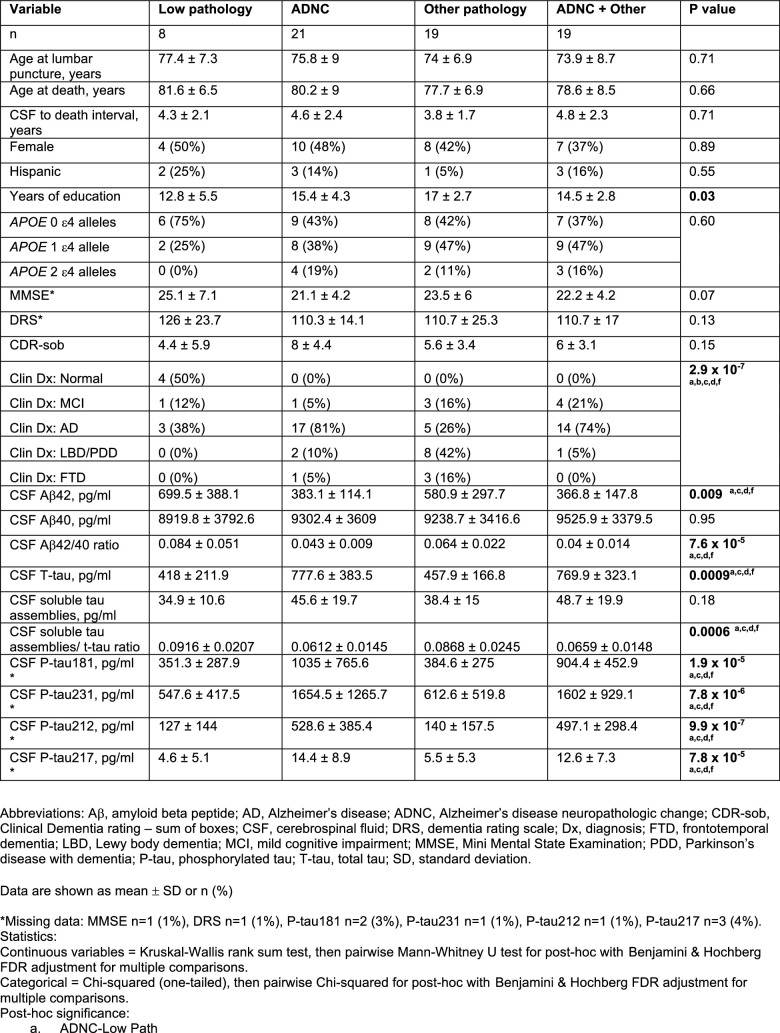
Demographic, clinical and biomarker characteristics of the antemortem CSF to autopsy cohort (cohort 3)

**Extended Data Table 3:**
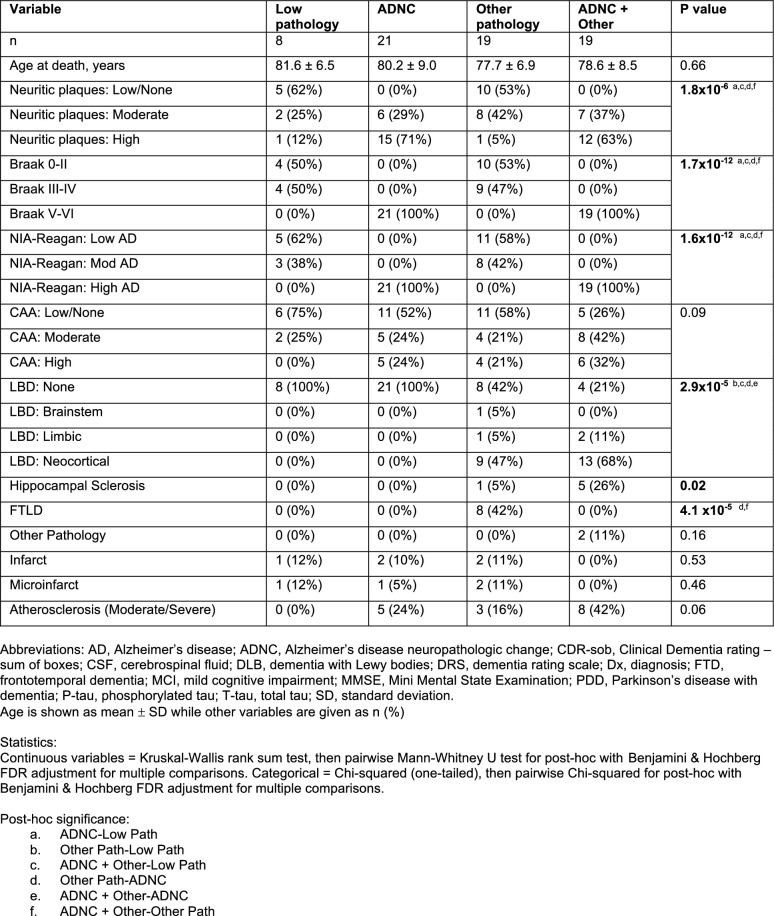
Neuropathological characteristics of the antemortem CSF to autopsy cohort (cohort 3)

**Extended Data Table 4:**
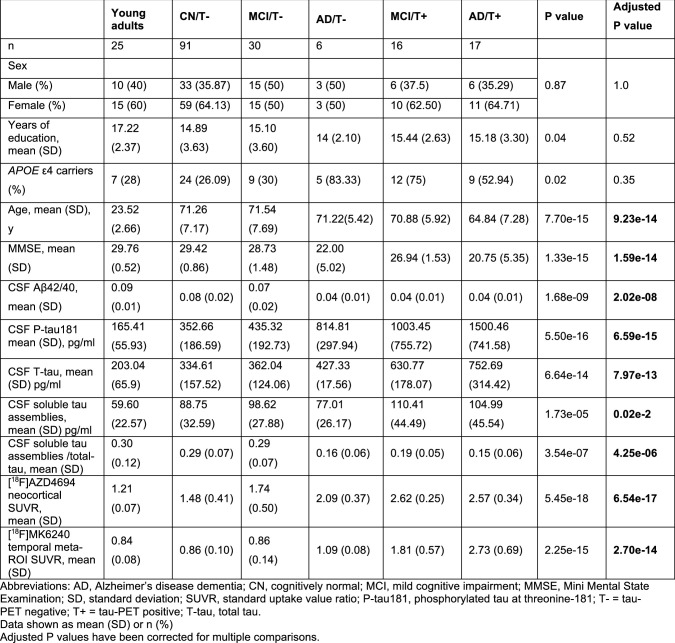
Demographic and biomarker characteristics of the cohort with paired CSF samples and tau-PET data (cohort 4)

## Data Availability

The UniProt database was used to search for the tau protein sequence (UniProt ID: P10636-8). De-identified data generated in this study can be shared with qualified and identifiable investigators for the purpose of replicating the results and procedures in the study. Requests can be made to the corresponding author of the present study (T.K.K.) who will refer them to the respective cohort principal investigators where necessary. Requests will be reviewed by the investigators and respective institutions to ensure that each data request and transfer are in agreement with UK, EU, Canada and US legislation on general data protection or is subject to any intellectual property or confidentiality obligations. The purpose of these procedures is to ensure participant anonymity and ensure data safeguarding limited to the terms set forth in the IRB approvals. Data request can be made directly at https://triad.tnl-mcgill.com/ (for TRIAD), https://www.adrc.pitt.edu/for-researchers/adrc-data-resources/ (for Pittsburgh ADRC) and https://neurosciences.ucsd.edu/centers-programs/adrc/professionals/resources/index.html (UCSD ADRC).

## Code availability

No new specialized code was written specifically for this study. Previously established code used has been appropriately referenced in the article.
