# Supplementary material for: Phospho-tau serine-262 and serine-356 as biomarkers of pre-tangle soluble tau assemblies in Alzheimer’s disease
Source: Nat Med. 2025 Feb 10;31(2):574–88. doi: 10.1038/s41591-024-03400-0 (PMC11835754; doi:10.1038/s41591-024-03400-0)
Supplement: Supplementary file 1 — Supplementary Figs. 1–13 and Tables 1–6. [file 41591_2024_3400_MOESM1_ESM.pdf]

nature medicine

Supplementary information https://doi.org/10.1038/s41591-024-03400-0

Phospho-tau serine-262 and serine-356 as biomarkers of pre-tangle soluble tau assemblies in Alzheimer's disease

In the format provided by the authors and unedited

Supplementary Appendix to:

Phospho-tau serine-262 and serine-356 as biomarkers of pre-tangle soluble tau assemblies in Alzheimer's disease

Tohidul Islam[1*], Emily Hill[2*], Eric E. Abrahamson[3,4*], Stijn Servaes[5,6], Denis S. Smirnov[7,8], Xuemei Zeng[9], Anuradha Sehrawat[9], Yijun Chen[9,10], Przemysław R. Kac[1], Hlin Kvartsberg[1,11], Maria Olsson[1,11], Emma Sjons[1,11], Fernando Gonzalez-Ortiz[1,11], Joseph Therriault[5,6], Cécile Tissot[5,6], Ivana Del Popolo[2], Nesrine Rahmouni[5,6], Abbie Richardson[2], Victoria Mitchell[2], Henrik Zetterberg[1, 11,12,13,14,15], Tharick A. Pascoal[3,9], Tammaryn Lashley[16], Mark J. Wall[2], Douglas Galasko[7], Pedro Rosa-Neto[5,6], Milos D. Ikonomovic[3,4,9#], Kaj Blennow[1,11#], Thomas K. Karikari[1,9#]

[1]Department of Psychiatry and Neurochemistry, Institute of Neuroscience and Physiology, The Sahlgrenska Academy at the University of Gothenburg, Mölndal, 431 80, Sweden
[2]School of Life Sciences, University of Warwick, Coventry, CV4 7AL, UK
[3]Department of Neurology, School of Medicine, University of Pittsburgh, Pittsburgh, PA, 15213, USA
[4]Geriatric Research Education and Clinical Center, VA Pittsburgh HS, Pittsburgh PA, USA
[5]Translational Neuroimaging Laboratory, McGill University Research Centre for Studies in Aging, Alzheimer's Disease Research Unit, Douglas Hospital Research Institute, Le Centre intégré universitaire de santé et de services sociaux (CIUSSS) de l'Ouest-de-l'Île-de-Montréal, Montréal, Québec, Canada
[6]Department of Neurology and Neurosurgery, McGill University, Montreal, Québec, Canada
[7]University of California, San Diego and Shiely-Marcos Alzheimer's Disease Research Center, La Jolla, CA 92037, USA
[8]Pathology Residency Program, Mass General and Brigham and Women's Hospitals, Harvard Medical School, Boston, MA, USA
[9]Department of Psychiatry, School of Medicine, University of Pittsburgh, Pittsburgh, PA, 15213, USA
[10]Department of Chemistry, School of Medicine, University of Pittsburgh, Pittsburgh, PA, 15213, USA
[11]Clinical Neurochemistry Laboratory, Sahlgrenska University Hospital, Mölndal, Sweden
[12]Department of Neurodegenerative Diseases, UCL Queen Square Institute of Neurology, London, UK
[13]UK Dementia Research Institute, University College London, London, UK
[14]Hong Kong Center for Neurodegenerative Diseases, Hong Kong, China
[15]Department of Medicine, School of Medicine and Public Health, University of Wisconsin-Madison, Wisconsin, USA
[16]Department of Neurodegenerative Disease, Queen Square Institute of Neurology, University College London, London, UK

*These authors contributed equally
[#] These authors jointly supervised this work
Corresponding author: Dr. Karikari: Department of Psychiatry, School of Medicine, University of Pittsburgh, Pittsburgh 15213, PA, USA. Email: Karikaritk@upmc.edu

Supplementary Figures

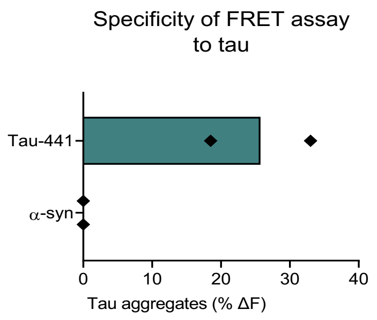

Supplementary Figure 1. Specificity of the tau-FRET assay. Binding profiles of the FRET assay against recombinant full-length tau-441 and recombinant alpha-synuclein suggest specificity to tau. N=2 biological replicates, each consisting of two replicates and performed on different days.

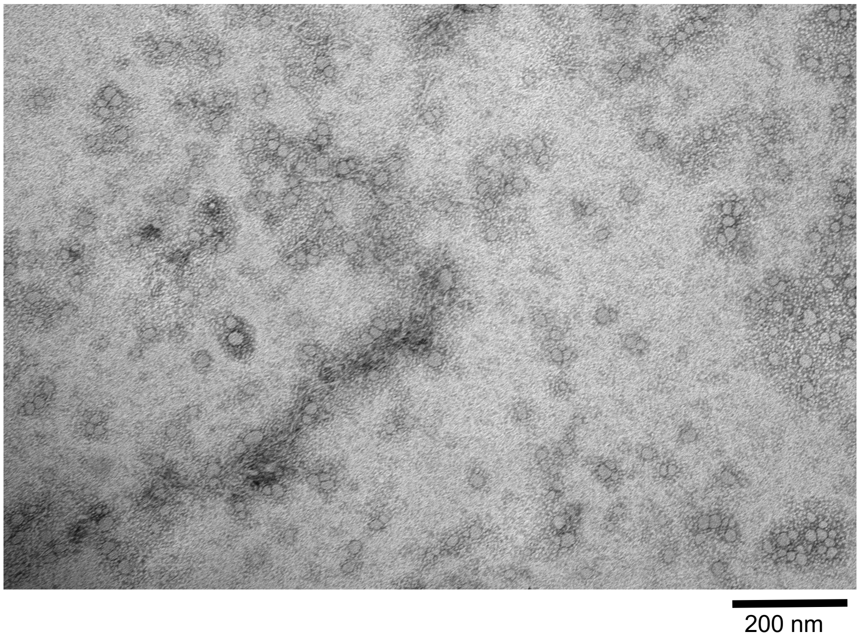

Supplementary Figure 2. **Negative-stain transmission electron microscopy image of Tris-buffered saline (TBS)-soluble fraction of an AD brain tissue**. The sample was immunoprecipitated with the antibody Tau12 (shown in Extended Data Figure 1 and Supplementary Figure 3 to pull tau peptides/forms including those in the MTBR) to enrich for tau forms which were then analyzed by electron microscopy. The image shows granular/spherical oligomers as well as emerging tangle-free pre-fibril-like structures.
The micrograph is a representative image from six technical replicates.

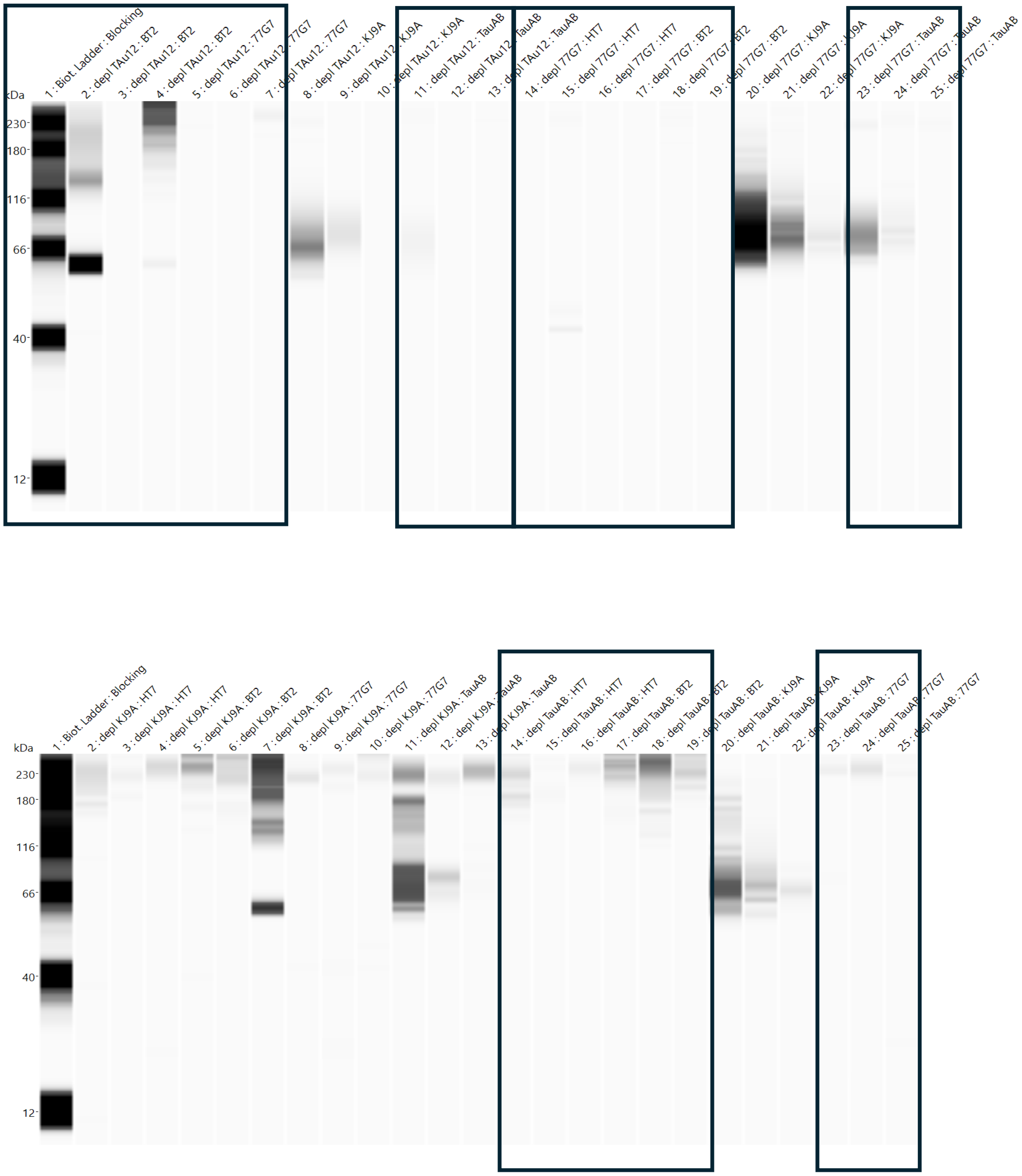

Supplementary Figure 3. Source uncropped WES blotting images used in Figure 2d in the main text. Boxes have been put around the panels used in the main text. Lane labels shown as “depl antibody X: antibody Y” indicate that antibody X was used for immunodepletion while antibody Y was used for detection. Note that data obtained using the K9JA polyclonal antibody the binding epitope(s) of which is/are not clearly known were excluded from the main text images. The results are representative of three biological replicates.

IP with Tau12 antibody

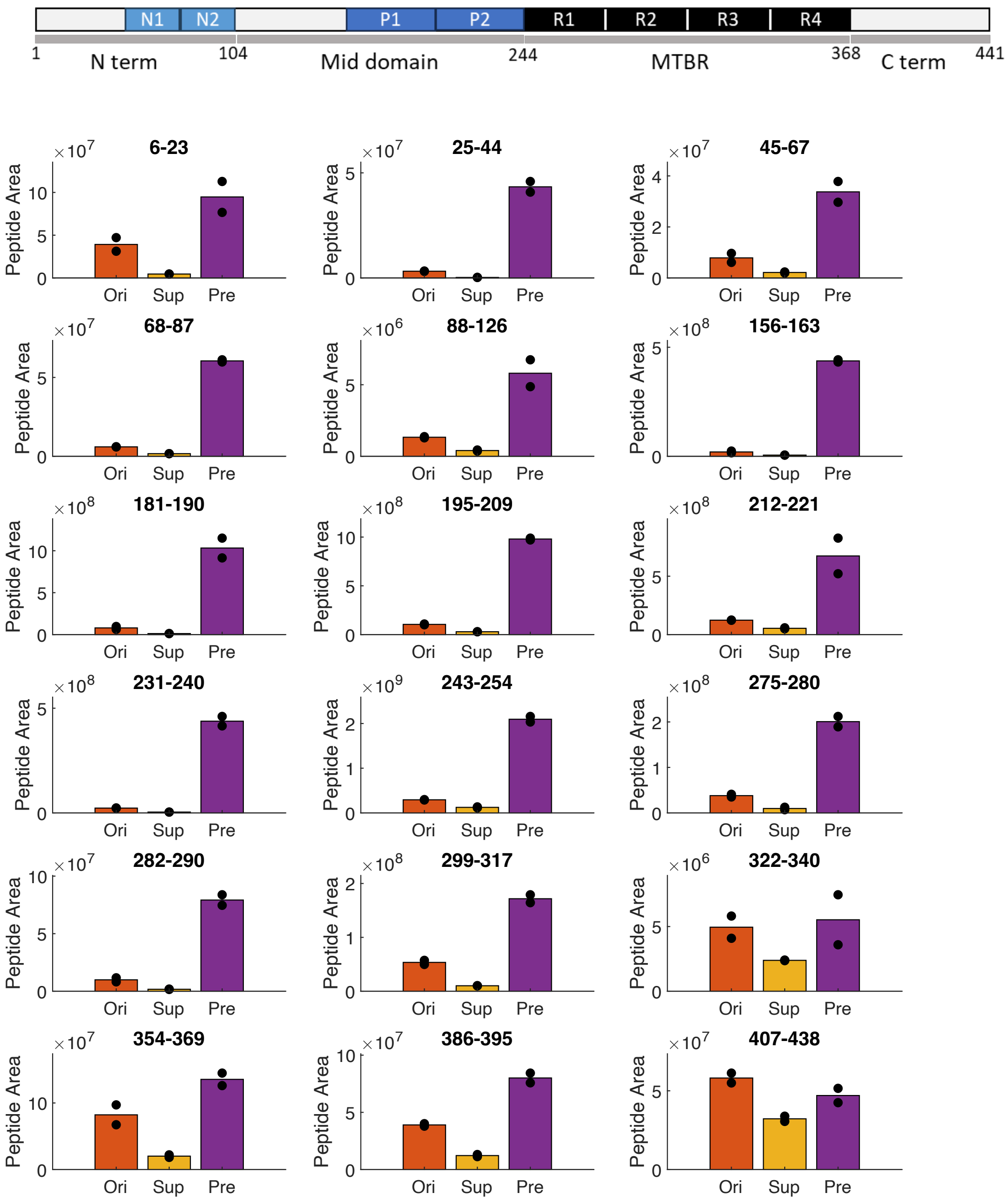

Supplementary Figure 4. The abundance of tau peptides in non-depleted original ("Ori"), depleted supernatant ("Sup"), and precipitate ("Pre") fractions in a pooled TBS soluble brain extract immunoprecipitated with the antibody Tau12 (epitope: aa6-18). The peak area of each tau peptide, derived from a PRM assay on the Orbitrap Exploris™ 480, served as a surrogate for peptide abundance. The structural features of tau441 are illustrated at the top of the figure. Each bar graph's title indicates the start and end positions of the peptide in tau441. N=2 biological replicate IPs.

IP with HT7 antibody

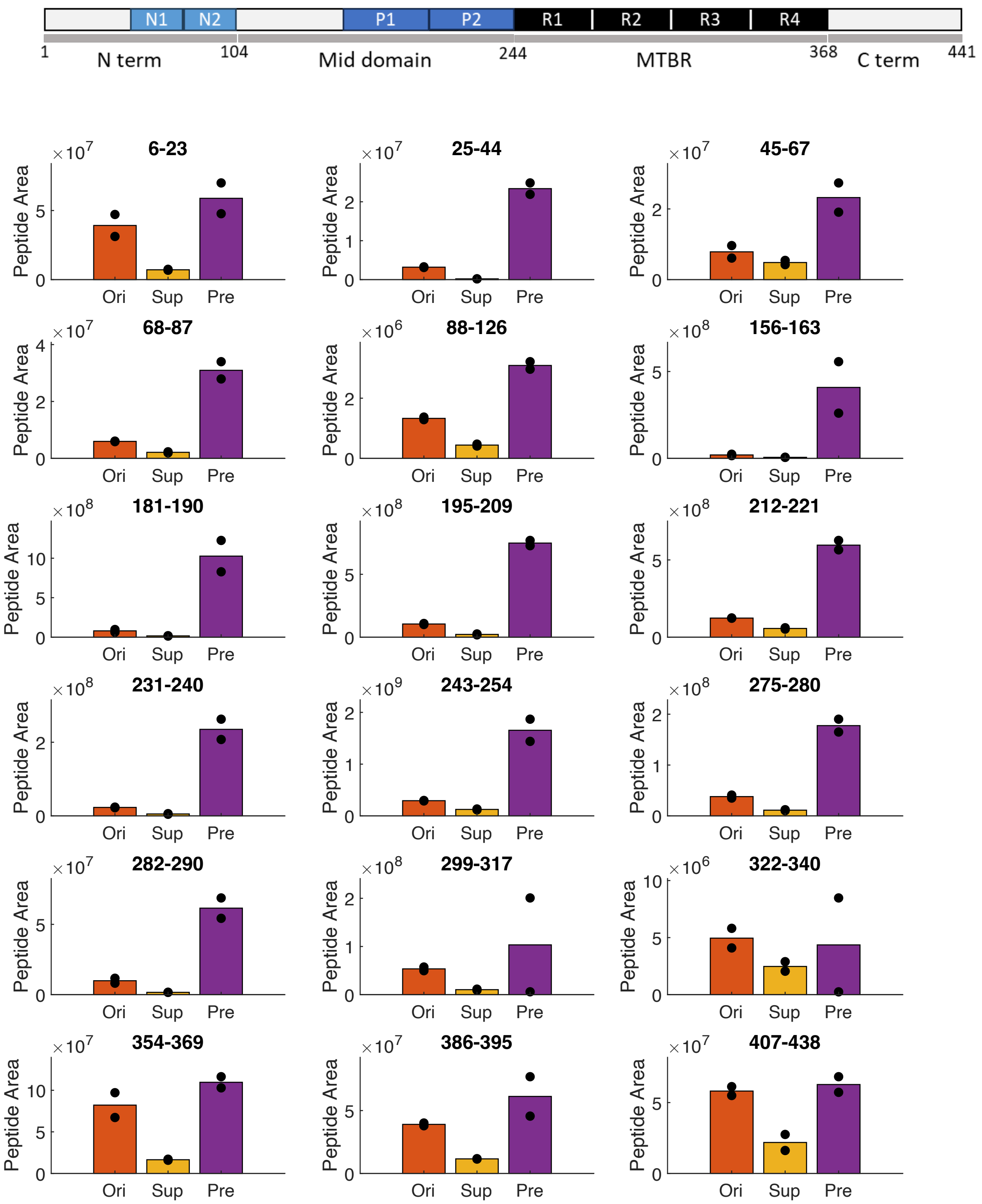

Supplementary Figure 5. The abundance of tau peptides in non-depleted original ("Ori"), depleted supernatant ("Sup"), and precipitate ("Pre") fractions in a pooled TBS soluble brain extract immunoprecipitated with the antibody HT7 (epitope: aa159-163). The peak area of each tau peptide, derived from a PRM assay on the Orbitrap Exploris™ 480, served as a surrogate for peptide abundance. The structural features of tau441 are illustrated at the top of the figure. Each bar graph's title indicates the start and end positions of the peptide in tau441. N=2 biological replicate IPs.

IP with BT2 antibody

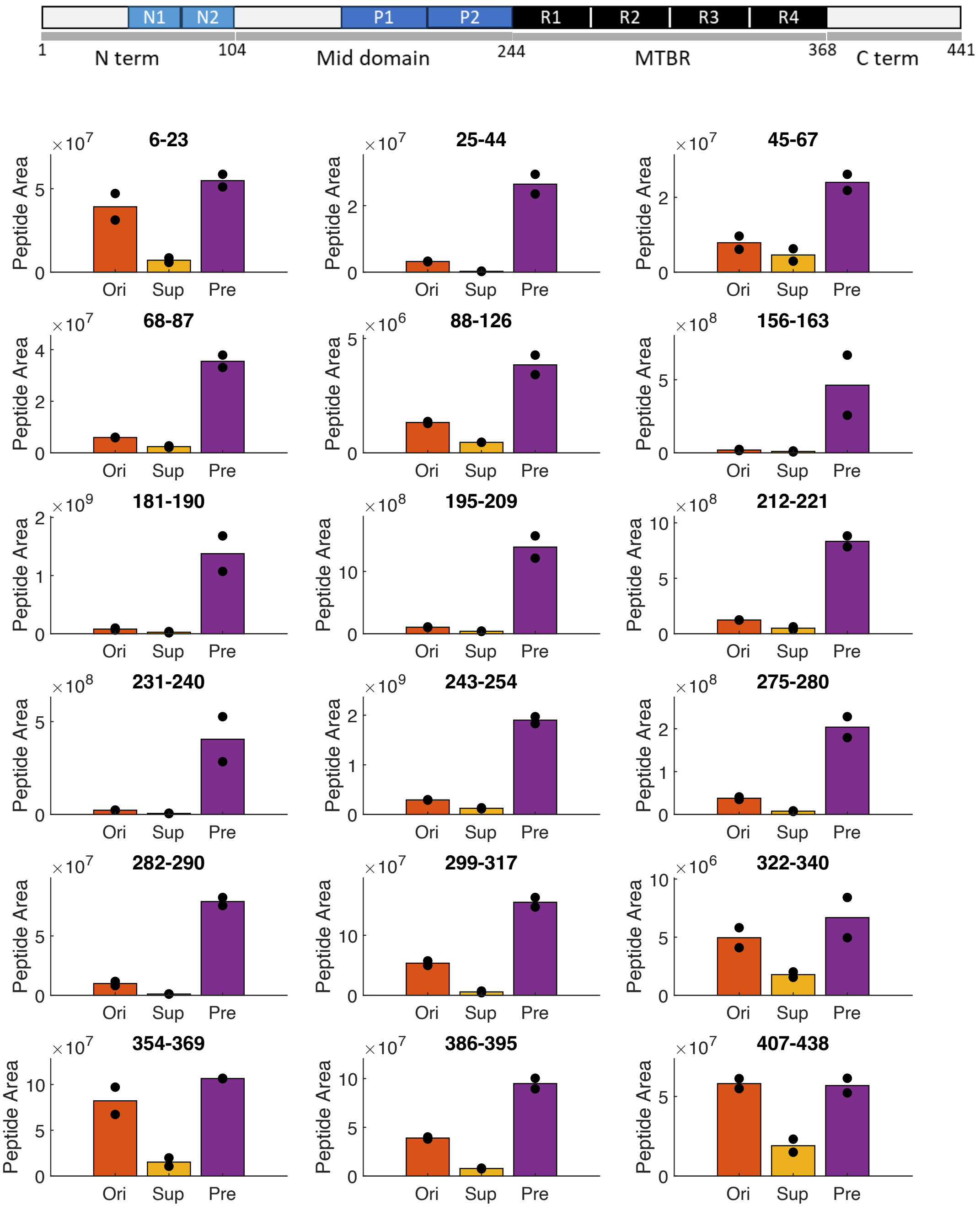

Supplementary Figure 6. The abundance of tau peptides in non-depleted original ("Ori"), depleted supernatant ("Sup"), and precipitate ("Pre") fractions in a pooled TBS soluble brain extract immunoprecipitated with BT2 (epitope: aa194-198). The peak area of each tau peptide, derived from a PRM assay on the Orbitrap Exploris™ 480, served as a surrogate for peptide abundance. The structural features of tau441 are illustrated at the top of the figure. Each bar graph's title indicates the start and end positions of the peptide in tau441. N=2 biological replicate IPs.

IP with Tau5 antibody

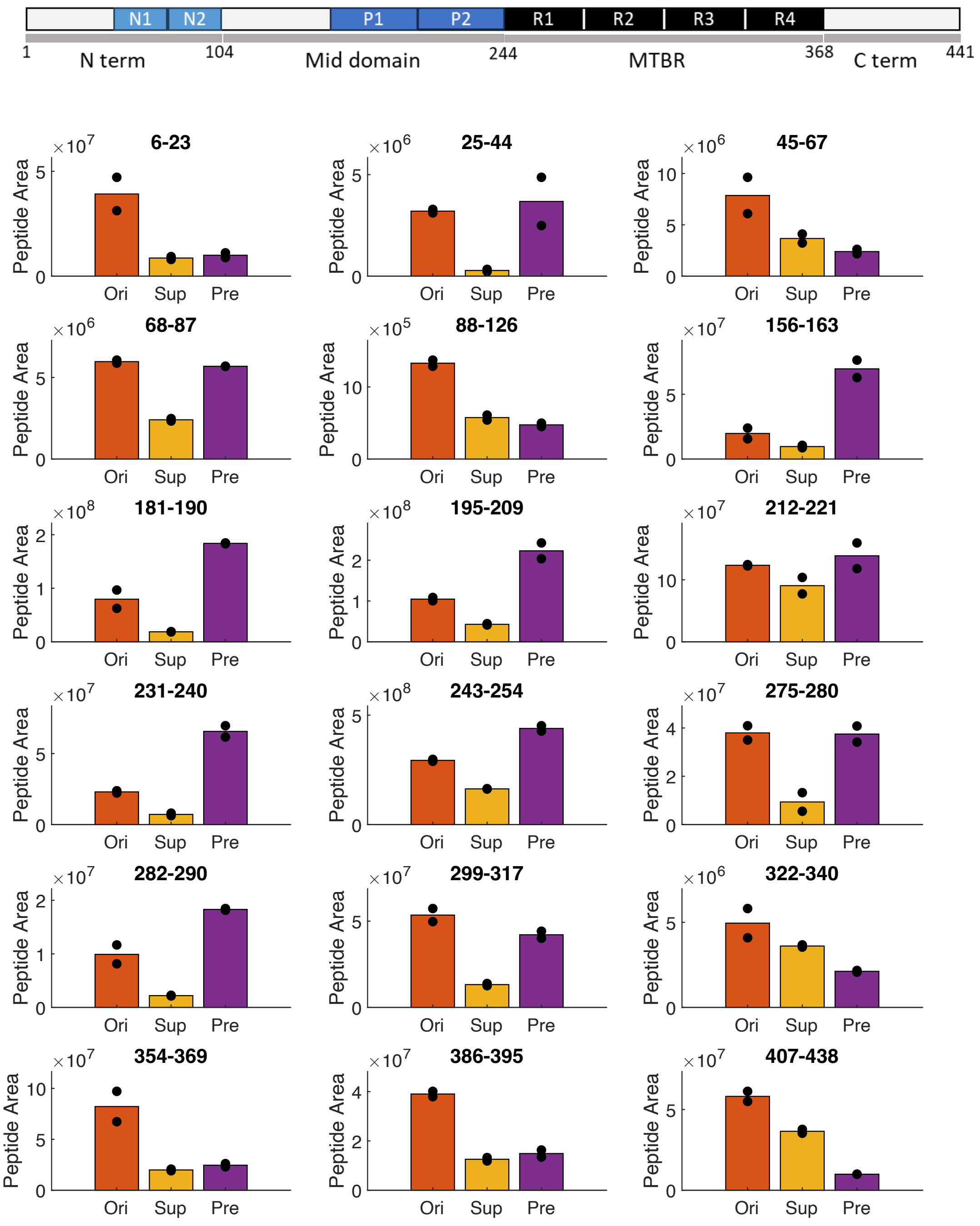

Supplementary Figure 7. The abundance of tau peptides in non-depleted original ("Ori"), depleted supernatant ("Sup"), and precipitate ("Pre") fractions in a pooled TBS soluble brain extract immunoprecipitated with the antibody Tau5 (epitope: 210-230). The peak area of each tau peptide, derived from a PRM assay on the Orbitrap Exploris™ 480, served as a surrogate for peptide abundance. The structural features of tau441 are illustrated at the top of the graph. Each bar graph's title indicates the start and end positions of the peptide in tau441. N=2 biological replicate IPs.

IP with 77G7 antibody

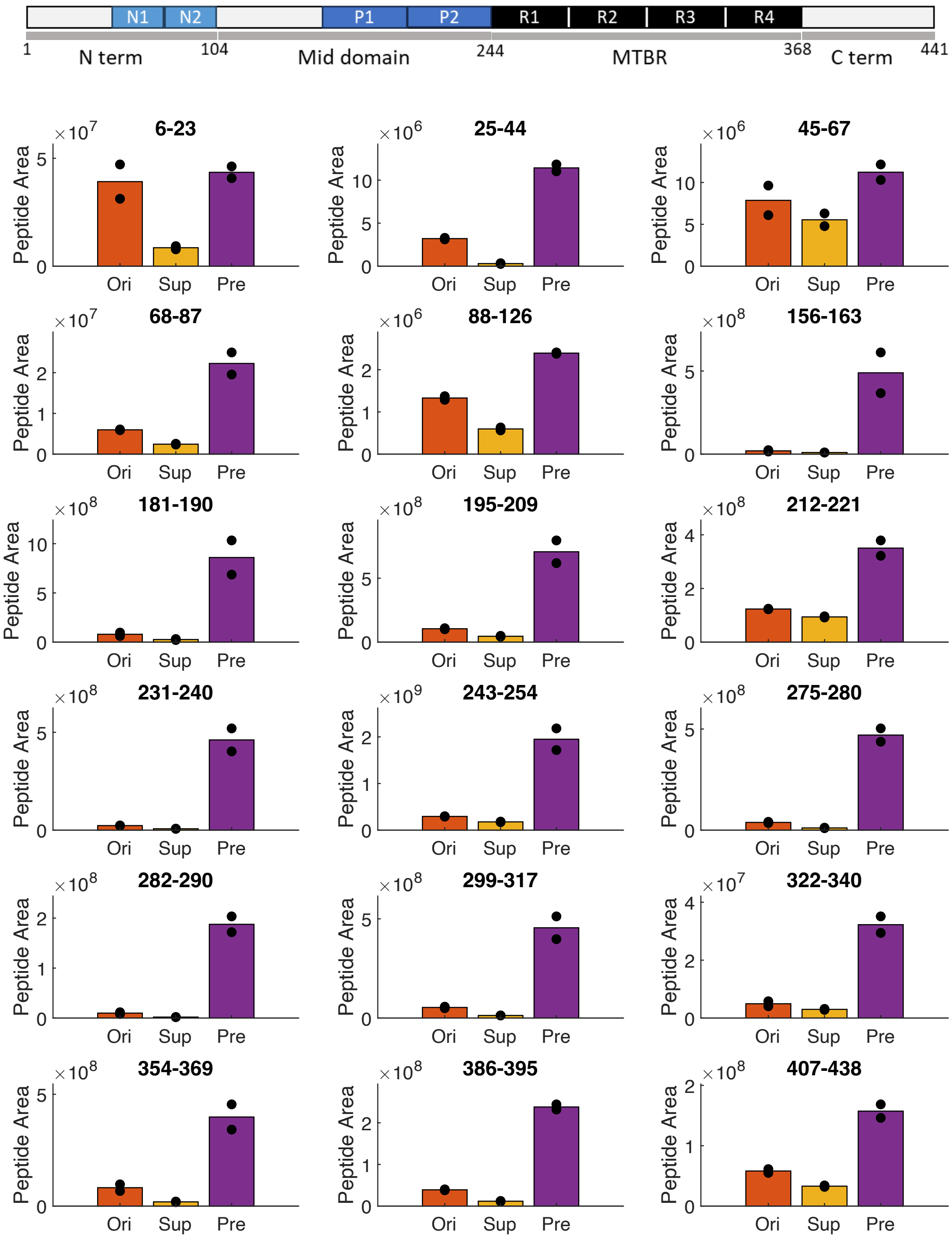

Supplementary Figure 8. The abundance of tau peptides in non-depleted original ("Ori"), depleted supernatant ("Sup"), and precipitate ("Pre") fractions in a pooled TBS soluble brain extract immunoprecipitated with the antibody 77G7 (epitope: 316-355). The peak area of each tau peptide, derived from a PRM assay on the Orbitrap Exploris™ 480, served as a surrogate for peptide abundance. The structural features of tau441 are illustrated at the top of the graph. Each bar graph's title indicates the start and end positions of the peptide in tau441. N=2 biological replicate IPs.

IP with Tau46 antibody

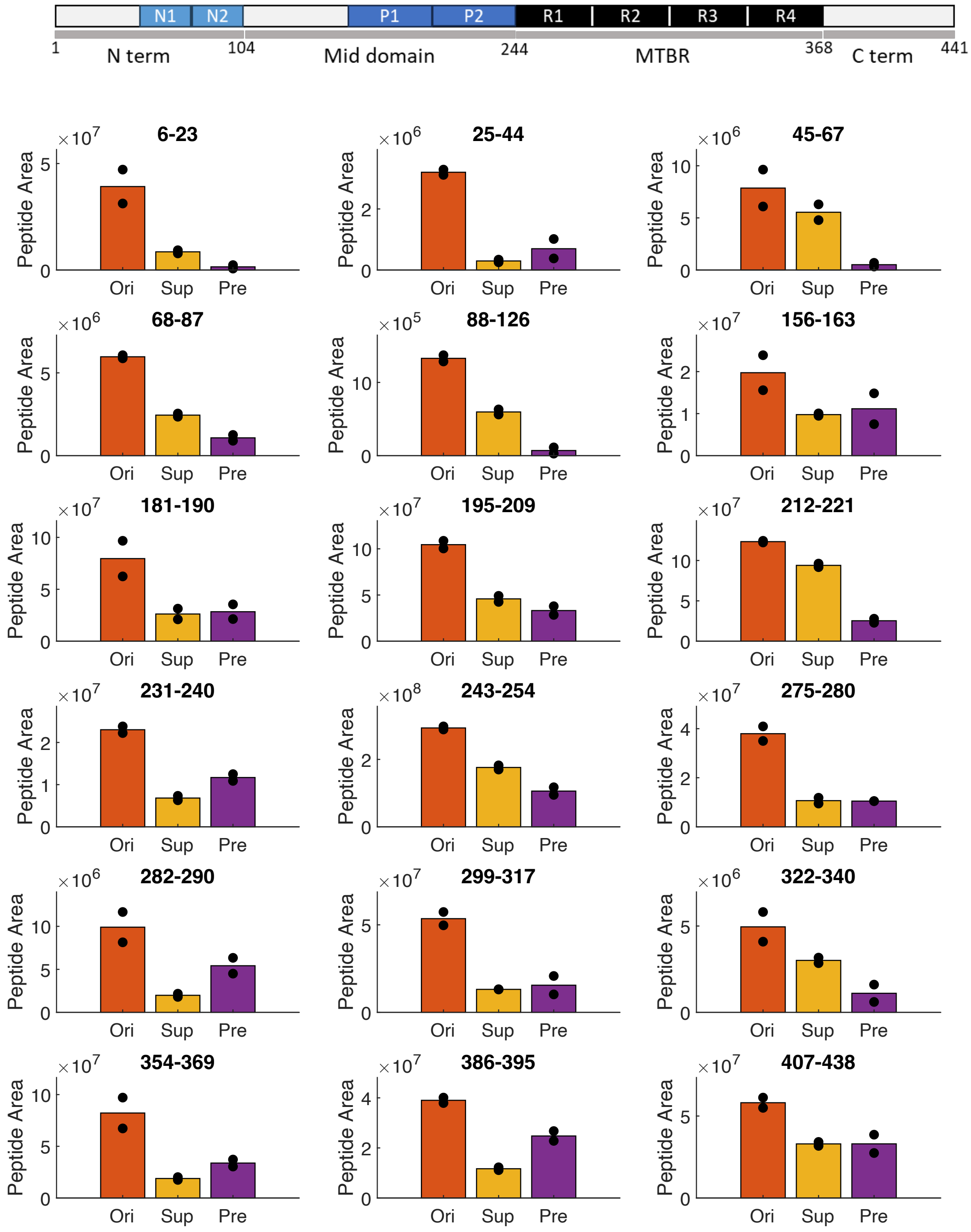

Supplementary Figure 9. The abundance of tau peptides in non-depleted original ("Ori"), depleted supernatant ("Sup"), and precipitate ("Pre") fractions in a pooled TBS soluble brain extract immunoprecipitated with the Tau46 antibody (epitope: 404-441). The peak area of each tau peptide, derived from a PRM assay on the Orbitrap Exploris™ 480, served as a surrogate for peptide abundance. The structural features of tau441 are illustrated at the top of the graph. Each bar graph's title indicates the start and end positions of the peptide in tau441. N=2 biological replicate IPs.

IP with mouse IgG isotype

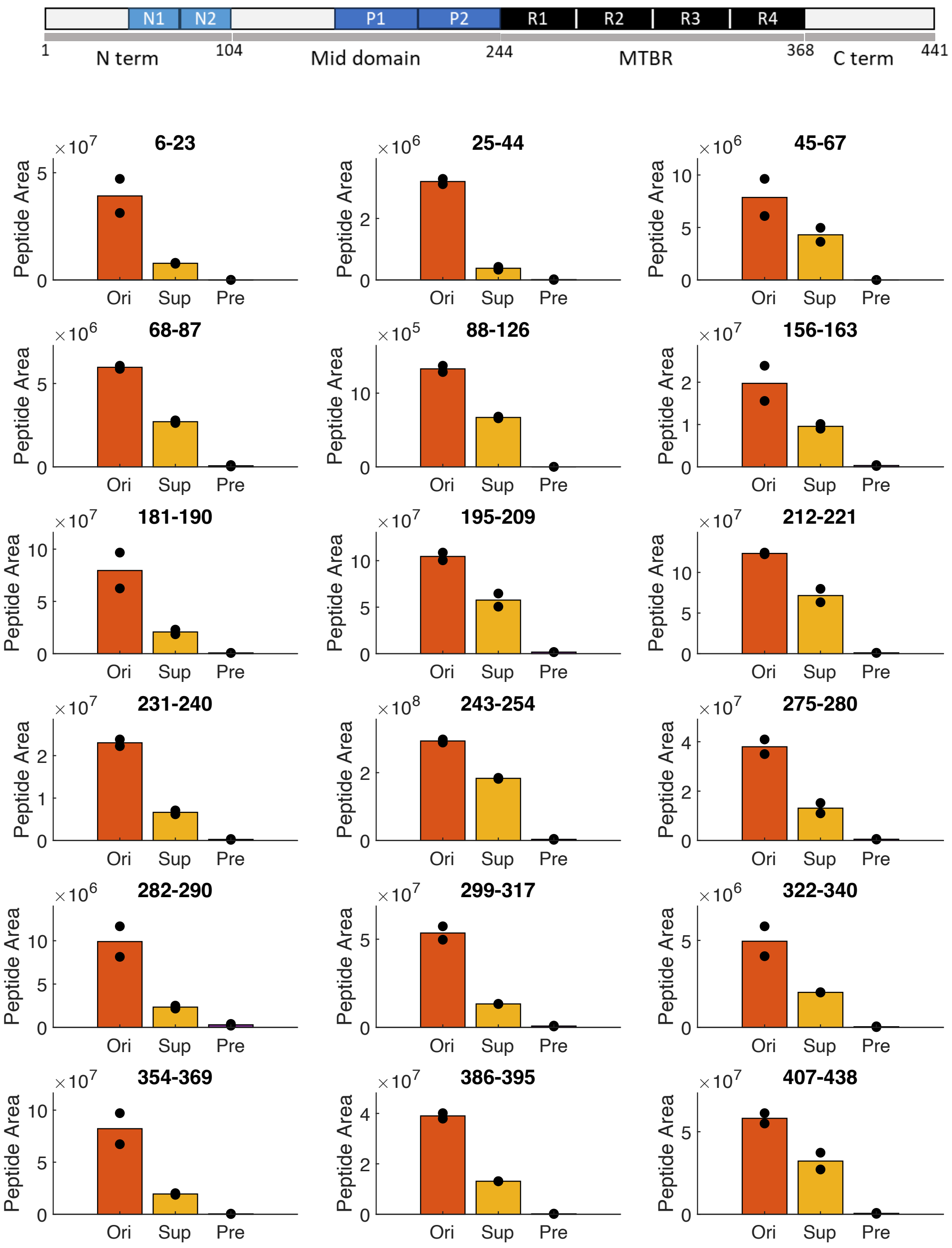

Supplementary Figure 10. The abundance of tau peptides in non-depleted original ("Ori"), depleted supernatant ("Sup"), and precipitate ("Pre") fractions in a pooled TBS soluble brain extract immunoprecipitated with a mouse IgG antibody not directed against tau protein. The peak area of each tau peptide, derived from a PRM assay on the Orbitrap Exploris™ 480, served as a surrogate for peptide abundance. The structural features of tau441 are illustrated at the top of the graph. Each bar graph's title indicates the start and end positions of the peptide in tau441. N=2 biological replicate IPs.

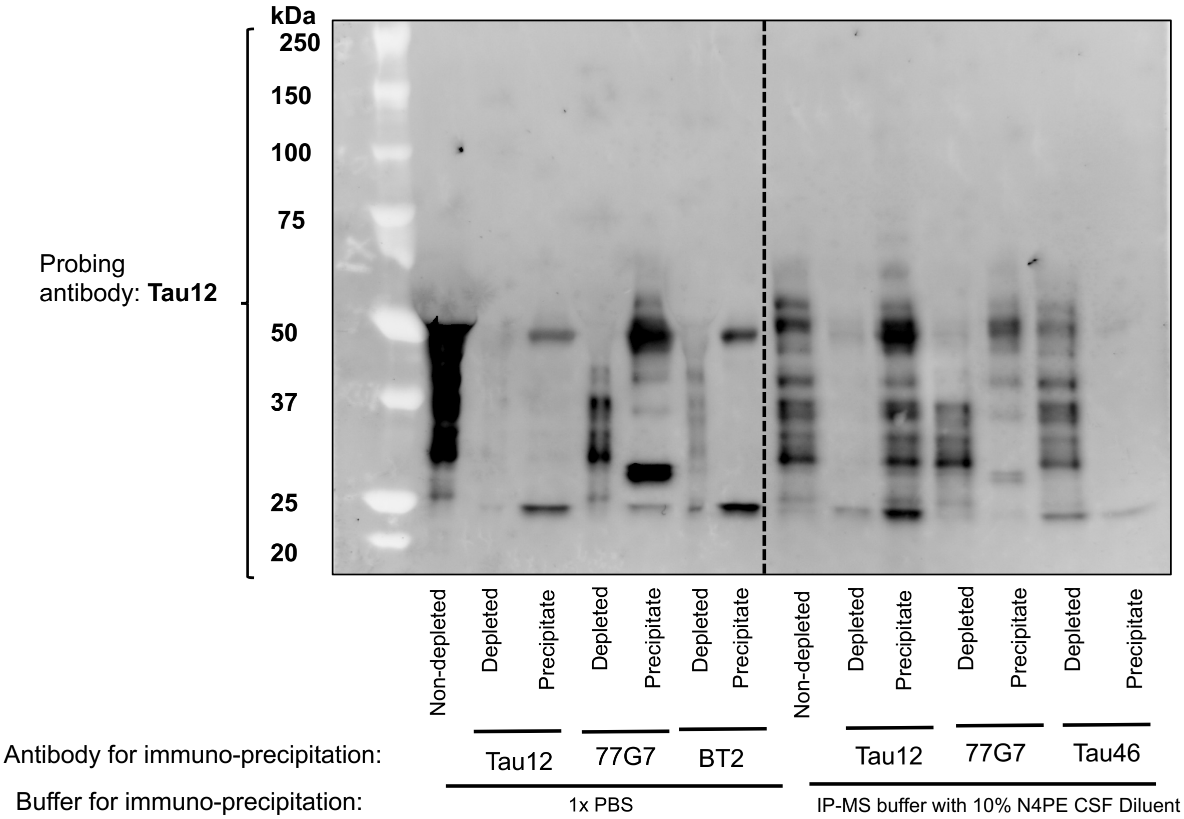

Supplementary Figure 11. Western blotting of non-depleted tau forms in TBS-soluble AD brain extracts as well as the depleted and precipitated fractions following immunoprecipitation with defined antibodies. The figure shows Western blotting with the antibody Tau12 against fractions before and after immunoprecipitation with Tau12, BT2, 77G7 and Tau46. Samples were immunoprecipitated either with PBS (as in Figure 2) or IP-MS buffer supplemented with 10% CSF sample diluent in the Neurology 4-plex kit from Quanterix Corp. (as in the IP-MS experiments in Extended Data Figure 1 and Supplementary Figures 3-9). Representative image from two technical replicates.

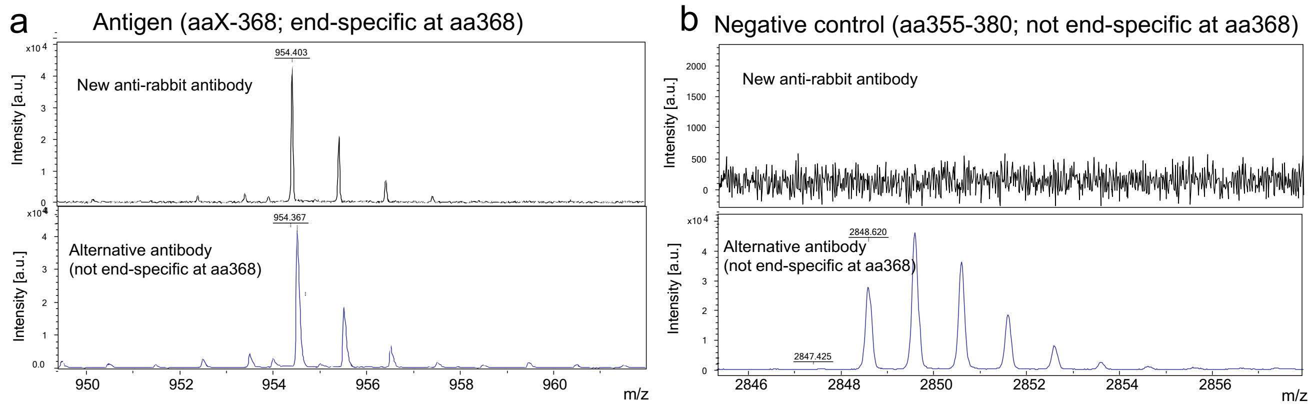

Supplementary Figure 12. Mass spectrometric validation of the end-specificity of the novel rabbit polyclonal antibody targeting tau truncated at aa368. The positive control (i.e., the antigen used to generate the antibody) and the negative control peptides were separately immunoprecipitated either with the new rabbit polyclonal antibody or with an alternative antibody that binds in the same region but is not end-specific for truncation at aa368. The figure shows MALDI spectra for the positive control/antigen (a) or the negative control (b) immunoprecipitated with the aa368 antibody (top panels) or the alternative antibody (bottom panel).

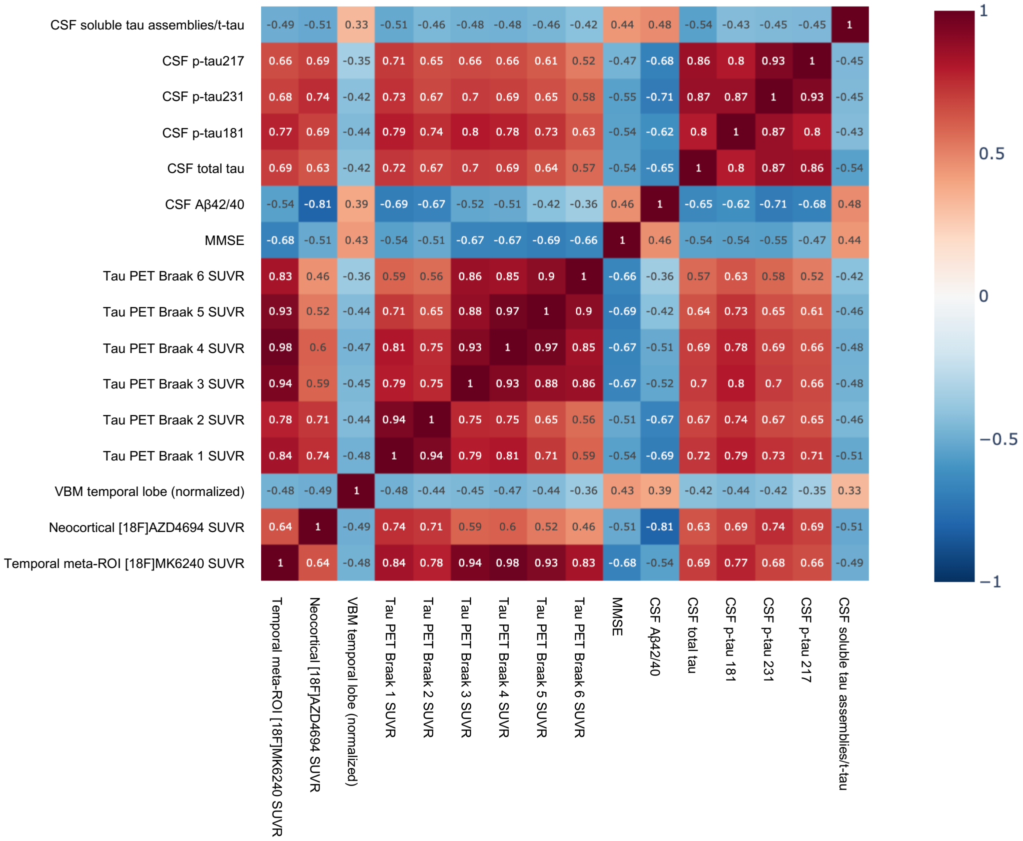

All values are significant (pFDR < 0.05)

Supplementary Figure 13. Pearson's correlation of CSF soluble tau assemblies (STAs)/total-tau ratio with regional tau PET uptake, CSF biomarkers, and cognitive performance (cohort 4). The strengths of the correlations are shown in a heatmap with intensity ranging from -1 to 1. The statistical tests were two-sided, and p-values were adjusted for multiple comparisons using the false discovery rate (FDR) correction.

Supplementary Tables

Supplementary Table 1: List of transitions used in the Parallel Reaction Monitoring (PRM) assays for various tau peptides in the IP-MS experiments.

Peptide amino acid sequence	Position	m/z	z	t start (min)	t stop (min)	Transitions for quantification
QEFEVMEDHAGTYGLGDR	6-23	1027.45	2	3.4	6.4	1421.611 (y13); 1161.528 (y11); 1046.501 (y10)
DQGGYTMHQDQEGDTDAGLK	25-44	722.64	3	2.3	5.3	905.421 (y9); 388.255 (y4); 961.913 ($y18^{2+}$)
ESPLQTPTEDGSEEPGSETSDAK	45-67	1196.02	2	2.5	5.5	1408.571 (y14); 1293.544 (y13); 891.405 (y9)
STPTAEDVTAPLVDEGAPGK	68-87	977.984	2	3.4	6.4	1154.605 (y12); 1053.558 (y11); 301.187 (y3)
QAAAQPHTEIPEGTTAEEAGIGDT PSLEDEAAGHVTQAR	88-126	1319.29	3	3.3	6.3	475.262 (y4); 790.887 ($y15^{2+}$); 1047.522 (b10)
GAAPPGQK	156-163	363.201	2	1.3	4.3	526.298 (y5); 429.246 (y4); 129.066 (b2)
TPPSSGEPPK	181-190	498.754	2	1.6	4.6	798.399 (y8); 341.218 (y3); 448.230 ($y9^{2+}$)
SGYSSPGSPGTPGSR	195-209	697.321	2	2	5	999.485 (y11); 912.453 (y10); 671.347 (y7)
TPSLPTPPTR	212-221	533.798	2	2.8	5.8	868.489 (y8); 668.373 (y6); 470.272 (y4)
TPPKSPSSAK	231-240	500.277	2	1.2	4.2	801.446 (y8); 576.300 (y6); 449.753 ($y9^{2+}$)
LQTAPVPMPDLK	243-254	655.363	2	3.7	6.7	896.491 (y8); 700.370 (y6); 472.277 (y4)
VQIINK	275-280	357.729	2	2.1	5.1	487.324 (y4); 374.240 (y3); 261.156 (y2)
LDLSNVQSK	282-290	502.275	2	2.6	5.6	775.431 (y7); 662.347 (y6); 362.203 (y3)
HVPGGGSVQIVYKPVDLSK	299-317	660.702	3	3	6	949.535 (y8); 658.377 (y6); 872.486 ($y17^{2+}$)
CGSLGNIHHKPGGGQVEVK	322-340	494.255	4	1.9	4.9	870.468 (y9); 435.738 ($y9^{2+}$); 519.614 ($y15^{2+}$)
IGSLDNITHVPGGGNK	354-369	789.915	2	2.9	5.9	866.448 (y9); 529.273 (y6); 951.489 (b9)
TDHGAEIVYK	386-395	566.785	2	2	5	458.748 (y8); 611.242 (b6); 724.326 (b7)
HLSNVSSTGSIDMVDSPQLATLAD EVSASLAK	407-438	1081.873	3	6.2	9.2	1275.679 (y13); 990.510 (y10); 1198.570 (b12)

All fragment ions were singly charged unless otherwise specified. B and y ions represent N-terminus and C-terminus fragment ions when the peptides were cleaved at the peptide bond. Underlined cysteine indicates carboxyamidomethylation.

Supplementary Table 2: List of transitions used in the Parallel Reaction Monitoring (PRM) assays for mass spectrometry characterization of phosphorylation in recombinant Tau441.

Peptide	Peptide amino acid sequence	Position	m/z	z	t start (min)	t stop (min)	Transitions for quantification
Pho_262	IGSTENLK	260 - 267	471.219	2	0	10	828.350 (y7); 604.330 (y5); 374.240 (y3)
Un_262	IGSTENLK	260 - 267	431.235	2	0	10	748.384 (y7); 691.362 (y6); 604.330 (y5)
Pho_356	IGSLDNITHVPGGGNK	353 - 369	553.600	3	0	10	866.448 (y9); 765.400 (y8); 529.273 (y6);
Un_356	IGSLDNITHVPGGGNK	353 - 369	526.944	3	0	10	866.448 (y9); 529.273 (y6); 733.373 ($y15^{2+}$)

All fragment ions were singly charged unless otherwise specified. B and y ions represent N-terminus and C-terminus fragment ions when the peptides were cleaved at the peptide bond. Underlined serine denotes carboxyamidomethylation.

Supplementary Table 3. Posthoc testing details for Figure 5f-h

Kruskal-Wallis statistics for Figure 5f-h

	Figure 5f	Figure 5g	Figure 5h
Kruskal-Wallis test P value	0.0065	<0.0001	<0.0001
Number of groups	5	5	5
Kruskal-Wallis statistic	14.27	24.6	25.66
Number of values (total)	60	60	60

Multiple comparisons for Figure 5f-h

**Dunn's multiple comparisons test**	**Figure 5f: RMP**		**Figure 5g: Input resistance**		**Figure 5h: Firing rate**	
	**Mean rank diff.**	**Adjusted P Value**	**Mean rank diff.**	**Adjusted P Value**	**Mean rank diff.**	**Adjusted P Value**
control vs. STA core	-20.46	0.04	-28.17	0.0008	-26.96	0.0016
control vs. N-terminus	-9.958	>0.9999	-2.417	>0.9999	-9.708	>0.9999
control vs. fibril core	-14	0.489	-11.21	>0.9999	-28.58	0.0006
control vs. C-terminus	2.125	>0.9999	2.625	>0.9999	-6.417	>0.9999
STA core vs. N-terminus	10.5	>0.9999	25.75	0.003	17.25	0.1552
STA core vs. fibril core	6.458	>0.9999	16.96	0.1736	-1.625	>0.9999
STA core vs. C-terminus	22.58	0.0149	30.79	0.0002	20.54	0.0395
N-terminus vs. fibril core	-4.042	>0.9999	-8.792	>0.9999	-18.88	0.081
N-terminus vs. C-terminus	12.08	0.8916	5.042	>0.9999	3.292	>0.9999
fibril core vs. C-terminus	16.13	0.233	13.83	0.5231	22.17	0.0187

Supplementary Table 4. Posthoc statistical testing information for Extended Data Tables 1-3

Extended Table 1 (post-hoc comparison p-values)

**Variable**	Control vs AD	Control vs Picks	Control vs CBD	Control vs PSP	AD vs Picks	AD vs CBD	AD vs PSP	Picks vs CBD	Picks vs PSP	CBD vs Picks
Age at onset	X	X	X	X	-	-	**0.0084**	-	**0.0084**	**0.025**
Age at death	-	-	-	-	-	-	-	-	0.05	0.05
Duration	X	X	X	X	-	-	-	-	-	-
Female sex	-	-	-	-	-	-	-	-	-	-
Brain weight	-	0.0032	-	-	-	-	-	-	-	-
APOE ε4 alleles: 0 / 1/ 2	-	X	X	X	X	X	X	X	X	X
Frontal t-tau	1.8×10^{-5}	1.8×10^{-5}	1.8×10^{-5}	1.8×10^{-5}	0.0043	1.8×10^{-5}	1.8×10^{-5}	-	1.9 x x 10^{-4}	6.1 x x 10^{-4}
Temporal t-tau	3.6×10^{-5}	3.6×10^{-5}	4.3×10^{-5}	1.3×10^{-4}	2.9×10^{-4}	4.3×10^{-5}	3.6×10^{-5}	-	0.0065	-
Soluble tau assemblies (FRET assay)	3.6×10^{-4}	8.2×10^{-4}	3.6×10^{-4}	0.0016	5.4×10^{-5}	5.4×10^{-5}	3.6×10^{-4}	-	-	0.0091

"-" = Not Significant
"X" = Cannot be tested

Extended Table 2 (post-hoc comparison p-values)

Variable	Low Path vs ADNC	Low Path vs Other Path	Low Path vs ADNC + Other	ADNC vs Other Path	ADNC vs ADNC + Other	Other Path vs ADNC + Other
Age at lumbar puncture	-	-	-	-	-	-
Age at death	-	-	-	-	-	-
CSF to death interval, years	-	-	-	-	-	-
Female	-	-	-	-	-	-
Hispanic	-	-	-	-	-	-
Years of education, mean ± SD	-	-	-	-	-	-
APOE ε4 alleles	-	-	-	-	-	-
DRS	-	-	-	-	-	-
CDR-sob	-	-	-	-	-	-
Clin Dx	0.012	0.012	0.012	0.012	-	0.012
Aβ42, pg/ml	0.036	-	0.036	0.045	-	0.045
Aβ40, pg/ml	-	-	-	-	-	-
Aβ42/40 ratio	0.0020	-	0.0018	0.0025	-	0.0018
t-tau, pg/ml	0.0099	-	0.0099	0.0099	-	0.0099
Soluble tau assemblies, pg/ml	-	-	-	-	-	-
Soluble tau assemblies/ t-tau	0.014	-	0.026	0.0052	-	0.0070
P-tau181, pg/ml *	0.0028	-	0.0028	2.3×10^{-4}	-	2.9×10^{-4}
P-tau231, pg/ml *	0.0012	-	0.0015	1.1×10^{-4}	-	2.5×10^{-4}
P-tau212, pg/ml *	3.2×10^{-4}	-	8.9×10^{-4}	1.00×10^{-05}	-	4.50×10^{-05}
P-tau217, pg/ml *	0.0033	-	0.014	3.4×10^{-4}	-	0.0031

"-" = Not Significant
"X" = Cannot be tested

Extended Table 3 (post-hoc comparison p-values)

Variable	Low Path vs ADNC	Low Path vs Other Path	Low Path vs ADNC + Other	ADNC vs Other Path	ADNC vs ADNC + Other	Other Path vs ADNC + Other
Age at death	-	-	-	-	-	-
Neuritic Plaques	4.5×10^{-4}	-	7.8×10^{-4}	7.8×10^{-5}	X	1.8×10^{-4}
Braak Stage	8.4×10^{-7}	-	1.7×10^{-6}	1.0×10^{-8}	X	1.4×10^{-8}
NIA-Reagan	8.4×10^{-7}	-	1.7×10^{-6}	1.0×10^{-8}	X	1.4×10^{-8}
CAA	-	-	-	-	-	-
LBD	-	-	0.0014	0.0014	8.7×10^{-6}	-
Hippocampal Sclerosis	X	-	-	-	-	-
FTLD	X	-	X	0.0080	X	0.0080
Other Pathology	X	X	-	X	-	-
Infarct	-	-	-	-	-	-
Microinfarct	-	-	-	-	-	-
Atherosclerosis	-	-	-	-	-	-

"-" = Not Significant
"X" = Cannot be tested

Supplementary Table 5. Antibodies used in the immunodepletion/immunoprecipitation studies.

Antibody	Species/ clonality	Clone name	Epitope	Source/ catalog number
Tau12	Mouse Mono	Tau 12	Tau amino acids 6-18	BioLegend 806501
95-108	Mouse Mono	SMI51	Tau amino acids 95-108	BioLegend 836104
HT7	Mouse Mono	HT7	Tau amino acids 159-163	Thermo MN1000
BT2	Mouse Mono	BT2	Tau amino acids 194-198	Thermo MN1010
Tau5	Mouse Mono	Tau5	Tau amino acids 210-230	BioLegend 806401
K9JA	Rabbit Poly	None	Tau microtubule binding region	DAKO Not available
77G7	Mouse Mono	77G7	Tau amino acids 316-355	BioLegend 817601
4R	Mouse Mono	5F9	The tau R2 region of the microtubule binding region	BioLegend 823701
368	Rabbit Poly	Not available	Tau amino acids ITHVPGGGN ending at N-368	This study
419	Rat Mono	A16097D	Tau amino acids 419-433	Bio Legend 851002
Tau46	Mouse Mono	Tau46	Tau amino acids 404-441	BioLegend 806601
TauAB	Mouse Mono	TauAB	Tau amino acids 425-441	MedImmune Chen et al., 2019, Alzheimers Dement 15(3):487-496
CT2	Mouse Mono	None	Tau amino acids 241-294	This study
CT3	Mouse Mono	None	Tau amino acids 281-331	This study
CT4	Mouse Mono	None	Tau amino acids 321-371	This study
CT1	Mouse Mono	None	Tau amino acids 361-411	This study
CT5	Mouse Mono	None	Tau amino acids 401-441	This study
P-tau181	Mouse Mono	AT180	Tau phosphorylation at threonine-181	Thermo, MN1040

P-tau202/205	Mouse<br>Mono	AT8	Tau phosphorylation at serine-202 and threonine-205	Thermo, MN1020
P-tau212	Rabbit<br>polyclonal	none	Tau phosphorylation at threonine-205	Thermo<br>44-740G
P-tau217	Rabit<br>polyclonal	none	Tau phosphorylation at threonine-217	Thermo<br>44-744
P-tau231	Mono<br>Mouse	none	Tau phosphorylation at threonine-231	ADx<br>ADx 253
P-tau235	Rabbit<br>Poly	None	Tau phosphorylation at serine-235	Thermo<br>PA5-104785
P-tau262	Rabbit<br>Poly	None	Tau phosphorylation at serine-262	Thermo, 44-750-G
P-tau356	Rabbit<br>Poly	None	Tau phosphorylation at serine-356	Thermo, 44-751G
P-tau396	Rabbit<br>Poly	None	Tau phosphorylation at serine-396	Thermo<br>44-752G
P-tau416	Rabbit<br>Poly	None	Tau phosphorylation at serine-416	Thermo<br>PA5-117246

Supplementary Table 6. Details of primary antibodies used in the immunohistochemistry and immunofluorescence studies.

Primary antibody	Species/ clonality	Clone name	Source, catalog number
P-tau181	Mouse Mono	AT180	Thermo, MN1040
P-tau202/p-tau205	Mouse Mono	AT8	Thermo, MN1020
P-tau262	Rabbit Poly	None	Thermo, 44-750-G
P-tau356	Rabbit Poly	None	Thermo, 44-751G
