# Supplementary material for: Phospho-tau serine-262 and serine-356 as biomarkers of pre-tangle soluble tau assemblies in Alzheimer’s disease
Source: Nat Med. 2025 Feb 10;31(2):574–88. doi: 10.1038/s41591-024-03400-0 (PMC11835754; doi:10.1038/s41591-024-03400-0)
Supplement: Supplementary file 2 — Reporting Summary [file 41591_2024_3400_MOESM2_ESM.pdf]

nature portfolio

Corresponding author(s): Thomas K. Karikari

Last updated by author(s): 10/17/2024

Reporting Summary

Nature Portfolio wishes to improve the reproducibility of the work that we publish. This form provides structure for consistency and transparency in reporting. For further information on Nature Portfolio policies, see our Editorial Policies and the Editorial Policy Checklist.

Statistics

For all statistical analyses, confirm that the following items are present in the figure legend, table legend, main text, or Methods section.

n/a	Confirmed	
☐	☒	The exact sample size (n) for each experimental group/condition, given as a discrete number and unit of measurement
☐	☒	A statement on whether measurements were taken from distinct samples or whether the same sample was measured repeatedly
☐	☒	The statistical test(s) used AND whether they are one- or two-sided<br>*Only common tests should be described solely by name; describe more complex techniques in the Methods section.*
☐	☒	A description of all covariates tested
☐	☒	A description of any assumptions or corrections, such as tests of normality and adjustment for multiple comparisons
☐	☒	A full description of the statistical parameters including central tendency (e.g. means) or other basic estimates (e.g. regression coefficient) AND variation (e.g. standard deviation) or associated estimates of uncertainty (e.g. confidence intervals)
☐	☒	For null hypothesis testing, the test statistic (e.g. F, t, r) with confidence intervals, effect sizes, degrees of freedom and P value noted<br>*Give P values as exact values whenever suitable.*
☒	☐	For Bayesian analysis, information on the choice of priors and Markov chain Monte Carlo settings
☒	☐	For hierarchical and complex designs, identification of the appropriate level for tests and full reporting of outcomes
☐	☒	Estimates of effect sizes (e.g. Cohen's d, Pearson's r), indicating how they were calculated

Our web collection on statistics for biologists contains articles on many of the points above.

Software and code

Policy information about availability of computer code

Data collection	Olympus BX53 microscope connected to a fluorescence illuminator (X-cite 120Q) and equipped with an Olympus DP72 digital camera, IR-DIC optics with an Olympus BX151W microscope (Scientifica) and a CCD camera (Hitachi), Axon Multiclamp 700B amplifier (Molecular Devices) , Talos L120C 120 kV TEM microscope (Thermo Fisher), BIAcore T100 biosensor (GE Healthcare), VECTASTAIN Elite ABC-HRP kit (Vector Laboratories, Burlingame, CA), Micro CED (Mark 2), Orbitrap Exploris 480 Mass Spectrometer coupled to Dionex Ultimate 3000 HPLC system (Thermo Scientific, Waltham, MA), Siemens High Resolution Research Tomograph (Siemens Medical Solutions, Knoxville, TN, USA), JEOL Flash 1400 TEM (JEOL USA, Inc., Peabody, MA), Bio-Rad ChemiDoc MP (Bio-Rad, Hercules, California).
Data analysis	GraphPad Prism version 9, Olympus cell Sens Standard imaging software (version 1.12), pClamp 10 (Molecular Devices), MATLAB (MathWorks), Julia (Julia Hub, version 1.7.3), Spike software (version 6.1, Cambridge Electronic Design, Cambridge UK), R version 4.3.1, Python 3.11.2, Pandas (version 1.5.3), NumPy (version 1.24.2), Scikit-learn (Version 1.2.2), Statsmodels (Version 0.13.5), VoxelStats toolbox, Xcalibur 2.2 SP1.48 (Thermo Scientific, Waltham, MA), Skyline (version 21.2.0.568).

For manuscripts utilizing custom algorithms or software that are central to the research but not yet described in published literature, software must be made available to editors and reviewers. We strongly encourage code deposition in a community repository (e.g. GitHub). See the Nature Portfolio guidelines for submitting code & software for further information.

Data

Policy information about availability of data

All manuscripts must include a data availability statement. This statement should provide the following information, where applicable:

- Accession codes, unique identifiers, or web links for publicly available datasets
- A description of any restrictions on data availability
- For clinical datasets or third party data, please ensure that the statement adheres to our policy

The Uniprot database was used to search for tau protein sequence (Uniprot ID: P10636). De-identified data generated in this study can be shared with qualified and identifiable investigators for the purpose of replicating the results and procedures in the study. Requests can be made to the corresponding author of the present study (TKK) who will refer to the respective cohort principal investigators where necessary. Requests will be reviewed by the investigators and respective institutions to ensure that data request and transfer is in agreement with UK, EU and USA legislation on general data protection or is subject to any intellectual property or confidentiality obligations. The purpose of these procedures is to ensure participant anonymity and ensure data safeguarding limited to the terms set forth in the IRB approvals. Data request to the TRIAD, Pittsburgh ADRC and the UCSD cohorts can be made directly at https://datashare.tnl-mcgill.com/ds/, https://www.adrc.pitt.edu/for-researchers/adrc-data-resources/ and https://neurosciences.ucsd.edu/centers-programs/adrc/professionals/resources/index.html, respectively.

Research involving human participants, their data, or biological material

Policy information about studies with human participants or human data. See also policy information about sex, gender (identity/presentation), and sexual orientation and race, ethnicity and racism.

Reporting on sex and gender	"Sex" referred to biological sex, based on self-reports, and this information is provided in the demographic tables. Sex was used as a covariate in statistical analyses for the clinical cohort studies. Sex distribution has been indicated in the cohort characteristics (Extended Data Tables 1, 2 and 4).
Reporting on race, ethnicity, or other socially relevant groupings	Self-reported racial status (Hispanic or non-Hispanic) has been presented for the blood-to-autopsy cohort (cohort 3) in Extended Table 2. No other race, ethnicity or socially relevant grouping information is presented in the manuscript.
Population characteristics	Detailed human cohort characteristics information are given in Extended Data Tables 1-4.
Recruitment	The UCSD ADRC cohort mostly included participants reporting with memory complaints to the Alzheimer's Disease Research Center. The TRIAD cohort recruited both cognitively normal and cognitively impaired participants from a variety of sources. Inclusion of participants in the catchment area begins with a telephone screening to ensure eligibility, followed by informed consent signing and detailed clinical and biomarker assessments. The Netherlands Brain Bank, the Queen Square Brain Bank and the University of Pittsburgh's ADRC Brain Bank recruited participants for brain donation based on their willingness and availability. These factors could have biased participation based on several factors including proximity to the study sites, language barriers and willingness to undergo the specified evaluations. These factors could impact the widespread transferability of results unless they have been validated in other, more diverse cohorts.
Ethics oversight	All participants or their close relatives/next of kin provided written informed consent. The Queen Square Brain Bank (QSBB) has generic ethical approval from a London Multi-Centre Research Ethics Committee under a license from the Human Tissue Authority. The Netherlands Brain Bank cohort was approved by the ethics committee of the VU University Medical Center (Amsterdam, the Netherlands). The research protocol for the University of California San Diego (UCSD) cohort was reviewed and approved by the human subject review board at UCSD, while informed consent was obtained from all patients or their caregivers consistent with California State law. Hippocampal tissue samples obtained from autopsy cases in the University of Pittsburgh Alzheimer's Disease Research Center's brain bank were approved by the University of Pittsburgh Committee for Oversight of Research and Clinical Training Involving Decedents. The TRIAD study was approved by the Montreal Neurological Institute PET working committee and the Douglas Mental Health University Institute Research Ethics Board.

Note that full information on the approval of the study protocol must also be provided in the manuscript.

Field-specific reporting

Please select the one below that is the best fit for your research. If you are not sure, read the appropriate sections before making your selection.

☒ Life sciences ☐ Behavioural & social sciences ☐ Ecological, evolutionary & environmental sciences

For a reference copy of the document with all sections, see nature.com/documents/nr-reporting-summary-flat.pdf

Life sciences study design

All studies must disclose on these points even when the disclosure is negative.

Sample size	No a priori sample size calculation was done. Human samples were selected and used based on specimen availability and details of their associated clinical and biomarker data. Sample size for the electrophysiology experiments were informed by our previous studies (e.g., Hill et

	al., eneuro, 2019, Hill et al., Communication Biology 2021).
Data exclusions	All participants with available brain tissue or cerebrospinal fluid samples were included. One participant in the neuropathology cohort 1 was excluded due to lack of brain tissue for analysis.
Replication	The neuropathology studies (Tris buffered saline-soluble homogenates) and the cerebrospinal fluid studies were each replicated in two independent cohorts. Other experiments were replicated at least twice owing to sample availability, with high reproducibility. Replicate values have been shown as separate data points in figures and extended data figures.
Randomization	This is an observational study. Therefore, randomization was not necessary.
Blinding	Biomarker measurements were performed by scientists blinded to the clinical and demographic data of the participants. In mouse electrophysiology experiments, the scientists were blinded to the identity of tau constructs being examined. Immunohistochemical and immunofluorescence experiments were performed blinded to Braak stage and imaging/analysis was performed blinded to Braak stage and to the antibodies used to achieve the staining. The other experiments were performed without blinding.

Reporting for specific materials, systems and methods

We require information from authors about some types of materials, experimental systems and methods used in many studies. Here, indicate whether each material, system or method listed is relevant to your study. If you are not sure if a list item applies to your research, read the appropriate section before selecting a response.

Materials & experimental systems

n/a	Involved in the study
☐	☒ Antibodies
☒	☐ Eukaryotic cell lines
☒	☐ Palaeontology and archaeology
☐	☒ Animals and other organisms
☒	☐ Clinical data
☒	☐ Dual use research of concern
☒	☐ Plants

Methods

n/a	Involved in the study
☒	☐ ChIP-seq
☒	☐ Flow cytometry
☒	☐ MRI-based neuroimaging

Antibodies

Antibodies used

The following antibodies were used for the WES/Western blotting, immunoprecipitation/immunodepletion and immunohistochemistry experiments:
- Tau12 from BioLegend, catalog number 806501
-Tau95-108 from BioLegend, atalog number 836104
- HT7 from Thermo Fisher, catalog number MN1000
- BT2 from Thermo Fisher, catalog number MN1010
- Tau5 from BioLegend, catalog number 806401
- K9JA from DAKO, catalog number unavailable (discontinued)
- 777G7 from BioLegend, catalog number 817601
- 4R tau (clone 5F9) from BioLegend, catalog number 823701
- Tau 368, generated as part of this study
- Tau 419 (clone A16097D) from Bio Legend, catalog number 851002
- Tau46 from BioLegend, catalog number 806601
- Tau AB from MedImmune (Chen et al., 2019, Alzheimers Dement 15(3):487-496)
- CT1, 2, 3, 4 and 5 antibodies generated as part of this study
- P-tau181 from Thermo Fisher, catalog number MN1040
- P-tau202/205(AT8) from Thermo Fisher, catalog number MN1020
- P-tau212 from Thermo Fisher, catalog number 44-740G
- P-tau217 from Thermo Fisher, catalog number 44-744
- P-tau231 from ADx Neurosciences, catalog number ADx 253
- P-tau235 from Thermo Fisher, catalog number PA5-104785
- P-tau262 from Thermo Fisher, catalog number 44-750-G
- P-tau356 from Thermo Fisher, catalog number 44-751G
- P-tau396 from Thermo Fisher, catalog number 44-752G
- P-tau416 from Thermo Fisher, catalog number PA5-117246
- TauAB, a non-commercial antibody, was provided kind courtesy of MedImmune.
- Cy3 goat anti-mouse antibody (Jackson Immunoresearch, catalog number 15-165-166)
- Biotinylated goat anti-mouse immunoglobulin (Jackson Immunoresearch, West Grove, PA, 115-065-146, lot 156758)
- Biotinylated goat anti-rabbit immunoglobulin (Jackson Immunoresearch,, 111-065-045, lot 154094)
- Alexa-594 conjugated goat anti-mouse immunoglobulin (Jackson, 115-585-146, lot 157009)
- Alexa-488 conjugated goat anti-rabbit immunoglobulin (Jackson, 111-585-144, lot 157632).

Validation

The CT1, CT2, CT3, CT4, CT5 and tau368 antibodies were validated in the present study as well as in previous publications (e.g., Islam et al., 2024 Alzheimer's and Dementia). The other antibodies have been validated previously - Karikari et al 2020 Lancet Neurol; Karikari et al 2021 Alzheimer's and Dementia; Ashton et al 2021 Acta Neuropathologica; Lantero-Rodriguez et al., 2022 EMBO Mol

Med; Chen et al., 2019 Alzheimer's and Dementia; Montoliu-Gaya et al 2023 Nature Aging, and Ercan et al., 2017. Further sources of validation have been provided by the vendors on their webpages for the respective antibodies.

Animals and other research organisms

Policy information about studies involving animals; ARRIVE guidelines recommended for reporting animal research, and Sex and Gender in Research

Laboratory animals	C57BL/6 mice (3–4-weeks old), Balb/c mice (8 weeks old)
Wild animals	The study did not involve wild animals
Reporting on sex	Both male and female mice were used for the study. The study was not powered to assess sex differences between male and female mice.
Field-collected samples	The study did not involve samples collected from the field.
Ethics oversight	Animal care and experimental procedures in the electrophysiology experiments were reviewed and approved by the institutional animal welfare and ethical review body (AWERB) at the University of Warwick.

Note that full information on the approval of the study protocol must also be provided in the manuscript.

Plants

Seed stocks	*Report on the source of all seed stocks or other plant material used. If applicable, state the seed stock centre and catalogue number. If plant specimens were collected from the field, describe the collection location, date and sampling procedures.*
Novel plant genotypes	*Describe the methods by which all novel plant genotypes were produced. This includes those generated by transgenic approaches, gene editing, chemical/radiation-based mutagenesis and hybridization. For transgenic lines, describe the transformation method, the number of independent lines analyzed and the generation upon which experiments were performed. For gene-edited lines, describe the editor used, the endogenous sequence targeted for editing, the targeting guide RNA sequence (if applicable) and how the editor was applied.*
Authentication	*Describe any authentication procedures for each seed stock used or novel genotype generated. Describe any experiments used to assess the effect of a mutation and, where applicable, how potential secondary effects (e.g. second site T-DNA insertions, mosiacism, off-target gene editing) were examined.*
